# Electrophilic and Radical Ability of Organic Nitrating Reagents

**DOI:** 10.1021/acs.joc.5c02809

**Published:** 2026-02-18

**Authors:** Anthony J. Fernandes, Harry Lecomte, Dmitry Katayev

**Affiliations:** Department für Chemie und Biochemie, 27210 Universität Bern, Freiestrasse 3, Bern 3012, Switzerland

## Abstract

Aromatic nitration remains one of the most fundamental yet continuously evolving transformations in organic chemistry. While traditional “mixed-acid” systems rely on *in situ* generation of the nitronium ion under strongly acidic conditions, modern reagent design has shifted toward discrete, stable, and tunable NO_2_-transfer reagents that operate under milder and more selective conditions. Here, we report a computationally derived Nitro Plus Detachment (NPD) scale that quantifies the thermodynamic propensity of over 150 organic nitrating reagents to release nitronium ions. Systematic density functional theory (DFT) calculations across major structural classes—including *N*-nitro carboxamides and carboximides, azoles, azines, sulfonamides and sulfonimides, sulfoximines, and heteroatom- and carbon-based reagents—reveal clear linear correlations between NPD values, Hammett substituent constants, and experimentally observed reactivity. Electron-withdrawing groups and cationic frameworks are shown to dramatically enhance nitronium character. In addition, we introduce a complementary Nitro Radical Activation (NRA) scale that captures redox behavior relevant to emerging radical nitration strategies under photoredox conditions. Together, these two scales establish a unified thermodynamic and redox framework for predicting the performance of nitrating reagents and guiding the rational design of next-generation nitrating reagents and transformations.

## Graphical abstract

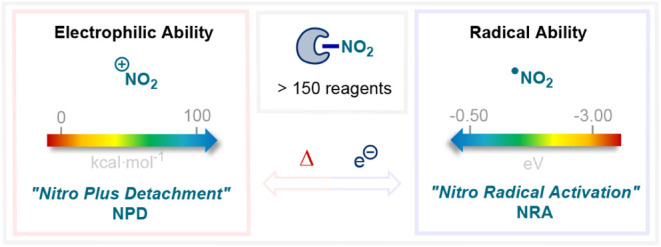

## Introduction

Aromatic nitration is one of the most thoroughly studied reactions in organic chemistry.^1^ This canonical transformation is a staple of chemistry textbooks, and the mechanism—formulated by Ingold and Hughes in the 1940s—was instrumental in establishing the general principles of electrophilic aromatic substitution.^2–5^ The nitronium ion is the key electrophile in nitration chemistry and is typically generated under strongly acidic conditions. Traditionally, aromatic nitration has been carried out using a mixture of nitric and sulfuric acids, the so-called “mixed-acid” nitration (Figure 1A). These conditions, however, suffer from major limitations, including poor functional group tolerance, problematic waste management, and significant safety and environmental concerns. Although some of these drawbacks can be mitigated by modern technologies such as flow chemistry,^6,7^ the field has particularly benefited over the past century from the development of more stable and readily available nitrating reagents (Figure 1B).^8^

**Figure 1:**
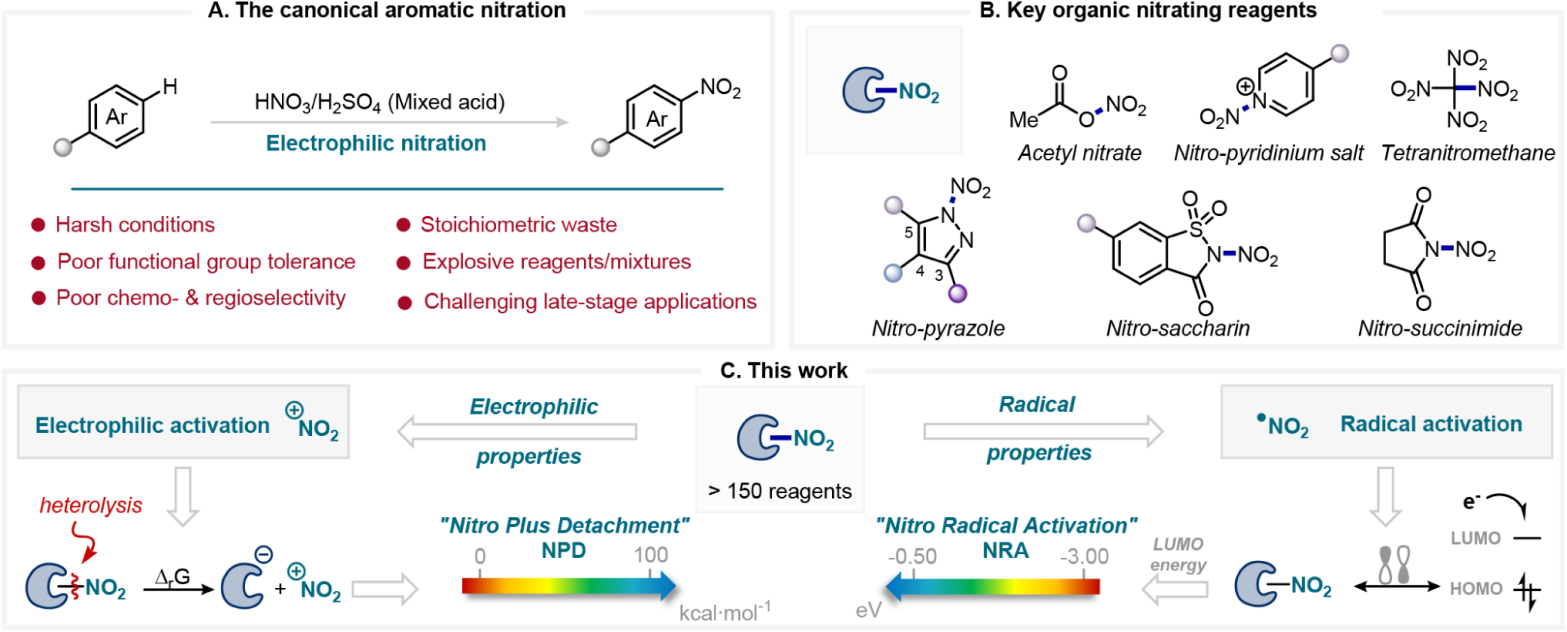
A) Aromatic nitration under mixed acid conditions. B) Key organic nitrating reagents developed in nitration chemistry. C) This work: Development of the NPD and NRA scales for quantifying the electrophilic and radical abilities of nitrating reagents.

The design of novel organic reagents has progressively enabled the development of electrophilic nitration reactions under milder conditions, thereby allowing for more selective and generally applicable transformations, including late-stage functionalization.^9,10^ For instance, acetyl nitrate—prepared *in situ* from nitrate salts and acetic anhydride—was already employed in the early 1900s as a convenient nitronium source. Tetranitromethane, an isolable yet explosive liquid, has been employed in the nitration of phenols and has rapidly been adopted for the nitration of tyrosyl residues in proteins.^11,12^ Subsequent work by Olah and coworkers introduced crystalline, solid nitro-pyridinium reagents,^13^ although their high moisture sensitivity limited their practical applications. Continued innovation has since yielded several new classes of solid and more stable nitrating agents, including *N*-nitro pyrazoles,^14,15^ *N*-nitro saccharin,^9,16^ and *N*-nitro succinimide,^17,18^ which offer tunable nitrating ability depending on the scaffold substitution pattern and reaction conditions. Taken together, these reagents illustrate two important trends in modern nitration chemistry: 1) a move away from highly acidic, wasteful mixed-acid protocols toward discrete, well-defined, and isolable NO_2_-transfer reagents that operate under milder and more chemoselective conditions, and 2) the design of organic reagents that balance stability with sufficient electrophilicity to achieve efficient nitro group transfer.

Besides electrophilic activation, recent years have witnessed a paradigm shift toward radical nitration transformations, driven by the remarkable progress of photoredox catalysis over the past two decades.^19–23^ These strategies rely on single-electron-transfer (SET) reduction of nitrating reagents by an excited photocatalyst, followed by mesolytic cleavage of the resulting radical anion to release nitryl radicals.^18,24,25^ Consequently, the redox properties of nitrating reagents—in particular, the ease of their reduction—are critical parameters governing their reactivity in such processes. Our group has a long-standing interest in nitration chemistry,^9,18^ and we have reported a series of efficient electrophilic and radical nitration reactions,^18,25^ often based on recyclable saccharin scaffolds, both in solution and using mechanochemistry, with the general aim of advancing the efficiency and sustainability of nitration processes.^26–28^ In the course of our studies on electrophilic aromatic nitration, we have employed the heterolytic dissociation energy of our nitrating reagents to rationalize their differences in reactivity.^9^ While previous studies have reported heterolytic and homolytic bond dissociation energies for selected nitrating reagents,^29^ these data remain limited in scope, and a general predictive framework is still lacking.

To address this gap, we have computationally determined the nitrating ability of more than 150 nitrating reagents—encompassing both known and unreported derivatives—across multiple reagent classes (Figure 1C). Inspired by the highly valuable Fluorine Plus Detachment (FPD) reactivity scale already available for fluorinating reagents,^30^ and analogous scales for trifluoromethylating^31^ and trifluoromethylthiolating^32^ reagents, we herein introduce the Nitro Plus Detachment (NPD) scale and validate its predictive power against Hammett σ parameters^33^ and previously reported experimental data. Furthermore, considering the recent progress and interest in radical nitration chemistry, we also present a complementary Nitro Radical Activation (NRA) scale.

## Methods

The DFT calculations were performed using the Gaussian 09 program package.^34^ The conformational space of all molecules was initially searched using meta-dynamics simulations based on tight-binding quantum chemical calculations, as implemented in the software package Conformer-Rotamer Ensemble Sampling Tool (CREST).^35^ The structures located with CREST were subjected to geometry optimization using the M06-2X functional^36,37^ with the 6-311++G­(2d, p) basis set^38,39^ (or def2-TZVP^40,41^ for molecules containing an iodine atom), including D3 dispersion correction^42^ and the polarizable continuum model (PCM)^43^ with SMD solvation parameters^44^ to account for solvent effects (acetonitrile). The nature of all stationary points was verified through the computation of vibrational frequencies. Single-point energies from these geometries were computed at the M06-2X/[6-311++G­(2df, 2p)+def2-QZVPPD­(Se, Br, I)] level, which has previously demonstrated high accuracy for dissociation energy predictions in related studies.^30–32^ These single-point calculations included D3 dispersion correction and SMD solvation (acetonitrile). Thermal corrections to Gibbs free energies were obtained from the optimized geometries and combined with single-point electronic energies to yield Gibbs free energies (ΔG) at 298.15 K. Frontier orbital energies were obtained at the SP level of theory.

The NPD scale was defined as the Gibbs free energy change (Δ_r_G) associated with the heterolytic cleavage of the Y–NO_2_ bond in the nitrating reagent, as shown in (Figure 1C, left). Therefore, high values indicate strong Y–NO_2_ bonds and, consequently, low nitrating ability, while low values express weak bonds and high nitrating power. As a representative example, NO_2_^+^BF_4_–^45^ exhibits an extremely low NPD of −6.6 kcal·mol^–1^ (Figure 2). Unless otherwise stated, all NPD values discussed throughout this work are reported in kcal·mol^–1^.

**Figure 2:**
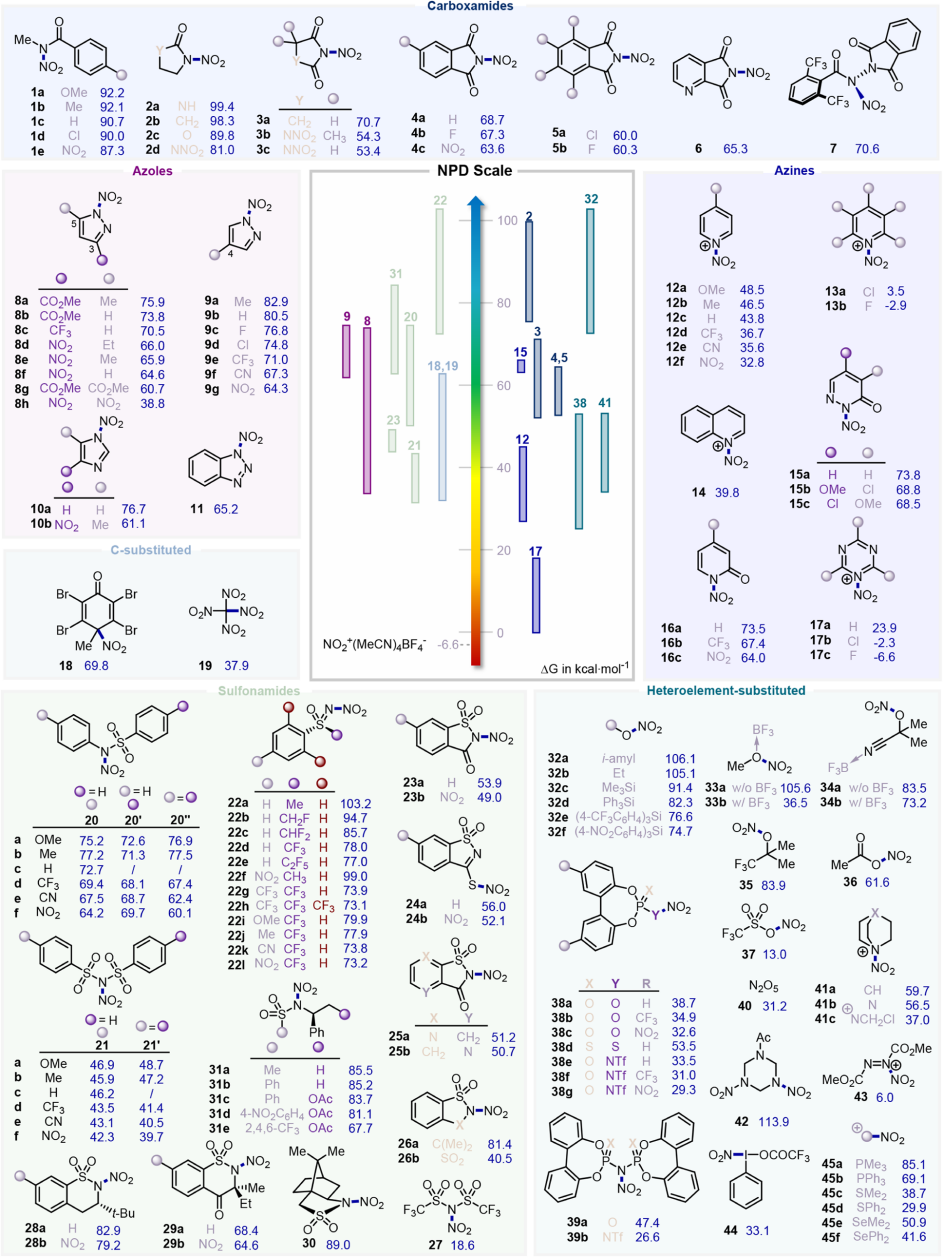
Computed nitro plus detachment (NPD) values for various nitrating reagents (see the SI for further details). Values are reported in kcal·mol^–1^.

The NRA scale was defined as the energy of the LUMO orbital in the nitrating reagent (Figure 1C, right). It is well established that this energy serves as an excellent descriptor for reduction potentials, which are the key parameters governing SET reduction of the nitrating reagents.^46,47^ Accordingly, lower LUMO energies correspond to lower reduction potentials, indicating reagents that are more readily reduced. On this scale, NO_2_^+^BF_4_^–^ exhibits a LUMO energy of −2.094 eV. Unless otherwise stated, all NRA values shown in Figure 3 are reported in eV.

**Figure 3:**
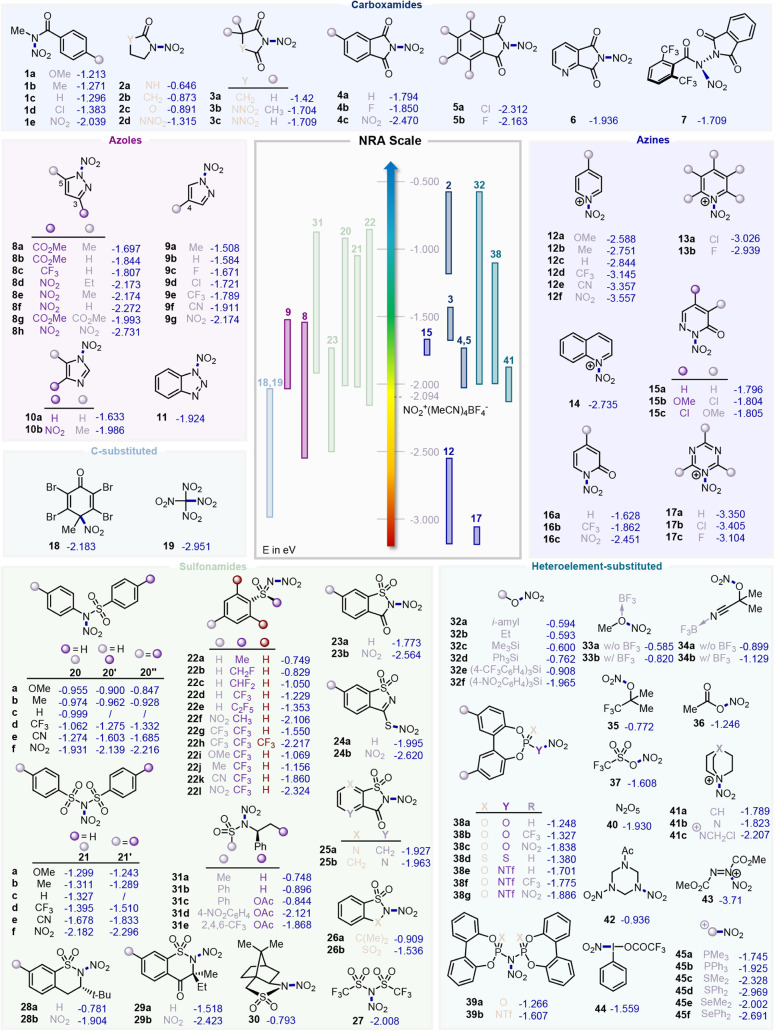
Computed nitryl radical activation (NRA) values for various nitrating reagents (see SI for further details). Values are reported in kcal·mol^–1^.

## Results and Discussion

### Validation against Experimental Data

While no experimental value of NPD (Δ_r_G) has been reported, the enthalpy change (Δ_r_H) associated with the heterolytic N–NO_2_ bond cleavage in several *N*-nitro arylcarboxamides **1a**–**e** has been experimentally determined in acetonitrile.^29^ We therefore computed the corresponding Δ_r_H values for these reagents and compared them to the experimental data. Pleasingly, the DFT approach reproduced the experimental Δ_r_H values with excellent fidelity (Figure 4).

**Figure 4:**
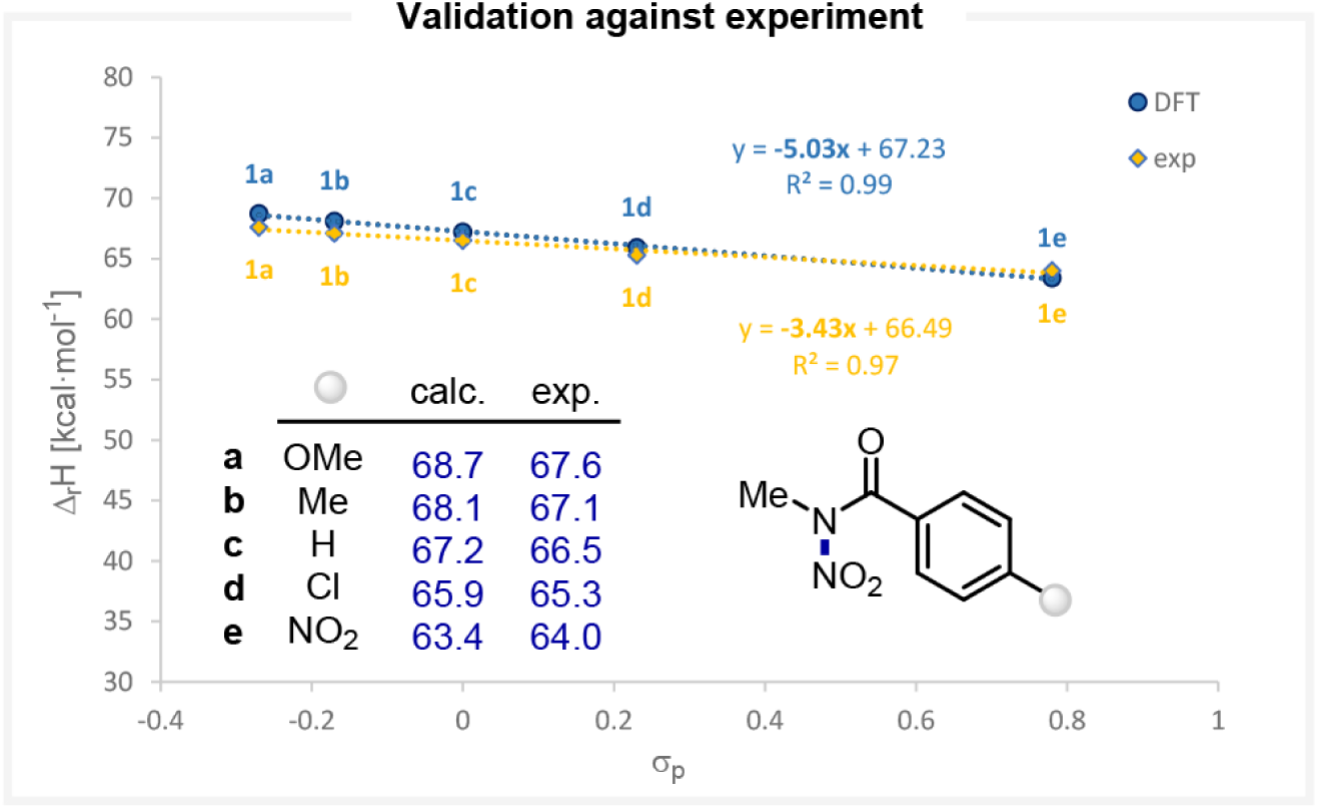
Comparison with experimental data (from ref. 29) and correlation between the computed Δ_r_H values for N–N bond heterolytic dissociation and the electronic nature of the substituents at *N*-nitro arylcarboxamides.

Notably, we found that an appropriate treatment of the nitronium ion reference was critical to achieving a quantitative agreement. In particular, the inclusion of explicit acetonitrile solvent molecules to better describe the solvation environment of the nitronium ion in solution was required to obtain accurate values.

The introduced NPD scale was originally established and experimentally validated in acetonitrile; however, other solvents have also been shown to be effective media for nitration reactions.^9,15,48–50^ While acetonitrile was chosen here as a representative polar, weakly coordinating medium commonly employed in modern nitration chemistry, the qualitative trends described by the NPD scale are expected to remain intact across different solvents, albeit with systematic shifts in absolute values. For example, NPD values are anticipated to decrease in more polar and/or strongly coordinating solvents (e.g., hexafluoroisopropanol, 1,4-dioxane) and to increase in less polar, weakly coordinating solvent systems (e.g., dichloroethane).

### *N*-Nitro Carboxamides and Carboximides

Acyclic *N*-nitro arylcarboxamides^29^ exhibit a relatively high NPD value (∼90 kcal·mol^–1^), consistent with the presence of a strong N–N bond in these reagents. The influence of the substituent electronic effects on the aryl motif was evaluated by correlating the NPD values with the Hammett σ parameters of the substituents (Figure 5). A strong linear correlation was observed (*r* ^2^ = 0.99), with a slope of −4.8, indicating a pronounced sensitivity of the NPD to electronic perturbation within this scaffold.

**Figure 5:**
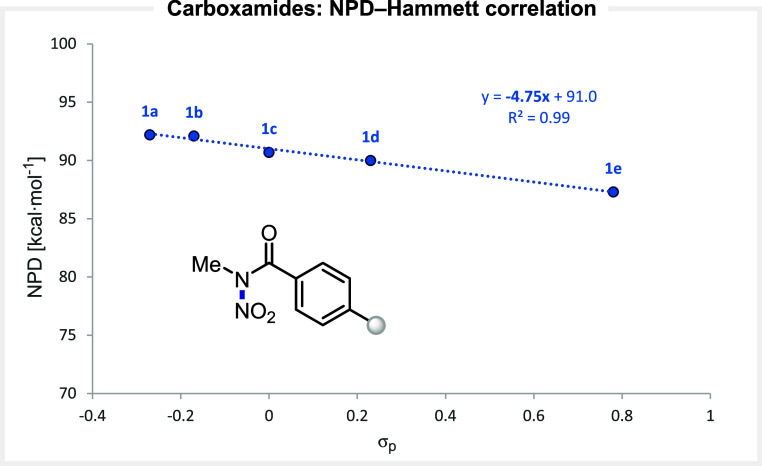
Correlation between the computed NPD values and the electronic nature of the substituents at *N*-nitro arylcarboxamides.

The *N*-nitration of various cyclic carboxamides and carboximide derivatives has been previously investigated, and several of these reagents have been employed as electrophilic nitrating reagents.^15,51^ *N*-nitrated cyclic urea **2a** ^52^ and pyrrolidinone **2b** ^18,53^ exhibit comparable NPD values of 99.4 and 98.3, respectively. The introduction of a more strongly electron-withdrawing oxygen atom in cyclic carbamate **2c** ^15^ significantly lowers the NPD to 89.8. Nitration at the second nitrogen site of **2a** affords the dinitrated derivative **2d**,^51^ which displays an even lower NPD value of 81.0.

A pronounced decrease in NPD is observed upon moving to carboximide derivatives. Succinimide^17,18^ **3a** exhibits a markedly lower value of 70.7, while further nitration of both nitrogen sites in hydantoin derivatives (**3b** and **3c**)^54,55^ reduces the NPD to 54.3 and 53.4, respectively. Incorporation of an aromatic ring, as in phthalimide **4a**,^17^ results in a slightly lower NPD of 68.7 compared to **3a** (70.7). Substitution on the aromatic ring with electron-withdrawing groups, such as fluorine (**4b**) or nitro (**4c**) further decreases the NPD to 67.3 and 63.6, respectively.

Perhalogenated phthalimides **5a** and **5b**, inspired by redox-active ester frameworks,^56^ display notably lower NPD values of 60.0 and 60.3. Replacing the benzenoid ring with a pyridyl unit (**6**) introduces an electron-withdrawing effect, which reduces the NPD to 65.3. Finally, the anomeric amide **7**, recently employed in Markovnikov hydronitration of alkenes by the Baran group,^57^ exhibits an intermediate NPD value of 70.6.

### *N*-Nitro Azoles

A series of *N*-nitropyrazoles has been synthesized and employed in electrophilic aromatic nitration by Zhuang, Zhou, and Shi groups.^15^ The NPD values of these reagents, together with those of newly designed *in silico* derivatives, were computed and found to range from 82.9 for the 4-methyl substituted pyrazole **9a** to 38.8 for the 3,5-dinitro substituted derivative **8h**.

To elucidate how substituent electronics influence the heterolytic cleavage of the N–NO_2_ bond in these pyrazoles, we plotted the NPD values as a function of the sum of the Hammett σ parameters for substituents at the 3-, 4-, and 5-positions (Figure 6). A strong linear correlation was obtained (*r* ^2^ = 0.94), confirming that the electronic effects of substituents directly govern the NPD. Notably, the steep slope of −26.1 reveals a substantially enhanced electronic influence on the heterolytic N–NO_2_ bond cleavage in this system. This computational trend mirrors experimental observations reported by the same authors, who found that electron-donating substituents afford lower nitration yields, whereas electron-withdrawing substituents lead to higher yields. Plotting the reported experimental yields for 4-substituted reagents against the corresponding Hammett parameters further confirmed the excellent correlation between NPD values and the nitrating power of these reagents. Such cross-validation between experiment and computation underscores the predictive value of this DFT-derived NPD scale for rationalizing and predicting the reactivity of nitrating reagents.

**Figure 6:**
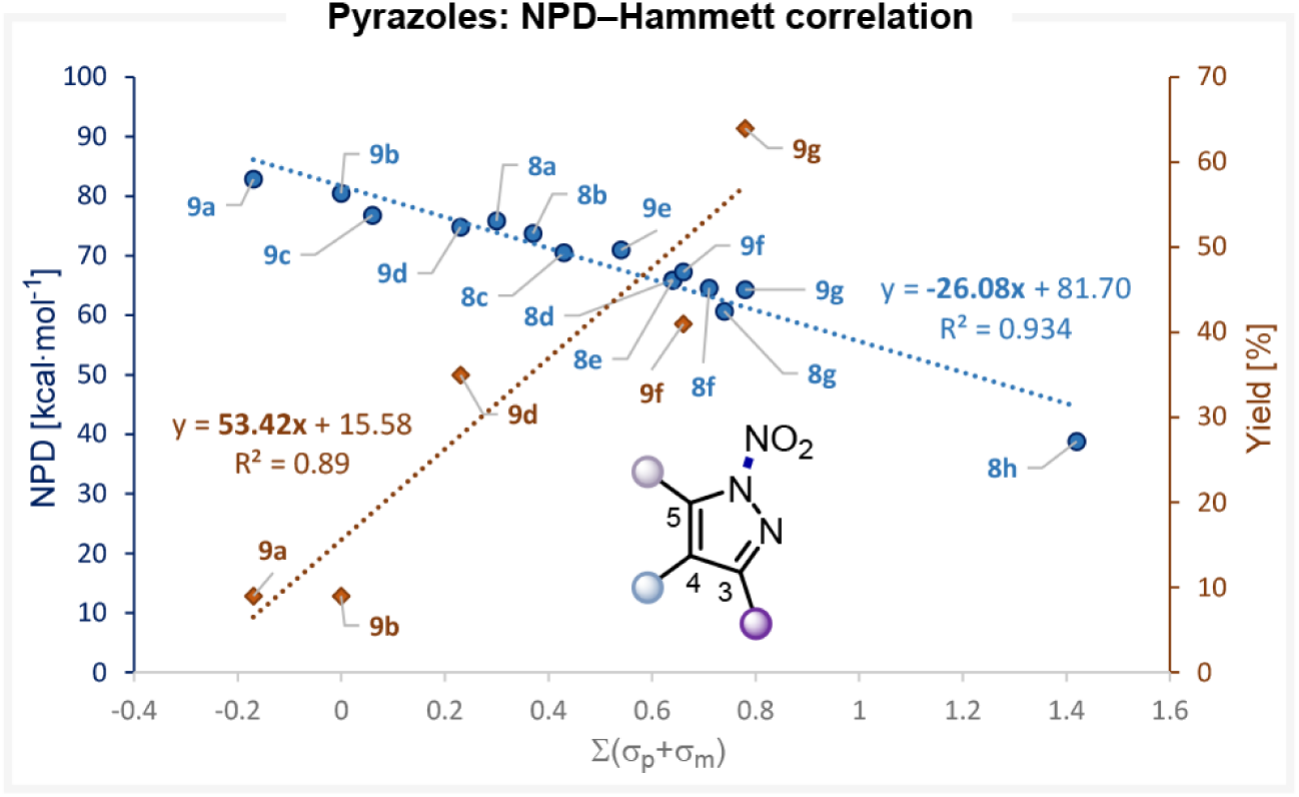
Correlation between the computed NPD values and the sum of the Hammett parameters of the substituents at *N*-nitro pyrazoles (blue), and correlation between the experimental yield of 4-substituted *N*-nitro pyrazoles (extracted from ref. 15) and the Hammett parameters (gold).

Notably, unsubstituted imidazole **10a** ^58^ exhibits an NPD of 76.7, approximately 4 kcal·mol^–1^ lower than pyrazole **9b** (80.5). A similar difference is observed between substituted imidazoles **10b** (61.1) and **8e** (71.0). In comparison, unsubstituted benzotriazole **11** ^15,59^ shows a markedly reduced NPD of 65.2—about 15 kcal·mol^–1^ lower than that of unsubstituted pyrazole **9b**.

### N-Nitro Azines

*N*-nitro pyridinium salts,^1,60^ extensively studied by the group of Olah, have demonstrated high reactivity, but their synthetic utility is limited by their low stability. Computed NPD values for a series of substituted pyridinium derivatives are accordingly quite low, ranging from 48.5 for the 4-methoxy-substituted reagent **12a** to 32.8 for the 4-nitro-substituted reagent **12f**. An excellent correlation between the NPD and substituent’s Hammett parameters is obtained, confirming the linear dependence between the strength of the N–NO_2_ bond and the electronic nature of the substituents (Figure 7).

**Figure 7:**
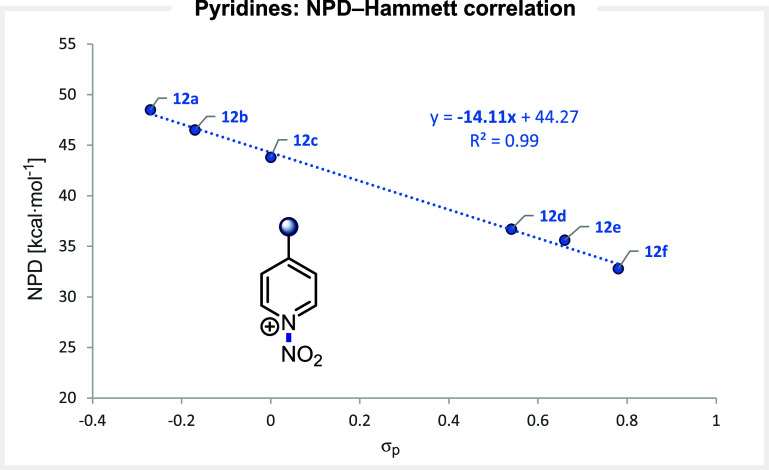
Correlation between the computed NPD values and the electronic nature of the substituents at *N*-nitro pyridines.

The perfluorinated reagent **13b,** derived from a pyridine, which can only be protonated by superacids,^61^ and its perchlorinated analog **13a**, exhibit extremely low NPD values of −2.9 and 3.5, respectively. This suggests that reagent **13a** is thermodynamically unstable, as the NO_2_-complex is less stable than the free pyridine and nitronium ion in acetonitrile.

The NPD value calculated for quinoline **14** is 39.8, which is about 4 kcal·mol^– 1^ lower than that for pyridine **12c** as a result of the extended aromatic conjugation. Pyridazinone **15a**–**c** ^62^ and pyridinone **16a**–**c** exhibit NPD values ranging from 74 to 64. Finally, triazine **17a** has a low NPD value of 23.9, consistent with its weak basicity, while chlorinated and fluorinated analogs show negative NPD values of −2.3 and −6.6, respectively. These negative values inform us that, while *N*-fluoro trichlorotriazine is known to be a powerful fluorinating reagent,^63^ the analogous nitrating reagent is thermodynamically unstable.

### *N*-Nitro Sulfonyl-Based Reagents

*N*-Arylsulfonamides have proven to be highly versatile scaffolds for the design of electrophilic reagents, including fluorinating agents.^64^ This framework allows extensive electronic tuning by varying substituents on both the aniline and arylsulfonyl moieties. To understand how these modifications influence the heterolytic cleavage of the N–NO_2_ bond, we computed the NPD values for a series of electronically varied *N*-nitro *N*-arylsulfonamides^65,66^ (ranging from 77.5 for **20b″** to 60.1 for **20f″**). A linear correlation was observed between the NPD and the corresponding Hammett σ parameters for substituents on either the aniline or the arylsulfonyl rings (Figure 8). Notably, the slope of the correlation was substantially steeper for the aniline series (−10.3) compared to the arylsulfonyl series (−3.7), indicating that electronic perturbations at the aniline core exert approximately three times greater influence on the heterolytic bond dissociation than those at the sulfonyl core.

**Figure 8:**
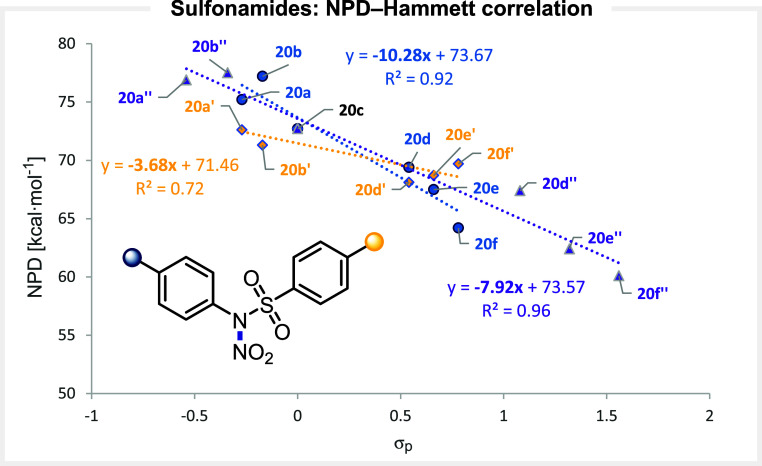
Correlation between the computed NPD values and the electronic nature of the substituents at *N*-nitro arylsulfonamides.

Substituting the aniline’s aromatic core with a second arylsulfonyl motif leads to a series of *N*-nitro sulfonimides^67^ **21** and **21’** — reminiscent of the well-known NFSI reagent — that exhibit significantly lower NPD values, with 46.2 for **21c** compared to 72.7 for **the 20c** analogue. Variation of the substituent at only one aromatic core (**21a**–**f**) leads to a linear correlation for NPD versus Hammett constant parameters, with a small slope coefficient of −4.1 (Figure 9).

**Figure 9:**
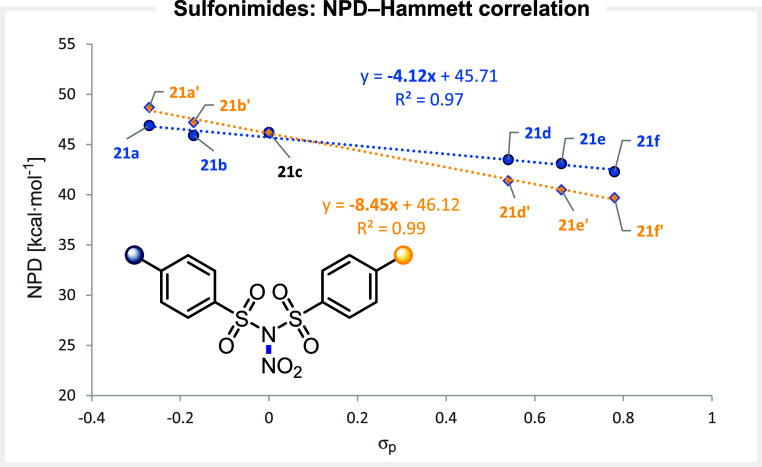
Correlation between the computed NPD values and the electronic nature of the substituents at *N*-nitro sulfonimides.

Variation of substituents at both aromatic cores yields a linear correlation with a doubled slope coefficient of −8.5, suggesting the additivity of the electronic effects. Overall, NPD values range from 48.7 for **21a’** bearing methoxy substituents, to 39.7 for **21f’** bearing two nitro substituents.

Nitration of arylsulfoximines to access *N*-nitro arylsulfoximines has been known for many decades,^68,69^ yet the use of these latter as nitrating reagents has never been explored. The NPD values of a series of *N*-nitro arylsulfoximines were thus systematically computed and spanned a 30 kcal·mol^– 1^ window, from 103.2 for **22a** to 73.2 for **22l**. Interestingly, the substituent at sulfur has a massive impact on the NPD value, with 103.2 for a CH_3_ substituent (**22a**) and 78.0 for a CF_3_ substituent (**22d**). Using Hammett σ_*p*_ parameters for various fluoroalkyl groups present at the sulfur atom led to an excellent correlation (*r* ^2^ = 0.99) between the NPD value of the reagent and the electronic properties of the substituents (Figure 10). The slope value of −37.5 associated with this straight line demonstrates the strong influence of this substituent on the NPD value, most likely due to its proximity to the N–NO_2_ bond. Substituent variation at the aromatic core also afforded a linear correlation, but the slope coefficient of −6.1 obtained is of much smaller magnitude, suggesting a more modest effect in this case.

**Figure 10:**
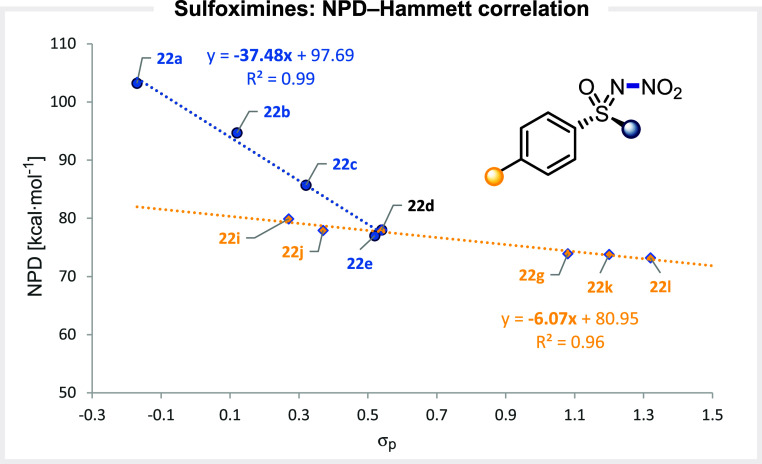
Correlation between the computed NPD values and the electronic nature of the substituents at *N*-nitro sulfoximines.

Besides these readily tunable classes of compounds, the NPD values of other important nitrating reagents have been computed. *N*-Nitrosaccharin reagent^9,16,70^ **23a** has an NPD of 53.9, and the more reactive *N*,6-dinitrosaccharin^27^ **23b** exhibits a value of 49.0. The thiosaccharin analogs **24a** and **24b** were found to exhibit similar NPD values of 56.0 and 52.1, respectively, although the NO_2_ was found to be more stable at sulfur than at the nitrogen atom in this case. Substitution of the benzenoid core of saccharin by a pyridyl motif decreases the NPD to 51.2 for **25a** and 50.7 for **25b**. Structurally analogous to saccharin **23a**, sultam **26a** ^71^ exhibits an NPD of 81.4, showcasing the impact associated with substituting the carbonyl with a *gem*-dimethyl fragment. On the other hand, substituting this carbonyl with an SO_2_ fragment leads to cyclic sulfonimide **26b,** which exhibits a low NPD of 40.5, notably lower than acyclic sulfonimide **21c** (40.0). A very low value was obtained in the case of the bistriflimide reagent **27**,^72^ with an NPD of 18.6.

A significant number of chiral sulfonamide-based scaffolds have been successfully employed in asymmetric synthesis, for instance, in the development of various asymmetric fluorination strategies. With this in mind, we computed the NPD of the reagent based on several important chiral scaffolds. Readily accessible chiral phenylglycine-derived arylsulfonamides^73^ **31a**–**e** exhibit an NPD in the range of 85.5 to 81.1. Remarkably, this value can drop to 67.7 upon the installation of a nonafluoromesitylene sulfonyl group (Nms) in **31e**.^74^ Chiral sultams^75^ **28a** and its NO_2_-substituted analog **28b** exhibit relatively high NPD values of 82.9 and 79.2, which can drop to the range of 64–69 for **29a**–**b** ^76^ upon the introduction of a carbonyl motif. Finally, Oppolzer’s chiral auxiliary derived from camphorsultam **30** ^77,78^ exhibits a computed NPD of 89.0.

### Heteroelement-Substituted Reagents

Nitrate esters^79^ have been known as suitable nitrating reagents for more than a century, and these species generally react only with strong nucleophiles^60,80^ unless additionally activated—such as through the action of a Lewis acid. Consistent with this observation, they exhibit very high NPDs of 106.1 for *i*-amyl nitrate **32a** and 105.1 for ethyl nitrate **32b**. Silylated nitrate esters^81,82^ show lower NPDs, with 91.4 obtained for Me_3_SiONO_2_ **32c**. Substituents at silicon significantly alter the NPD value, which decreases to 82.3 for Ph_3_Si derivatives **32d**, and 76.6 and 74.7 for CF_3_ or NO_2_ *para*-substituted Ar_3_Si derivatives **32e** and **32f**, respectively. Lewis acids can further modulate the nitrating power of nitrate esters, as exemplified by methyl nitrate **33a**,^83^ whose NPD decreases from 105.6 to 36.5 upon complexation with BF_3_ (**33b**), and reagent **34a**,^84^ for which the NPD drops from 83.5 to 73.2 upon complexation (**34b**). CF_3_-substituted reagent **35** was found to be a better nitrating reagent than common alkyl nitrates,^85^ and exhibits an NPD of 83.9, significantly lower than that of alkyl nitrates **32a**–**b**.

Acetyl nitrate **36**,^86^ a common, powerful nitrating reagent, exhibits an NPD of 61.6. On the other hand, the highly reactive nitronium triflate **37**,^87^ derived from highly acidic triflic acid, shows an NPD as low as 13.0. Owing to the widespread generalization of chiral phosphoric acids in asymmetric synthesis over the last two decades, the NPD of a series of nitronium phosphates^88,89^ has been computed. Unsubstituted BINOL-based **38a** shows an NPD of 38.7, an intermediate value between that of reagents **36** and **37**, suggesting a high nitrating power for such compounds. The introduction of CF_3_ or NO_2_ electron-withdrawing substituents further decreases the NPD to 34.9 for **38b** and 32.6 for **38c**, respectively. While nitronium dithiophosphate **38d** exhibits an NPD of 53.5, significantly higher than that of the **38a** analog, nitronium phosphoramidate shows lower NPD values, ranging from 33.5 for **38e** to 31.0 for **38g**. In the series of diphosphate derivatives,^90^ nitronium imidodiphosphate (IDP) **39a** has a computed NPD of 47.4, while the imidodiphosphorimidates (IDPi) derivative **39b** exhibits a lower value of 26.6. These reagents are expected to have a nitrating ability similar to dinitrogen pentoxide **40**,^91^ which has a computed NPD of 31.2.

*N*-nitroammonium salt derived from quinuclidine (**41a**) and DABCO (**41b**) have NPD values of 59.7 and 56.5, respectively, showcasing the influence of the introduction of a nitrogen atom. Inspired by the highly powerful fluorinating reagent Selectfluor, nitrating reagent **41c** was investigated and exhibits a significantly lower NPD of 37.0, demonstrating the charge-enhanced electrophilicity in this scaffold. As opposed to these ammonium salts, triazine^55^ **42** possesses a very high NPD of 113.9. Of note, the nitronium salt of diethyl azodicarboxylate (DEAD) **43** is computed with an extremely low NPD value of 6.0.

Heavy heteroelement-based nitrating reagents have been designed. For instance, *in situ*-produced hypervalent iodine **44** ^92^ has a low computed NPD of 33.1. The Olah group has investigated the use of several nitronium salts **45** in electrophilic aromatic nitration reactions. Among these, nitro phosphonium salts **45a**–**b** have computed NPD values in the range of 85.1–69.1, nitro sulfonium **45c**–**d** exhibits considerably lower values in the range of 38.7–29.9, and selenonium derivatives **45e**–**f** show intermediate values in the range 50.9–41.6.

### *C*-Substituted Nitrating Reagents

A handful of carbon-based reagents have been reported in the context of nitration chemistry. Nitrocyclohexadienone **18**,^93,94^ with an NPD of 69.8, has demonstrated interesting nitrating ability in the context of the nitration of naphthols. Tetranitromethane **19** ^95,96^ is also a powerful nitrating reagent employed in various contexts and exhibits an NPD value of 37.9.

## Conclusions

In summary, we conducted a comprehensive computational investigation of more than 150 nitrating reagents to rationalize and quantify their electrophilic nitrating abilities. The resulting NPD scale provides a unified thermodynamic metric that accurately reflects the heterolytic Y–NO_2_ bond dissociation tendency across diverse structural classes, including *N*-nitro carboxamides and carboximides, azoles, azines, sulfonamides and sulfonimides, sulfoximines, and heteroatom- and carbon-based frameworks. The strong linear correlations observed between NPD values, Hammett parameters, and available experimental data establish the predictive power of this scale for guiding future reagent design.

The analysis reveals clear trends in structure–reactivity relationships: 1) electron-withdrawing substituents consistently lower NPD values, enhancing nitrating ability; 2) the magnitude of the substituent effects depends strongly on the underlying scaffold and increases in the order Ar-sulfonyl < Ar-carboxamide < Ar-sulfoximine < Ar-aniline < pyridine < pyrazole < *S*-atom sulfoximine; and 3) Lewis acid activation or cationic groups can markedly increase the nitrating ability of reagents. Together, these findings provide a coherent framework for rationalizing the reactivity of known nitrating reagents and predicting the behavior of unreported ones.

A complementary NRA scale was presented to provide insight into the ease of reducing these reagents through SET reductive strategies. These frameworks have recently emerged as convenient and powerful methods for introducing the nitro group into aromatics and olefins via the intermediacy of nitryl radical species.

Beyond their synthetic relevance, the scales introduced herein represent foundational tools for guiding both electrophilic and radical nitration chemistry. We anticipate that this work will contribute to systematic reagent optimization and accelerate fine-tuning in reagent design.

## Supplementary Material



## Data Availability

The data underlying this study are available in the published article and its Supporting Information.

## Notes

Computational details, calculated energies, and Cartesian geometries of optimized structures (PDF)
