# Supplementary material for: Electrophilic and Radical Ability of Organic Nitrating Reagents
Source: J Org Chem. 2026 Feb 18;91(8):3138–49. doi: 10.1021/acs.joc.5c02809 (PMC12954762; doi:10.1021/acs.joc.5c02809)
Supplement: Supplementary file 1 [file jo5c02809_si_001.pdf]

Supporting Information

Electrophilic and Radical Ability of Organic Nitrating Reagents

Anthony J. Fernandes, Harry Lecomte, Dmitry Katayev*

Department of Chemistry, Biochemistry and Pharmaceutical Sciences, University of Bern, Freiestrasse 3, 3012 Bern, Switzerland

*Corresponding authors

E-mail: dmitry.katayev@unibe.ch

Table of content

1. Computed energies -- S3

2. Computed structures -- S17

1. Computed energies

Table S1. Computed energies and frontier molecular orbitals energies in Hartree unless otherwise stated. Computed at the M06-2X-D3/[6-311++G(2df,2p)+def2-QZVPPD(Se,Br,I)],SMD(MeCN) // M06-2X-D3/6-311++G(2d,p),SMD(MeCN) level of theory. *Geometry of reagent **44** obtained using M06-2X-D3/def2-TZVP,SMD(MeCN).

N°	Thermal correction		SP energy	*ΔH*	*ΔG*	BDE	BDFE	HOMO (eV)	LUMO (eV)
	to *H*	to *G*				*ΔH in kcal·mol^{-1}*	*ΔG in kcal·mol^{-1}*		
**1a**	0.206413	0.150001	-759.230857	-759.024444	-759.080856	68.7	92.2	-7.880	-1.213
**1a-anion**	0.187813	0.138539	-554.260506	-554.072693	-554.121967				
**1b**	0.200005	0.14447	-684.0166609	-683.8166559	-683.8721909	68.1	92.1	-8.347	-1.271
**1b-anion**	0.18153	0.133866	-479.0473848	-478.8658548	-478.9135188				
**1c**	0.170845	0.119943	-644.70585	-644.535005	-644.585907	67.2	90.7	-8.684	-1.296
**1c-anion**	0.152343	0.108565	-439.737916	-439.585573	-439.629351				
**1d**	0.162388	0.107508	-1104.311148	-1104.14876	-1104.20364	65.9	90.0	-8.502	-1.383
**1d-anion**	0.143933	0.097113	-899.3453794	-899.2014464	-899.2482664				
**1e**	0.17621	0.118023	-849.2105842	-849.0343742	-849.0925612	63.4	87.3	-9.157	-2.039
**1e-anion**	0.157818	0.107446	-644.2488964	-644.0910784	-644.1414504				
**2a**	0.111538	0.070444	-507.1555365	-507.0439985	-507.0850925	75.9	99.4	-9.037	-0.646
**2a-anion**	0.092813	0.058976	-302.1735539	-302.0807409	-302.1145779				
**2b**	0.122673	0.081104	-491.0959188	-490.9732458	-491.0148148	74.8	98.3	-9.514	-0.873
**2b-anion**	0.103788	0.06943	-286.1155603	-286.0117723	-286.0461303				
**2c**	0.099173	0.05826	-527.0140707	-526.9148977	-526.9558107	66.4	89.8	-10.086	-0.891
**2c-anion**	0.080103	0.046126	-322.0468634	-321.9667604	-322.0007374				
**2d**	0.116752	0.069201	-711.6204902	-711.5037382	-711.5512892	57.5	81.0	-10.002	-1.315
**2d-anion**	0.09755	0.057192	-506.6673675	-506.5698175	-506.6101755				

N°	Thermal correction		SP energy	*ΔH*	*ΔG*	BDE *ΔH in kcal·mol^-1^*	BDFE *ΔG in kcal·mol^-1^*	HOMO (eV)	LUMO (eV)
	to *H*	to *G*							
**3a**	0.103603	0.059953	-565.1236001	-565.0199971	-565.0636471	47.1	70.7	-9.822	-1.42
**3a-anion**	0.085748	0.049436	-360.188322	-360.102574	-360.138886				
**3b**	0.15582	0.099976	-864.2599175	-864.1040975	-864.1599415	30.8	54.3	-10.245	-1.704
**3b-anion**	0.137691	0.089059	-659.3503999	-659.2127089	-659.2613409				
**3c**	0.097385	0.047608	-785.6388605	-785.5414755	-785.5912525	30.1	53.4	-10.448	-1.709
**3c-anion**	0.07945	0.036615	-580.7306995	-580.6512495	-580.6940845				
**4a**	0.130085	0.081504	-717.5374483	-717.4073633	-717.4559443	44.9	68.7	-9.185	-1.794
**4a-anion**	0.112372	0.071382	-512.6058792	-512.4935072	-512.5344972				
**4b**	0.122507	0.07182	-816.7817515	-816.6592445	-816.7099315	43.5	67.3	-9.072	-1.85
**4b-anion**	0.104783	0.061703	-611.8524263	-611.7476433	-611.7907233				
**4c**	0.135237	0.080014	-922.0369544	-921.9017174	-921.9569404	39.9	63.6	-9.636	-2.47
**4c-anion**	0.117546	0.069879	-717.1133874	-716.9958414	-717.0435084				
**5a**	0.096496	0.035395	-2555.927748	-2555.831252	-2555.892353	36.7	60.0	-8.942	-2.312
**5a-anion**	0.078683	0.024413	-2351.009116	-2350.930433	-2350.984703				
**5b**	0.101632	0.044843	-1114.480911	-1114.379279	-1114.436068	36.6	60.3	-9.31	-2.163
**5b-anion**	0.083682	0.03425	-909.5622659	-909.4785839	-909.5280159				
**6**	0.118215	0.069947	-733.5716222	-733.4534072	-733.5016752	41.5	65.3	-9.439	-1.936
**6-anion**	0.100573	0.059856	-528.6454632	-528.5448902	-528.5856072				
**7**	0.262152	0.17833	-1791.37367	-1791.111518	-1791.19534	46.7	70.6	-9.083	-1.709
**7-anion**	0.244402	0.168461	-1586.439232	-1586.19483	-1586.270771				
**8a**	0.157641	0.104618	-697.8481638	-697.6905228	-697.7435458	51.3	75.9	-8.997	-1.697
**8a-anion**	0.138363	0.094329	-492.9048807	-492.7665177	-492.8105517				
**8b**	0.128365	0.07886	-658.5350061	-658.4066411	-658.4561461	49.9	73.8	-9.434	-1.844

N°	Thermal correction		SP energy	*ΔH*	*ΔG*	BDE	BDFE	HOMO (eV)	LUMO (eV)
	to *H*	to *G*				*ΔH in kcal·mol^{-1}*	*ΔG in kcal·mol^{-1}*		
**8b-anion**	0.110146	0.068487	-453.5949695	-453.4848235	-453.5264825				
**8c**	0.089079	0.042116	-767.7223765	-767.6332975	-767.6802605	47.6	70.5	-9.569	-1.807
**8c-anion**	0.070871	0.030144	-562.7859913	-562.7151203	-562.7558473				
**8d**	0.145531	0.095319	-713.7762088	-713.6306778	-713.6808898	43.4	66.0	-9.304	-2.173
**8d-anion**	0.127055	0.082656	-508.8462673	-508.7192123	-508.7636113				
**8e**	0.115813	0.068927	-674.4696141	-674.3538011	-674.4006871	43.2	65.9	-9.314	-2.174
**8e-anion**	0.097233	0.056146	-469.5398453	-469.4426123	-469.4836993				
**8f**	0.086156	0.042496	-635.1555336	-635.0693776	-635.1130376	41.3	64.6	-9.776	-2.272
**8f-anion**	0.067947	0.031005	-430.2291025	-430.1611555	-430.1980975				
**8g**	0.176195	0.116632	-886.4000689	-886.2238739	-886.2834369	37.1	60.7	-9.595	-1.993
**8g-anion**	0.15805	0.105751	-681.4804737	-681.3224237	-681.3747227				
**8h**	0.091223	0.040371	-839.6267431	-839.5355201	-839.5863721	15.3	38.8	-10.455	-2.731
**8h-anion**	0.073441	0.029763	-634.7422229	-634.6687819	-634.7124599				
**9a**	0.110211	0.068658	-469.9704651	-469.8602541	-469.9018071	60.1	82.9	-8.757	-1.508
**9a-anion**	0.091679	0.056229	-265.0138331	-264.9221541	-264.9576041				
**9b**	0.080616	0.043211	-430.6597407	-430.5791247	-430.6165297	57.2	80.5	-9.21	-1.584
**9b-anion**	0.062321	0.031759	-225.7079489	-225.6456279	-225.6761899				
**9c**	0.073417	0.033882	-529.8917661	-529.8183491	-529.8578841	53.6	76.8	-9.098	-1.671
**9c-anion**	0.055263	0.022466	-324.9458672	-324.8906042	-324.9234012				
**9d**	0.072377	0.031422	-890.256454	-890.184077	-890.225032	51.6	74.8	-8.873	-1.721
**9d-anion**	0.054349	0.020141	-685.3139057	-685.2595567	-685.2937647				
**9e**	0.089259	0.041987	-767.7236923	-767.6344333	-767.6817053	48.0	71.0	-9.732	-1.789
**9e-anion**	0.070987	0.03009	-562.7866656	-562.7156786	-562.7565756				

N°	Thermal correction		SP energy	*ΔH*	*ΔG*	BDE *ΔH in kcal·mol^{-1}*	BDFE *ΔG in kcal·mol^{-1}*	HOMO (eV)	LUMO (eV)
	to *H*	to *G*							
**9f**	0.081202	0.039242	-522.9024502	-522.8212482	-522.8632082	44.0	67.3	-9.455	-1.911
**9f-anion**	0.063211	0.02812	-317.9720847	-317.9088737	-317.9439647				
**9g**	0.086122	0.041951	-635.1550244	-635.0689024	-635.1130734	40.9	64.3	-9.941	-2.174
**9g-anion**	0.068022	0.030828	-430.2293602	-430.1613382	-430.1985322				
**10a**	0.081012	0.043563	-430.6812854	-430.6002734	-430.6377224	53.4	76.7	-8.671	-1.633
**10a-anion**	0.062918	0.032422	-225.7358026	-225.6728846	-225.7033806				
**10b**	0.115986	0.068524	-674.4944467	-674.3784607	-674.4259227	37.7	61.1	-8.972	-1.986
**10b-anion**	0.097702	0.05728	-469.5737391	-469.4760371	-469.5164591				
**11**	0.118118	0.074585	-600.3097168	-600.1915988	-600.2351318	41.7	65.2	-8.63	-1.924
**11-anion**	0.100263	0.063798	-395.3830098	-395.2827468	-395.3192118				
**12a**	0.148301	0.103708	-567.6929235	-567.5446225	-567.5892155	25.6	48.5	-9.824	-2.588
**12a-anion**	0.130254	0.091973	-362.7918136	-362.6615596	-362.6998406				
**12b**	0.141517	0.097782	-492.4742995	-492.3327825	-492.3765175	23.3	46.5	-10.236	-2.751
**12b-anion**	0.123696	0.086605	-287.5770242	-287.4533282	-287.4904192				
**12c**	0.112466	0.073042	-453.1581931	-453.0457271	-453.0851511	20.5	43.8	-10.276	-2.844
**12c-anion**	0.094528	0.061953	-248.2653303	-248.1708023	-248.2033773				
**12d**	0.120757	0.072029	-790.2141064	-790.0933494	-790.1420774	13.4	36.7	-10.568	-3.145
**12d-anion**	0.102917	0.060941	-585.3325405	-585.2296235	-585.2715995				
**12e**	0.112418	0.068387	-545.3889824	-545.2765644	-545.3205954	12.2	35.6	-10.621	-3.357
**12e-anion**	0.094837	0.057763	-340.5096417	-340.4148047	-340.4518787				
**12f**	0.117307	0.070958	-657.6427234	-657.5254164	-657.5717654	9.5	32.8	-10.711	-3.557
**12f-anion**	0.099725	0.060141	-452.7676099	-452.6678849	-452.7074689				
**13a**	0.069275	0.013684	-2751.099647	-2751.030372	-2751.085963	-19.7	3.5	-9.806	-3.026

N°	Thermal correction		SP energy	*ΔH*	*ΔG*	BDE *ΔH in kcal·mol⁻¹*	BDFE *ΔG in kcal·mol⁻¹*	HOMO (eV)	LUMO (eV)
	to *H*	to *G*							
**13a-anion**	0.052159	0.00326	-2546.271533	-2546.219374	-2546.268273				
**13b**	0.075718	0.025466	-949.289266	-949.213548	-949.2638	-26.6	-2.9	-10.323	-2.939
**13b-anion**	0.058617	0.015763	-744.4721776	-744.4135606	-744.4564146				
**14**	0.161791	0.116527	-606.785598	-606.623807	-606.669071	16.6	39.8	-8.963	-2.735
**14-anion**	0.144028	0.10538	-401.8990604	-401.7550324	-401.7936804				
**15a**	0.092375	0.050591	-543.9811838	-543.8888088	-543.9305928	50.2	73.8	-8.86	-1.796
**15a-anion**	0.074192	0.039596	-339.0406071	-338.9664151	-339.0010111				
**15b**	0.120089	0.06944	-1118.103978	-1117.983889	-1118.034538	45.4	68.8	-8.398	-1.804
**15b-anion**	0.101831	0.05813	-913.1710456	-913.0692146	-913.1129156				
**15c**	0.11968	0.068504	-1118.097372	-1117.977692	-1118.028868	45.0	68.5	-8.396	-1.805
**15c-anion**	0.101552	0.057563	-913.165258	-913.063706	-913.107695				
**16a**	0.104368	0.062628	-527.967053	-527.862685	-527.904425	50.1	73.5	-8.052	-1.628
**16a-anion**	0.086282	0.05157	-323.0268236	-322.9405416	-322.9752536				
**16b**	0.1128	0.061915	-865.0314078	-864.9186078	-864.9694928	44.2	67.4	-8.372	-1.862
**16b-anion**	0.094784	0.050548	-660.1006016	-660.0058176	-660.0500536				
**16c**	0.109543	0.060897	-732.463473	-732.35393	-732.402576	40.7	64.0	-8.493	-2.451
**16c-anion**	0.091588	0.049707	-527.5383154	-527.4467274	-527.4886084				
**17a**	0.08817	0.049	-485.222966	-485.134796	-485.173966	0.6	23.9	-10.949	-3.35
**17a-anion**	0.071169	0.038848	-280.3627061	-280.2915371	-280.3238581				
**17b**	0.061488	0.011892	-1863.98902	-1863.927532	-1863.977128	-26	-2.3	-11.732	-3.405
**17b-anion**	0.04466	0.002498	-1659.171224	-1659.126564	-1659.168726				
**17c**	0.066199	0.020793	-782.9384377	-782.8722387	-782.9176447	-30.3	-6.6	-12.303	-3.104
**17c-anion**	0.049401	0.011502	-578.1276728	-578.0782718	-578.1161708				

N°	Thermal correction		SP energy	*ΔH*	*ΔG*	BDE *ΔH in kcal·mol⁻¹*	BDFE *ΔG in kcal·mol⁻¹*	HOMO (eV)	LUMO (eV)
	to *H*	to *G*							
**18**	0.111299	0.049106	-10845.84775	-10845.73645	-10845.79864	47.6	69.8	-8.883	-2.183
**18-anion**	0.093299	0.036287	-10640.91161	-10640.81831	-10640.87533				
**19**	0.070103	0.018769	-858.4108111	-858.3407081	-858.3920421	14.5	37.9	-11.946	-2.951
**19-anion**	0.052792	0.008318	-653.5280073	-653.4752153	-653.5196893				
**20a**	0.26456	0.197672	-1386.224404	-1385.959844	-1386.026732	52.8	75.2	-7.895	-0.955
**20a-anion**	0.245941	0.184395	-1181.279332	-1181.033391	-1181.094937				
**20b**	0.258044	0.189376	-1311.010965	-1310.752921	-1310.821589	52.7	77.2	-8.382	-0.974
**20b-anion**	0.239628	0.17966	-1106.066236	-1105.826608	-1105.886576				
**20c**	0.228977	0.167918	-1271.700269	-1271.471292	-1271.532351	50.7	72.7	-8.708	-0.999
**20c-anion**	0.210616	0.154413	-1066.758816	-1066.5482	-1066.604403				
**20d**	0.237305	0.167042	-1608.768543	-1608.531238	-1608.601501	46.7	69.4	-8.943	-1.062
**20d-anion**	0.218952	0.154526	-1403.833471	-1403.614519	-1403.678945				
**20e**	0.22922	0.163135	-1363.946346	-1363.717126	-1363.783211	44.4	67.5	-8.903	-1.274
**20e-anion**	0.211491	0.151907	-1159.015545	-1158.804054	-1158.863638				
**20f**	0.234182	0.166372	-1476.20443	-1475.970248	-1476.038058	39.6	64.2	-8.971	-1.931
**20f-anion**	0.214966	0.156126	-1271.279821	-1271.064855	-1271.123695				
**20a'**	0.264785	0.198867	-1386.226184	-1385.961399	-1386.027317	48.9	72.6	-8.143	-0.9
**20a'-anion**	0.244671	0.186141	-1181.285752	-1181.041081	-1181.099611				
**20b'**	0.258298	0.193478	-1311.01173	-1310.753432	-1310.818252	49.1	71.3	-8.581	-0.962
**20b'-anion**	0.239578	0.1799	-1106.072429	-1105.832851	-1105.892529				
**20d'**	0.237565	0.167763	-1608.766924	-1608.529359	-1608.599161	46.3	68.1	-8.769	-1.275
**20d'-anion**	0.218884	0.153566	-1403.832162	-1403.613278	-1403.678596				
**20e'**	0.228314	0.165585	-1363.944268	-1363.715954	-1363.778683	48.1	68.7	-8.779	-1.603

N°	Thermal correction		SP energy	*ΔH*	*ΔG*	BDE *ΔH in kcal·mol⁻¹*	BDFE *ΔG in kcal·mol⁻¹*	HOMO (eV)	LUMO (eV)
	to *H*	to *G*							
**20e'-anion**	0.211139	0.150972	-1159.008116	-1158.796977	-1158.857144				
**20f'**	0.233939	0.165285	-1476.201848	-1475.967909	-1476.036563	47.0	69.7	-8.814	-2.139
**20f'-anion**	0.216137	0.153394	-1271.26685	-1271.050713	-1271.113456				
**20a''**	0.300235	0.228182	-1500.750131	-1500.449896	-1500.521949	54.4	76.9	-7.837	-0.847
**20a''-anion**	0.281726	0.215139	-1295.802577	-1295.520851	-1295.587438				
**20b''**	0.287272	0.217127	-1350.322339	-1350.035067	-1350.105212	52.8	77.5	-8.337	-0.928
**20b''-anion**	0.267822	0.206749	-1145.376369	-1145.108547	-1145.16962				
**20d''**	0.245743	0.166273	-1945.835008	-1945.589265	-1945.668735	44.6	67.4	-9.084	-1.332
**20d''-anion**	0.227242	0.153806	-1740.903123	-1740.675881	-1740.749317				
**20e''**	0.229736	0.159521	-1456.190115	-1455.960379	-1456.030594	39.4	62.4	-9.000	-1.685
**20e''-anion**	0.211701	0.147821	-1251.266987	-1251.055286	-1251.119166				
**20f''**	0.239512	0.16484	-1680.705599	-1680.466087	-1680.540759	37.0	60.1	-9.263	-2.216
**20f''-anion**	0.221323	0.153141	-1475.786064	-1475.564741	-1475.632923				
**21a**	0.276956	0.204548	-1934.821906	-1934.54495	-1934.617358	26.0	46.9	-8.223	-1.299
**21a-anion**	0.260747	0.191356	-1729.922	-1729.661253	-1729.730644				
**21b**	0.270815	0.199589	-1859.6074	-1859.336585	-1859.407811	24.9	45.9	-8.754	-1.311
**21b-anion**	0.25417	0.186167	-1654.708725	-1654.454555	-1654.522558				
**21c**	0.242996	0.174027	-1820.295951	-1820.052955	-1820.121924	23.6	46.2	-8.987	-1.327
**21c-anion**	0.225355	0.162137	-1615.398396	-1615.173041	-1615.236259				
**21d**	0.251302	0.173264	-2157.362223	-2157.110921	-2157.188959	21.3	43.5	-9.007	-1.395
**21d-anion**	0.233325	0.160475	-1952.468	-1952.234675	-1952.307525				
**21e**	0.243236	0.17035	-1912.539463	-1912.296227	-1912.369113	20.8	43.1	-9.014	-1.678
**21e-anion**	0.225606	0.158008	-1707.646398	-1707.420792	-1707.48839				

N°	Thermal correction		SP energy	*ΔH*	*ΔG*	BDE *ΔH in kcal·mol^-1^*	BDFE *ΔG in kcal·mol^-1^*	HOMO (eV)	LUMO (eV)
	to *H*	to *G*							
**21f**	0.248189	0.173046	-2024.796956	-2024.548767	-2024.62391	20.3	42.3	-9.019	-2.182
**21f-anion**	0.230674	0.160386	-1819.90481	-1819.674136	-1819.744424				
**21a'**	0.314026	0.234412	-2049.348149	-2049.034123	-2049.113737	26.5	48.7	-8.135	-1.243
**21a'-anion**	0.296007	0.221459	-1844.445552	-1844.149545	-1844.224093				
**21b'**	0.301121	0.224524	-1898.919026	-1898.617905	-1898.694502	25.0	47.2	-8.677	-1.289
**21b'-anion**	0.283338	0.211773	-1694.019001	-1693.735663	-1693.807228				
**21d'**	0.259734	0.173092	-2494.428295	-2494.168561	-2494.255203	19.1	41.4	-9.297	-1.51
**21d'-anion**	0.241734	0.160325	-2289.537573	-2289.295839	-2289.377248				
**21e'**	0.243976	0.166876	-2004.782983	-2004.539007	-2004.616107	18.0	40.5	-9.341	-1.833
**21e'-anion**	0.22631	0.154758	-1799.894227	-1799.667917	-1799.739469				
**21f'**	0.253616	0.171439	-2229.297692	-2229.044076	-2229.126253	16.9	39.7	-9.455	-2.296
**21f'-anion**	0.236068	0.159915	-2024.410884	-2024.174816	-2024.250969				
**22a**	0.165593	0.113238	-1004.721904	-1004.556311	-1004.608666	79.7	103.2	-8.988	-0.749
**22a-anion**	0.147949	0.102703	-799.7349312	-799.5869822	-799.6322282				
**22b**	0.159631	0.106477	-1103.947864	-1103.788233	-1103.841387	71.9	94.7	-9.07	-0.829
**22b-anion**	0.142097	0.094894	-898.9733582	-898.8312612	-898.8784642				
**22c**	0.152494	0.098147	-1203.191206	-1203.038712	-1203.093059	63.5	85.7	-9.099	-1.05
**22c-anion**	0.135097	0.085779	-998.2302625	-998.0951655	-998.1444835				
**22d**	0.144865	0.089419	-1302.440313	-1302.295448	-1302.350894	56.2	78.0	-9.161	-1.229
**22d-anion**	0.127456	0.076608	-1097.49109	-1097.363634	-1097.414482				
**22e**	0.160118	0.098035	-1540.238708	-1540.07859	-1540.140673	54.7	77.0	-9.168	-1.353
**22e-anion**	0.14305	0.086299	-1335.292164	-1335.149114	-1335.205865				
**22f**	0.170769	0.111689	-1209.223361	-1209.052592	-1209.111672	75.5	99.0	-9.43	-2.106

N°	Thermal correction		SP energy	*ΔH*	*ΔG*	BDE *ΔH in kcal·mol^-1^*	BDFE *ΔG in kcal·mol^-1^*	HOMO (eV)	LUMO (eV)
	to *H*	to *G*							
**22f-anion**	0.153242	0.101317	-1004.2432	-1004.089958	-1004.141883				
**22g**	0.153378	0.089061	-1639.505325	-1639.351947	-1639.416264	53.2	73.9	-9.447	-1.55
**22g-anion**	0.135618	0.073935	-1434.56042	-1434.424802	-1434.486485				
**22h**	0.169945	0.090769	-2313.610643	-2313.440698	-2313.519874	50.5	73.1	-10.133	-2.217
**22h-anion**	0.152212	0.078626	-2108.670039	-2108.517827	-2108.591413				
**22i**	0.180462	0.118581	-1416.966993	-1416.786531	-1416.848412	57.6	79.9	-8.356	-1.069
**22i-anion**	0.162735	0.106215	-1212.015203	-1211.852468	-1211.908988				
**22j**	0.174103	0.114778	-1341.752273	-1341.57817	-1341.637495	56.8	77.9	-8.952	-1.156
**22j-anion**	0.156216	0.100106	-1136.801474	-1136.645258	-1136.701368				
**22k**	0.145407	0.085772	-1394.682419	-1394.537012	-1394.596647	52.4	73.8	-9.521	-1.86
**22k-anion**	0.12773	0.071949	-1189.738897	-1189.611167	-1189.666948				
**22l**	0.150289	0.087974	-1506.939509	-1506.78922	-1506.851535	51.7	73.2	-9.592	-2.324
**22l-anion**	0.132692	0.074398	-1301.997184	-1301.864492	-1301.922786				
**23a**	0.131916	0.079833	-1152.794342	-1152.662426	-1152.714509	29.9	53.9	-9.41	-1.773
**23a-anion**	0.114342	0.070139	-947.8867969	-947.7724549	-947.8166579				
**23b**	0.137099	0.078116	-1357.290881	-1357.153782	-1357.212765	25.0	49.0	-9.862	-2.564
**23b-anion**	0.11956	0.068494	-1152.391203	-1152.271643	-1152.322709				
**24a**	0.129033	0.074796	-1475.753222	-1475.624189	-1475.678426	31.5	56.0	-9.177	-1.995
**24a-anion**	0.112379	0.06688	-1270.844054	-1270.731675	-1270.777174				
**24b**	0.134383	0.073656	-1680.251152	-1680.116769	-1680.177496	27.8	52.1	-9.665	-2.62
**24b-anion**	0.117573	0.065303	-1475.347744	-1475.230171	-1475.282441				
**25a**	0.119903	0.068008	-1168.827935	-1168.708032	-1168.759927	27.3	51.2	-9.741	-1.927
**25a-anion**	0.102457	0.058354	-963.9246341	-963.8221771	-963.8662801				

N°	Thermal correction		SP energy	ΔH	ΔG	BDE	BDFE	HOMO (eV)	LUMO (eV)
	to *H*	to *G*				*ΔH in kcal·mol*$^{-1}$	*ΔG in kcal·mol*$^{-1}$		
**25b**	0.12002	0.068219	-1168.826907	-1168.706887	-1168.758688	26.8	50.7	-9.716	-1.963
**25b-anion**	0.102612	0.058613	-963.924435	-963.821823	-963.865822				
**26a**	0.207616	0.154257	-1157.395044	-1157.187428	-1157.240787	59.6	81.4	-8.891	-0.909
**26a-anion**	0.190132	0.1412	-952.4402761	-952.2501441	-952.2990761				
**26b**	0.133777	0.07954	-1588.044991	-1587.911214	-1587.965451	17.3	40.5	-9.655	-1.536
**26b-anion**	0.116521	0.068912	-1383.157768	-1383.041247	-1383.088856				
**27**	0.08714	0.023613	-2032.285117	-2032.197977	-2032.261504	-4.2	18.6	-11.613	-2.008
**27-anion**	0.070098	0.012572	-1827.432435	-1827.362337	-1827.419863				
**28a**	0.298341	0.236859	-1275.314911	-1275.01657	-1275.078052	60.5	82.9	-8.643	-0.781
**28a-anion**	0.279559	0.223502	-1070.357465	-1070.077906	-1070.133963				
**28b**	0.303569	0.235107	-1479.817467	-1479.513898	-1479.58236	56.3	79.2	-9.139	-1.904
**28b-anion**	0.28519	0.222895	-1274.867044	-1274.581854	-1274.644149				
**29a**	0.250504	0.189037	-1310.016649	-1309.766145	-1309.827612	45.6	68.4	-9.177	-1.518
**29a-anion**	0.232146	0.176618	-1105.083225	-1104.851079	-1104.906607				
**29b**	0.255637	0.186732	-1514.51521	-1514.259573	-1514.328478	41.5	64.6	-9.66	-2.423
**29b-anion**	0.237402	0.174956	-1309.588468	-1309.351066	-1309.413512				
**30**	0.286536	0.22979	-1199.107719	-1198.821183	-1198.877929	65.9	89.0	-9.438	-0.793
**30-anion**	0.267881	0.217658	-994.1417297	-993.8738487	-993.9240717				
**31a**	0.230643	0.173159	-1158.597622	-1158.366979	-1158.424463	64.0	85.5	-8.335	-0.748
**31a-anion**	0.213498	0.160075	-953.6362086	-953.4227106	-953.4761336				
**31b**	0.288496	0.220977	-1350.318051	-1350.029555	-1350.097074	62.3	85.2	-8.328	-0.896
**31b-anion**	0.270374	0.209028	-1145.358285	-1145.087911	-1145.149257				
**31c**	0.336759	0.261063	-1578.203405	-1577.866646	-1577.942342	62.2	83.7	-8.445	-0.844

N°	Thermal correction		SP energy	*ΔH*	*ΔG*	BDE *ΔH in kcal·mol⁻¹*	BDFE *ΔG in kcal·mol⁻¹*	HOMO (eV)	LUMO (eV)
	to *H*	to *G*							
**31c-anion**	0.317773	0.245982	-1373.242966	-1372.925193	-1372.996984				
**31d**	0.341689	0.258638	-1782.705182	-1782.363493	-1782.446544	58.9	81.1	-8.479	-2.121
**31d-anion**	0.323265	0.245308	-1577.750638	-1577.427373	-1577.50533				
**31e**	0.362071	0.264602	-2589.368727	-2589.006656	-2589.104125	47.1	67.7	-8.526	-1.868
**31e-anion**	0.342975	0.248073	-2384.432242	-2384.089267	-2384.184169				
**32a**	0.179724	0.132169	-477.4183266	-477.2386026	-477.2861576	82.3	106.1	-10.109	-0.594
**32a-anion**	0.159464	0.119574	-272.4245513	-272.2650873	-272.3049773				
**32b**	0.090708	0.053649	-359.4966208	-359.4059128	-359.4429718	81.5	105.1	-10.832	-0.593
**32b-anion**	0.070532	0.04087	-154.5042641	-154.4337321	-154.4633941				
**32c**	0.139961	0.092003	-689.5792659	-689.4393049	-689.4872629	68.1	91.4	-9.851	-0.6
**32c-anion**	0.121115	0.080077	-484.609627	-484.488512	-484.52955				
**32d**	0.312852	0.239967	-1264.746415	-1264.433563	-1264.506448	59.1	82.3	-8.474	-0.762
**32d-anion**	0.295003	0.22887	-1059.792128	-1059.497125	-1059.563258				
**32e**	0.338376	0.238596	-2275.954727	-2275.616351	-2275.716131	53.7	76.6	-8.874	-0.908
**32e-anion**	0.320426	0.226897	-2071.008905	-2070.688479	-2070.782008				
**32f**	0.329037	0.2347	-1878.264194	-1877.935157	-1878.029494	50.4	74.7	-9.056	-1.965
**32f-anion**	0.311334	0.225407	-1673.323887	-1673.012553	-1673.09848				
**33a**	0.061227	0.027693	-320.1852757	-320.1240487	-320.1575827	81.9	105.6	-10.986	-0.585
**33a-anion**	0.040821	0.014723	-115.1920237	-115.1512027	-115.1773007				
**33b**	0.079819	0.031738	-644.7802168	-644.7003978	-644.7484788	10.8	36.5	-11.251	-0.82
**33b-anion**	0.061535	0.024172	-439.9024513	-439.8409163	-439.8782793				
**34a**	0.119628	0.075706	-491.0434871	-490.9238591	-490.9677811	60.5	83.5	-10.992	-0.899
**34a-anion**	0.100108	0.062637	-286.0852496	-285.9851416	-286.0226126				

N°	Thermal correction		SP energy	*ΔH*	*ΔG*	BDE	BDFE	HOMO (eV)	LUMO (eV)
	to *H*	to *G*				*ΔH in kcal·mol⁻¹*	*ΔG in kcal·mol⁻¹*		
**34b**	0.138021	0.082112	-815.6501087	-815.5120877	-815.5679967	50.8	73.2	-11.584	-1.129
**34b-anion**	0.118421	0.067997	-610.707244	-610.588823	-610.639247				
**35**	0.127832	0.079984	-735.8701708	-735.7423388	-735.7901868	60.9	83.9	-10.954	-0.772
**35-anion**	0.108226	0.066741	-530.9112628	-530.8030368	-530.8445218				
**36**	0.07159	0.03195	-433.5288271	-433.4572371	-433.4968771	37.9	61.6	-10.726	-1.246
**36-anion**	0.054037	0.021901	-228.608593	-228.554556	-228.586692				
**37**	0.052406	0.003322	-1166.511598	-1166.459192	-1166.508276	-11.6	13.0	-11.837	-1.608
**37-anion**	0.035933	-0.004203	-961.671359	-961.635426	-961.675562				
**38a**	0.210047	0.150292	-1309.495991	-1309.285944	-1309.345699	14.9	38.7	-8.128	-1.248
**38a-anion**	0.193007	0.140993	-1104.612938	-1104.419931	-1104.471945				
**38b**	0.226965	0.148879	-1983.632371	-1983.405406	-1983.483492	11.2	34.9	-8.52	-1.327
**38b-anion**	0.209921	0.139166	-1778.755078	-1778.545157	-1778.615912				
**38c**	0.22082	0.147169	-1718.503946	-1718.283126	-1718.356777	8.9	32.6	-8.727	-1.838
**38c-anion**	0.203866	0.137719	-1513.630522	-1513.426656	-1513.492803				
**38d**	0.205827	0.141608	-1955.421457	-1955.21563	-1955.279849	28.8	53.5	-8.124	-1.38
**38d-anion**	0.189695	0.134613	-1750.517149	-1750.327454	-1750.382536				
**38e**	0.244184	0.167868	-2175.265998	-2175.021814	-2175.09813	10.1	33.5	-8.151	-1.701
**38e-anion**	0.227485	0.158161	-1970.390815	-1970.16333	-1970.232654				
**38f**	0.26107	0.167046	-2849.402127	-2849.141057	-2849.235081	7.8	31.0	-8.547	-1.775
**38f-anion**	0.244215	0.156949	-2644.530559	-2644.286344	-2644.37361				
**38g**	0.25482	0.165038	-2584.273291	-2584.018471	-2584.108253	6.1	29.3	-8.755	-1.886
**38g-anion**	0.238024	0.154966	-2379.404432	-2379.166408	-2379.249466				
**39a**	0.401864	0.315535	-2318.244785	-2317.842921	-2317.92925	24.8	47.4	-8.018	-1.266

N°	Thermal correction		SP energy	ΔH	ΔG	BDE ΔH in kcal·mol⁻¹	BDFE ΔG in kcal·mol⁻¹	HOMO (eV)	LUMO (eV)
	to *H*	to *G*							
**39a-anion**	0.384939	0.304224	-2113.345948	-2112.961009	-2113.041724				
**39b**	0.470681	0.350736	-4049.755933	-4049.285252	-4049.405197	3.9	26.6	-8.19	-1.607
**39b-anion**	0.453204	0.339078	-3844.889883	-3844.436679	-3844.550805				
**40**	0.035293	-0.002899	-485.3191305	-485.2838375	-485.3220295	6.7	31.2	-11.849	-1.93
**40-anion**	0.018534	-0.010987	-280.4493584	-280.4308244	-280.4603454				
**41a**	0.22265	0.178908	-534.1931961	-533.9705461	-534.0142881	36.9	59.7	-10.538	-1.789
**41a-anion**	0.203218	0.165506	-329.272621	-329.069403	-329.107115				
**41b**	0.211355	0.167967	-550.2170667	-550.0057117	-550.0490997	34.1	56.5	-8.555	-1.823
**41b-anion**	0.192303	0.154381	-345.3014145	-345.1091115	-345.1470335				
**41c**	0.247446	0.198437	-1049.554512	-1049.307066	-1049.356075	14.2	37.0	-10.88	-2.207
**41c-anion**	0.228706	0.185648	-844.6707409	-844.4420349	-844.4850929				
**42**	0.195099	0.139857	-845.6012841	-845.4061851	-845.4614271	90.5	113.9	-9.005	-0.936
**42-anion**	0.175161	0.127029	-640.594872	-640.419711	-640.467843				
**43**	0.143573	0.087653	-771.2065453	-771.0629723	-771.1188923	-17.3	6.0	-11.034	-3.71
**43-anion**	0.127612	0.078542	-566.3757677	-566.2481557	-566.2972257				
**44**	0.149044	0.082472	-1260.513866	-1260.364822	-1260.431394	10.8	33.1	-8.988	-1.559
**44-anion**	0.132796	0.071356	-1055.638022	-1055.505226	-1055.566666				
**45a**	0.137557	0.093286	-666.022397	-665.88484	-665.929111	61.3	85.1	-10.973	-1.745
**45a-anion**	0.120193	0.083536	-461.0650557	-460.9448627	-460.9815197				
**45b**	0.30996	0.242349	-1241.174937	-1240.864977	-1240.932588	46.9	69.1	-9.063	-1.925
**45b-anion**	0.291313	0.228761	-1036.239236	-1035.947923	-1036.010475				
**45c**	0.098394	0.058619	-682.8789193	-682.7805253	-682.8203003	15.4	38.7	-11.437	-2.328
**45c-anion**	0.081677	0.048693	-477.9953004	-477.9136234	-477.9466074				

N°	Thermal correction		SP energy	*ΔH*	*ΔG*	BDE *ΔH in kcal·mol⁻¹*	BDFE *ΔG in kcal·mol⁻¹*	HOMO (eV)	LUMO (eV)
	to *H*	to *G*							
**45d**	0.211316	0.154952	-1066.318801	-1066.107485	-1066.163849	6.8	29.9	-9.274	-2.969
**45d-anion**	0.194836	0.144991	-861.4491487	-861.2543127	-861.3041577				
**45e**	0.097003	0.054425	-2686.341213	-2686.24421	-2686.286788	27.0	50.9	-10.757	-2.002
**45e-anion**	0.080635	0.045737	-2481.439411	-2481.358776	-2481.393674				
**45f**	0.210786	0.152745	-3069.776124	-3069.565338	-3069.623379	16.6	41.6	-9.107	-2.691
**45f-anion**	0.193271	0.144772	-2864.889783	-2864.696512	-2864.745011				
**$NO_2^+(MeCN)_4BF_4^-$**	0.245112	0.145559	-1160.5421	-1160.296988	-1160.396541	4.0	-6.6	-10.955	-2.094
**BF_4^-**	0.019405	-0.013505	-424.659495	-424.64009	-424.673				
**$NO_2^+(MeCN)_4$**	0.223788	0.14022	-735.8742491	-735.6504611	-735.7340291			-11.113	-2.393
**MeCN**	0.049927	0.021442	-132.7519591	-132.7020321	-132.7305171				

2. Computed structures

1a

C	-2.616264	-0.273192	0.661868
C	-3.982395	-0.360192	0.801851
C	-4.824195	0.342435	-0.070414
C	-4.279284	1.121580	-1.093081
C	-2.901978	1.185849	-1.233831
C	-2.061399	0.503522	-0.360935
C	-0.610485	0.563667	-0.598632
N	0.266338	0.182312	0.479891
O	-0.107897	0.798594	-1.666865
N	0.172679	0.813718	1.685823
O	1.008996	0.522441	2.519299
O	-0.719953	1.625321	1.848188
H	-4.427559	-0.967433	1.580058
H	-4.911220	1.672650	-1.774777
C	1.540484	-0.467791	0.184105
H	1.731460	-1.234493	0.929940
H	1.436437	-0.927119	-0.794122
H	2.354874	0.257023	0.170058
H	-2.471328	1.784214	-2.027512
H	-1.976059	-0.827394	1.338372
O	-6.143727	0.205020	0.151423
C	-7.040362	0.901176	-0.708104
H	-6.880675	1.979491	-0.645471
H	-6.926337	0.566412	-1.741216
H	-8.039431	0.658770	-0.354975

1a-anion

C	-2.614268	0.188992	0.969482
C	-3.980522	0.185936	1.180268
C	-4.856108	-0.049643	0.116425
C	-4.342955	-0.283182	-1.155531
C	-2.961714	-0.277359	-1.344678
C	-2.075537	-0.042980	-0.301093
C	-0.581855	-0.042663	-0.576098
N	0.186213	0.254441	0.448311
O	-0.213809	-0.320039	-1.752014
H	-4.396080	0.363082	2.165673
H	-4.997331	-0.469248	-1.996309
C	1.601096	0.246476	0.124993
H	2.183390	0.506010	1.012466
H	1.948918	-0.733103	-0.231127
H	1.856477	0.964231	-0.666987
H	-2.553077	-0.457817	-2.331200
H	-1.944754	0.373757	1.799836
O	-6.180229	-0.032599	0.419628
C	-7.096443	-0.268039	-0.637661
H	-6.996158	0.486525	-1.421911
H	-6.956101	-1.263182	-1.067316
H	-8.089029	-0.199977	-0.197883

1b

C	-2.632589	-0.357542	0.667391
C	-4.003921	-0.473399	0.828827
C	-4.885069	0.207765	-0.011210
C	-4.356657	0.998063	-1.034771
C	-2.988844	1.103843	-1.216900
C	-2.120535	0.433361	-0.357554
C	-0.668989	0.507490	-0.630684
N	0.238933	0.238534	0.445415
O	-0.205672	0.662507	-1.730336
N	0.097086	0.875788	1.645074
O	0.920008	0.611070	2.499812
O	-0.815455	1.669356	1.775886
H	-4.398231	-1.103954	1.617343
H	-5.030171	1.530900	-1.696971
C	1.533368	-0.386245	0.186909
H	1.714258	-1.158498	0.929938
H	1.471655	-0.832372	-0.800577
H	2.332250	0.355028	0.210157
H	-2.584683	1.711201	-2.017689
H	-1.964979	-0.898710	1.328468
C	-6.371034	0.112394	0.182504
H	-6.879433	-0.026039	-0.773138
H	-6.631350	-0.714443	0.842840
H	-6.755823	1.034811	0.625464

1b-anion

C	-2.617738	0.148355	1.001406
C	-3.989241	0.151205	1.227515
C	-4.893465	-0.010137	0.178276
C	-4.378120	-0.181349	-1.106705
C	-3.007641	-0.188425	-1.332027
C	-2.103536	-0.020671	-0.283624
C	-0.611497	-0.031148	-0.579219
N	0.171819	0.234689	0.441408
O	-0.263434	-0.286229	-1.765793
H	-4.366766	0.278026	2.237085
H	-5.063172	-0.311824	-1.938798
C	1.582776	0.218399	0.102393
H	2.177025	0.457721	0.987589
H	1.916980	-0.758689	-0.272854
H	1.836545	0.946573	-0.680434
H	-2.612588	-0.325086	-2.331095
H	-1.932004	0.274750	1.829554
C	-6.379392	0.015085	0.414050
H	-6.875370	-0.807182	-0.105480
H	-6.610866	-0.059211	1.477101
H	-6.816205	0.945070	0.040351

1c

C	-2.583136	-0.486919	0.679142
C	-3.947226	-0.647837	0.865828
C	-4.846783	0.027936	0.047321
C	-4.383596	0.854176	-0.970462
C	-3.019530	1.002302	-1.175145
C	-2.121832	0.340203	-0.342813
C	-0.673931	0.461216	-0.643328
N	0.259642	0.284416	0.422561
O	-0.241953	0.582883	-1.759396
N	0.067661	0.899688	1.628341
O	0.886962	0.660744	2.493333
O	-0.879046	1.652869	1.749794
H	-4.309567	-1.299716	1.650830
H	-5.912198	-0.092466	0.202970
H	-5.084791	1.380112	-1.606363

C	1.582109	-0.286503	0.182040
H	1.773732	-1.071833	0.908972
H	1.563370	-0.705509	-0.818903
H	2.350031	0.483930	0.247280
H	-2.644603	1.636437	-1.969210
H	-1.882168	-1.015671	1.315853

1c-anion

C	-2.627843	-0.158892	0.836470
C	-4.003621	-0.217529	1.020097
C	-4.864614	0.188308	0.004459
C	-4.336046	0.652612	-1.194053
C	-2.957756	0.709111	-1.372696
C	-2.085402	0.305382	-0.363454
C	-0.584837	0.386428	-0.608756
N	0.176455	-0.007309	0.386361
O	-0.211285	0.819297	-1.734024
H	-4.406621	-0.581779	1.958329
H	-5.937780	0.143307	0.148334
H	-4.997866	0.971796	-1.991130
C	1.594548	0.091611	0.093995
H	2.173441	-0.237223	0.960313
H	1.890895	-0.526162	-0.764854
H	1.903981	1.117129	-0.149920
H	-2.534045	1.070371	-2.301430
H	-1.959286	-0.474482	1.627038

1d

C	-2.599639	-0.475355	0.679891
C	-3.963120	-0.626070	0.867462
C	-4.832870	0.063737	0.035382
C	-4.377621	0.884080	-0.985099
C	-3.011299	1.011163	-1.177641
C	-2.123385	0.343158	-0.341032
C	-0.673360	0.448582	-0.638159
N	0.256231	0.265537	0.428034
O	-0.242365	0.563318	-1.755075
N	0.070596	0.891846	1.631071
O	0.900327	0.668440	2.489100
O	-0.882779	1.635949	1.755019
H	-4.345200	-1.270361	1.648101
H	-5.077644	1.411061	-1.619923
C	1.582470	-0.294741	0.180881
H	1.791763	-1.062009	0.921815
H	1.554668	-0.736197	-0.810149
H	2.342224	0.485656	0.218867
H	-2.632577	1.640194	-1.973535
H	-1.912754	-1.013454	1.323415
Cl	-6.552405	-0.109877	0.279094

1d-anion

C	-2.595471	0.351663	1.002563
C	-3.959490	0.362720	1.259400
C	-4.832775	-0.023000	0.253865
C	-4.373959	-0.416279	-0.991410
C	-3.004306	-0.419189	-1.227196
C	-2.097784	-0.037175	-0.241477
C	-0.610074	-0.058893	-0.559302
N	0.184056	0.283361	0.427599
O	-0.282060	-0.399560	-1.729360
H	-4.339521	0.667898	2.226348
H	-5.071815	-0.715549	-1.763421
C	1.589998	0.246138	0.069390
H	2.197469	0.534943	0.930185
H	1.915914	-0.751574	-0.254837
H	1.830370	0.927367	-0.758290
H	-2.619095	-0.723093	-2.192120
H	-1.903155	0.650553	1.778574
Cl	-6.559144	-0.011549	0.567994

1e

C	-2.634556	-0.459573	0.645427
C	-4.002660	-0.551724	0.829668
C	-4.827318	0.198041	0.006697
C	-4.346186	1.018471	-0.999052
C	-2.976070	1.085512	-1.188320
C	-2.127639	0.359806	-0.359781
C	-0.666365	0.390162	-0.660119
N	0.256444	0.272985	0.409601
O	-0.248493	0.387350	-1.787066
N	0.041518	0.920433	1.600461
O	0.846072	0.705072	2.481987
O	-0.908958	1.672747	1.683127
H	-4.422139	-1.189434	1.594553
H	-5.025079	1.585519	-1.619845
C	1.579731	-0.314090	0.212270
H	1.752001	-1.075986	0.968280
H	1.576705	-0.765336	-0.774527
H	2.349226	0.455164	0.266624
H	-2.564185	1.708519	-1.971691
H	-1.970314	-1.039398	1.275606
N	-6.283105	0.115563	0.209830
O	-6.695703	-0.643231	1.063581
O	-6.994826	0.811084	-0.486488

1e-anion

C	-2.611951	0.154690	1.005056
C	-3.975114	0.165357	1.243591
C	-4.836018	0.001448	0.167353
C	-4.376080	-0.176257	-1.127720
C	-3.007111	-0.189766	-1.341913
C	-2.110525	-0.021881	-0.286973
C	-0.617832	-0.037829	-0.584891
N	0.155301	0.230890	0.439203
O	-0.276966	-0.297878	-1.770451
H	-4.369423	0.297848	2.241444
H	-5.075074	-0.301645	-1.942842
C	1.568594	0.217016	0.110039
H	2.155902	0.464764	0.996881
H	1.904804	-0.762462	-0.255866
H	1.821406	0.939656	-0.677539
H	-2.608871	-0.329767	-2.337917
H	-1.919064	0.282831	1.825290
N	-6.280085	0.014494	0.409051
O	-6.670680	0.144793	1.554662
O	-7.024853	-0.105387	-0.546340

2a

C	-2.485886	2.290451	0.199284
C	-3.701591	1.400320	-0.057764

H	-2.599578	2.866722	1.113619
H	-2.302308	2.962219	-0.640553
H	-4.187501	1.095902	0.870247
H	-4.424809	1.852311	-0.729900
N	-1.416663	1.308535	0.323608
N	-3.061925	0.241794	-0.686518
C	-1.678490	0.139600	-0.298834
O	-0.944929	-0.793058	-0.492833
H	-0.451319	1.575290	0.461183
N	-3.823372	-0.856742	-0.961571
O	-5.021962	-0.731640	-0.780289
O	-3.277705	-1.845035	-1.398255

2a-anion

C	-2.465373	2.300697	0.209433
C	-3.649214	1.385133	-0.134790
H	-2.679680	2.991742	1.025933
H	-2.156400	2.878827	-0.670288
H	-4.250163	1.200911	0.768411
H	-4.313597	1.836809	-0.875384
N	-1.470122	1.298229	0.573017
N	-3.042906	0.147324	-0.622754
C	-1.794570	0.121964	-0.186883
O	-0.927034	-0.766818	-0.334288
H	-0.503108	1.570815	0.448702

2b

C	1.922357	-0.533175	0.254924
C	2.607087	0.752187	-0.204943
N	4.117435	-0.995634	-0.373865
N	5.314808	-1.648431	-0.473143
C	2.882792	-1.642172	-0.099008
O	2.682638	-2.821273	-0.119338
O	5.305957	-2.827174	-0.747968
O	6.309136	-0.969112	-0.293385
C	4.099074	0.443326	-0.082894
H	2.363124	0.953354	-1.248101
H	0.951672	-0.723243	-0.198150
H	2.333184	1.616924	0.393738
H	1.808190	-0.564071	1.342714
H	4.712743	0.974966	-0.804921
H	4.482382	0.616769	0.924170

2b-anion

C	1.887701	-0.544696	0.021813
C	2.624633	0.786686	0.092596
N	4.209346	-1.025843	-0.007003
C	3.024636	-1.569538	0.196389
O	2.774286	-2.769356	0.476173
C	4.040623	0.381616	-0.363094
H	2.189198	1.578568	-0.518818
H	1.427765	-0.706711	-0.959428
H	2.659383	1.138013	1.127255
H	1.113814	-0.681448	0.777795
H	4.143023	0.523860	-1.449399
H	4.811946	1.002559	0.102994

2c

O	2.086635	-0.544530	0.337812
C	2.641177	0.722853	-0.088118
N	4.113418	-0.963645	-0.423351
N	5.291870	-1.661841	-0.515037
C	2.924732	-1.547638	0.077552
O	2.681694	-2.698957	0.250897
O	5.237633	-2.860929	-0.641684
O	6.300276	-0.985331	-0.497303
C	4.140960	0.475245	-0.176455
H	2.210308	0.965865	-1.057716
H	2.375018	1.470620	0.651440
H	4.604011	1.004724	-1.003860
H	4.661019	0.689412	0.757611

2c-anion

O	1.988478	-0.545215	-0.081208
C	2.622297	0.727409	-0.032397
N	4.215110	-1.007598	-0.010179
C	3.004523	-1.514255	0.068284
O	2.628766	-2.680048	0.242481
C	4.087389	0.406532	-0.343870
H	2.150611	1.395410	-0.753439
H	2.506059	1.146345	0.972811
H	4.312190	0.586502	-1.403951
H	4.767104	1.031235	0.240698

2d

C	-2.470876	2.315895	0.168229
C	-3.675888	1.417616	-0.096064
H	-2.601989	2.921607	1.059360
H	-2.227825	2.940291	-0.690836
H	-4.204328	1.164068	0.822517
H	-4.356389	1.849482	-0.822944
N	-1.416298	1.321210	0.375178
N	-3.027692	0.223886	-0.644689
C	-1.680186	0.084525	-0.249017
O	-0.951954	-0.838885	-0.405396
N	-3.804940	-0.866720	-0.971581
O	-5.001606	-0.692804	-0.879240
O	-3.252182	-1.870906	-1.346167
N	-0.130846	1.750818	0.624291
O	-0.001786	2.950818	0.742293
O	0.736234	0.920349	0.736974

2d-anion

C	-2.446753	2.241163	0.146504
C	-3.697339	1.414567	-0.158007
H	-2.609839	2.895620	1.003349
H	-2.197907	2.876930	-0.711380
H	-4.247020	1.159223	0.751728
H	-4.375067	1.877369	-0.872945
N	-1.379383	1.282705	0.406943
N	-3.068042	0.228812	-0.717372
C	-1.671452	0.117346	-0.096058
O	-1.069678	-0.947522	-0.139745
N	-3.845603	-0.845448	-0.942680
O	-5.054024	-0.723874	-0.739881
O	-3.332991	-1.861756	-1.380396

3a

C	1.866350	-0.565127	0.145446
C	2.588210	0.768212	-0.052041

N	4.137906	-0.973101	-0.217800
N	5.354899	-1.654697	-0.370719
C	2.922221	-1.637630	0.085916
O	2.824568	-2.811587	0.272876
O	5.318463	-2.668510	-1.017792
O	6.312862	-1.157263	0.161067
C	4.029945	0.436429	-0.335993
O	4.932971	1.158068	-0.630269
H	2.200870	1.353241	-0.884812
H	1.145805	-0.772831	-0.646549
H	2.569231	1.392236	0.842285
H	1.353142	-0.644100	1.102547

3a-anion

C	1.859057	-0.556297	0.149168
C	2.569248	0.771038	-0.059458
N	4.195036	-0.989906	-0.193473
C	2.993244	-1.578507	0.038860
O	2.802374	-2.787652	0.153231
C	4.028171	0.356047	-0.264265
O	4.921814	1.175563	-0.467296
H	2.229612	1.320796	-0.938432
H	1.104545	-0.776692	-0.607382
H	2.508397	1.445063	0.796165
H	1.385808	-0.655269	1.127225

3c

C	-3.597853	1.510313	-0.362466
H	-4.531021	1.312075	0.166882
H	-3.769861	2.156719	-1.222719
N	-1.544693	1.226871	0.688536
N	-2.921844	0.277941	-0.738119
C	-1.700311	0.054462	-0.097950
O	-0.964464	-0.871141	-0.148516
N	-3.595091	-0.683919	-1.463019
O	-4.709205	-0.366265	-1.813235
O	-3.027485	-1.720423	-1.691334
C	-2.606033	2.151927	0.578692
O	-2.673203	3.216424	1.100329
N	-0.383475	1.460365	1.453102
O	0.646807	1.053842	0.992198
O	-0.557158	2.052569	2.482375

3c-anion

C	-3.648674	1.462302	-0.349389
H	-4.504146	1.190010	0.270345
H	-3.967676	2.135432	-1.144742
N	-1.435624	1.290335	0.510864
N	-2.959130	0.300409	-0.860324
C	-1.628657	0.193955	-0.253158
O	-0.886947	-0.735932	-0.458363
N	-3.635657	-0.673310	-1.495303
O	-4.826149	-0.460127	-1.697374
O	-3.044805	-1.675707	-1.847603
C	-2.537571	2.076410	0.496626
O	-2.679420	3.145144	1.072000

4a

C	0.581616	-0.498847	0.400428
C	1.939193	-0.458909	0.152136
C	2.609650	0.739127	-0.048661
C	1.953617	1.953281	-0.012117
C	0.583537	1.924748	0.237986
C	-0.090383	0.720589	0.439695
H	0.064059	-1.437367	0.554162
H	2.481529	2.885592	-0.168309
H	0.030124	2.854559	0.277428
H	-1.156131	0.735278	0.630178
N	4.149102	-0.954836	-0.220305
N	5.342022	-1.650588	-0.382153
C	2.892115	-1.588663	0.056174
O	2.719179	-2.757350	0.204669
O	5.259279	-2.747958	-0.873513
O	6.344068	-1.090101	-0.015535
C	4.045901	0.474405	-0.294683
O	4.936963	1.224465	-0.542102

4a-anion

C	0.573194	-0.488953	0.406228
C	1.930263	-0.450131	0.157671
C	2.597317	0.743388	-0.047664
C	1.939843	1.956244	-0.014297
C	0.564914	1.934038	0.236609
C	-0.107293	0.731357	0.443999
H	0.052063	-1.425945	0.565857
H	2.463442	2.891498	-0.175715
H	0.010507	2.864453	0.271365
H	-1.173451	0.745821	0.637065
N	4.194663	-0.979051	-0.218442
C	2.976016	-1.537357	0.038132
O	2.730539	-2.731435	0.159507
C	4.045929	0.375797	-0.283573
O	4.928150	1.198437	-0.498240

4b

C	0.593118	-0.518893	0.367495
C	1.949658	-0.465510	0.126244
C	2.626415	0.733823	-0.034049
C	1.972655	1.950190	0.035217
C	0.605020	1.928182	0.276473
C	-0.042188	0.710455	0.434411
H	0.047678	-1.445342	0.494566
H	2.501261	2.886301	-0.091179
H	0.028308	2.841820	0.344338
N	4.155919	-0.963800	-0.253301
C	2.898678	-1.598992	-0.002694
O	2.712171	-2.769249	0.106925
C	4.060036	0.469896	-0.282189
O	4.956483	1.221821	-0.500682
F	-1.361205	0.727000	0.663566
N	5.346457	-1.661331	-0.433947
O	5.257761	-2.743926	-0.955198
O	6.350130	-1.115119	-0.052083

4b-anion

C	0.568023	-0.500138	0.371318
C	1.925397	-0.449515	0.130884
C	2.600061	0.745545	-0.031199
C	1.947983	1.960306	0.038532
C	0.573613	1.945748	0.280623

C	-0.070345	0.730320	0.438788
H	0.017485	-1.424028	0.501487
H	2.473595	2.899088	-0.089328
H	-0.002783	2.860029	0.347335
N	4.185604	-0.982028	-0.251573
C	2.965377	-1.541508	-0.018500
O	2.706383	-2.735243	0.064560
C	4.045923	0.376285	-0.272728
O	4.934028	1.198760	-0.456451
F	-1.401321	0.745451	0.671730

4c

C	0.597800	-0.516086	0.395696
C	1.951215	-0.467805	0.141803
C	2.619086	0.731042	-0.052173
C	1.972038	1.948569	-0.002290
C	0.605195	1.930560	0.253323
C	-0.038973	0.714993	0.442048
H	0.064203	-1.444520	0.545900
H	2.499131	2.881380	-0.153839
H	0.041287	2.851376	0.305705
N	4.154696	-0.964585	-0.253733
C	2.903396	-1.600607	0.028342
O	2.724088	-2.767627	0.167908
C	4.058397	0.461189	-0.313315
O	4.943294	1.216416	-0.558608
N	-1.491666	0.729348	0.705297
O	-2.043877	1.806627	0.774241
O	-2.048840	-0.339966	0.836977
N	5.349513	-1.663780	-0.432025
O	6.352188	-1.105222	-0.069382
O	5.257048	-2.755342	-0.930559

4c-anion

C	0.569961	-0.503095	0.370390
C	1.924089	-0.453799	0.131133
C	2.595636	0.743603	-0.030494
C	1.949709	1.961210	0.037822
C	0.577536	1.947997	0.279642
C	-0.072516	0.731129	0.438904
H	0.029272	-1.431048	0.498342
H	2.477663	2.897981	-0.090526
H	0.014706	2.868702	0.344502
N	4.184933	-0.979611	-0.251641
C	2.965593	-1.543350	-0.018302
O	2.709438	-2.735828	0.064864
C	4.047098	0.375382	-0.272279
O	4.927382	1.204445	-0.455413
N	-1.520252	0.746218	0.692827
O	-2.080004	1.822684	0.770391
O	-2.087898	-0.322164	0.813578

5a

C	0.601830	-0.496649	0.433816
C	1.953342	-0.463551	0.164894
C	2.615512	0.730367	-0.076451
C	1.953441	1.939107	-0.054643
C	0.580942	1.925784	0.220758
C	-0.088196	0.721024	0.459031
N	4.154627	-0.959675	-0.230329
C	2.918178	-1.594669	0.085903
O	2.758565	-2.757075	0.273007
C	4.054374	0.457618	-0.344255
O	4.938480	1.197843	-0.630658
Cl	2.781473	3.414340	-0.347822
Cl	-0.286195	3.407469	0.272513
Cl	-1.774029	0.731457	0.788640
Cl	-0.211275	-1.980365	0.725131
N	5.357778	-1.657784	-0.378394
O	5.274919	-2.758140	-0.856652
O	6.350619	-1.086364	-0.011996

5a-anion

C	0.573999	-0.478293	0.403574
C	1.926766	-0.448156	0.157089
C	2.593076	0.744644	-0.050561
C	1.931361	1.949653	-0.013699
C	0.553498	1.942418	0.239640
C	-0.120273	0.737803	0.445071
N	4.188671	-0.976704	-0.220692
C	2.982410	-1.543400	0.041327
O	2.742030	-2.731699	0.171230
C	4.052284	0.372630	-0.293041
O	4.930936	1.189094	-0.512768
Cl	2.764380	3.444274	-0.270520
Cl	-0.317736	3.432838	0.302858
Cl	-1.820237	0.748017	0.753979
Cl	-0.262247	-1.971485	0.657571

5b

C	0.590467	-0.485455	0.394760
C	1.941323	-0.464462	0.151110
C	2.612975	0.736453	-0.049301
C	1.947799	1.936292	-0.007416
C	0.579573	1.922280	0.240678
C	-0.089935	0.726750	0.437799
N	4.145163	-0.953015	-0.221849
N	5.345355	-1.650460	-0.381069
C	2.897022	-1.593142	0.056607
O	2.719783	-2.758252	0.207969
O	5.258822	-2.751054	-0.858834
O	6.342059	-1.079324	-0.023895
C	4.048841	0.471543	-0.299463
O	4.931904	1.226186	-0.549650
F	2.554614	3.095753	-0.188475
F	-0.093386	3.056928	0.294158
F	-1.389886	0.744771	0.668150
F	-0.086157	-1.604494	0.584349

5b-anion

C	0.583522	-0.472854	0.400752
C	1.932453	-0.454513	0.157148
C	2.600177	0.741532	-0.048258
C	1.931846	1.937423	-0.009842
C	0.563762	1.929013	0.239274
C	-0.103448	0.735706	0.441866
N	4.193739	-0.977609	-0.220373
C	2.986022	-1.545672	0.038586
O	2.738125	-2.733920	0.164782
C	4.055367	0.372891	-0.288249

O	4.928395	1.198419	-0.502905
F	2.527416	3.115506	-0.201119
F	-0.116811	3.071709	0.286509
F	-1.413225	0.754729	0.677140
F	-0.111143	-1.594222	0.600497

6

N	0.639919	-0.494227	0.391354
C	1.932662	-0.392667	0.150033
C	2.627177	0.783293	-0.052338
C	1.956057	1.992441	-0.013617
C	0.594195	1.915216	0.236386
C	-0.013260	0.671333	0.429376
H	2.461687	2.937762	-0.166615
H	-0.010828	2.810728	0.284420
H	-1.078103	0.617712	0.624298
N	4.129328	-0.946334	-0.216363
N	5.312281	-1.667145	-0.373169
C	2.865617	-1.558221	0.057331
O	2.658356	-2.718406	0.204493
O	5.206274	-2.765313	-0.855644
O	6.322449	-1.121827	-0.009609
C	4.056520	0.485298	-0.298418
O	4.961048	1.215147	-0.550602

6-anion

N	0.614240	-0.480619	0.407322
C	1.909179	-0.390785	0.160115
C	2.609203	0.779951	-0.055273
C	1.948508	1.988564	-0.022558
C	0.578871	1.928314	0.234697
C	-0.033550	0.695862	0.440527
H	2.455029	2.932831	-0.186378
H	-0.019568	2.829298	0.276698
H	-1.098322	0.651572	0.642250
N	4.152157	-0.989930	-0.212751
C	2.924184	-1.519959	0.049515
O	2.645139	-2.702767	0.179059
C	4.043361	0.368569	-0.289527
O	4.947581	1.162539	-0.511179

7

C	-3.011437	-0.998119	-0.284940
C	-4.374419	-0.767145	-0.177068
C	-4.853605	0.532239	-0.132406
C	-3.970888	1.598638	-0.188078
C	-2.606686	1.367780	-0.291903
C	-2.115192	0.065298	-0.342821
C	-0.657152	-0.209799	-0.574797
N	0.287732	0.129277	0.443994
O	-0.241697	-0.625035	-1.613243
N	1.596358	0.011109	0.159140
C	2.284203	-1.226661	0.202174
C	3.694966	-0.874125	-0.085671
C	3.798021	0.507428	-0.219528
C	2.459744	1.119659	-0.028103
O	2.110045	2.261581	-0.004572
O	1.755563	-2.270192	0.449746
C	4.796374	-1.697298	-0.204107
C	6.021170	-1.086083	-0.465865
C	6.124492	0.297376	-0.599450
C	5.006951	1.121027	-0.476335
N	-0.021204	0.130013	1.840954
O	0.921958	0.246473	2.572539
O	-1.179926	0.031997	2.140075
H	-5.059704	-1.602888	-0.122543
H	-5.916769	0.714624	-0.050552
H	-4.341481	2.614451	-0.153290
H	4.711991	-2.771537	-0.096655
H	6.910586	-1.695710	-0.564844
H	7.092230	0.740139	-0.799677
H	5.083010	2.196569	-0.576502
C	-2.503060	-2.413753	-0.304671
C	-1.663999	2.540291	-0.318386
F	-3.388957	-3.267122	0.212224
F	-2.225599	-2.842639	-1.542699
F	-1.372320	-2.537435	0.409670
F	-2.288180	3.682986	-0.603124
F	-0.694788	2.379412	-1.229879
F	-1.054156	2.715293	0.866167

7-anion

C	-2.892655	-0.975330	-0.279781
C	-4.254328	-0.811409	-0.060222
C	-4.790704	0.459535	0.065089
C	-3.961836	1.561603	-0.047377
C	-2.598852	1.392979	-0.269158
C	-2.030959	0.121571	-0.373691
C	-0.544025	-0.077323	-0.638794
N	0.193046	0.268364	0.409047
O	-0.206460	-0.519079	-1.745313
N	1.553112	0.078988	0.149287
C	2.197930	-1.151532	0.226728
C	3.643108	-0.875774	-0.038083
C	3.787539	0.489036	-0.230783
C	2.437490	1.118975	-0.102685
O	2.157052	2.289409	-0.188202
O	1.678629	-2.208424	0.483842
C	4.721350	-1.733064	-0.092672
C	5.972326	-1.169761	-0.354457
C	6.116956	0.201181	-0.549592
C	5.015902	1.058526	-0.488897
H	-4.896332	-1.679105	0.015726
H	-5.850334	0.589777	0.241049
H	-4.374766	2.558659	0.032458
H	4.604452	-2.798986	0.061501
H	6.845587	-1.808491	-0.405121
H	7.100488	0.608293	-0.750102
H	5.124253	2.125952	-0.638376
C	-2.381713	-2.382229	-0.445158
C	-1.763043	2.639312	-0.401683
F	-3.215400	-3.276593	0.118705
F	-2.283668	-2.739357	-1.737494
F	-1.186821	-2.579462	0.108568
F	-2.516260	3.686405	-0.793536
F	-0.790615	2.523397	-1.306859
F	-1.198052	3.013869	0.755812

12d

C	-0.525660	-0.289884	-1.778192

C	-1.429735	-1.110299	-1.116399
C	-1.476881	-1.070531	0.254766
C	0.250575	0.564902	0.295726
C	0.322080	0.552595	-1.078369
H	-2.098164	-1.776316	-1.646641
H	-2.147231	-1.668735	0.854839
H	0.870457	1.186439	0.925860
H	1.034617	1.199419	-1.570636
N	-0.638222	-0.239328	0.895068
N	-0.700440	-0.210516	2.400036
O	0.091385	0.508563	2.912049
O	-1.531976	-0.911991	2.871834
C	-0.493671	-0.332798	-3.290391
F	0.431910	0.478233	-3.791720
F	-1.674550	0.029646	-3.793548
F	-0.235178	-1.568205	-3.721490

8a

C	-1.353525	-1.555916	0.006737
C	-0.008860	-1.790558	0.012042
C	0.607825	-0.515691	0.002541
N	-1.443373	-0.171322	-0.005378
N	-2.614065	0.607534	-0.025605
O	-2.469574	1.797830	-0.027561
O	-3.649040	-0.010191	-0.039072
N	-0.267873	0.464504	-0.007413
H	0.469945	-2.755642	0.023103
C	2.070486	-0.260207	-0.005953
O	2.371466	1.028032	0.028542
C	3.774713	1.331895	0.009523
H	4.264637	0.894748	0.878549
H	4.227762	0.950026	-0.904385
H	3.840270	2.415233	0.041524
O	2.874638	-1.156567	-0.043155
C	-2.485614	-2.517738	0.014587
H	-3.127044	-2.367279	0.882825
H	-3.097461	-2.415422	-0.881787
H	-2.066563	-3.521967	0.048715

8a-anion

C	-1.324321	-1.515260	-0.011209
C	0.032626	-1.808852	-0.009507
C	0.639289	-0.543125	-0.006151
N	-1.499021	-0.172552	-0.008730
N	-0.298643	0.423962	-0.005568
H	0.510368	-2.776206	-0.010798
C	2.076291	-0.272993	-0.002946
O	2.382034	1.033523	-0.001165
C	3.777347	1.336396	0.003146
H	4.256589	0.931602	0.894545
H	4.261978	0.931885	-0.885452
H	3.845557	2.421338	0.003564
O	2.930813	-1.135798	-0.001830
C	-2.496556	-2.447232	-0.015074
H	-3.127266	-2.295966	0.864444
H	-3.123320	-2.293650	-0.897027
H	-2.163416	-3.485774	-0.015741

8b

C	-1.308713	-1.560325	-0.011500
C	0.029121	-1.806484	-0.009298
C	0.636078	-0.522069	-0.006333
N	-1.412407	-0.193979	-0.009833
H	-2.176550	-2.197683	-0.013916
N	-2.618300	0.533420	-0.011333
O	-2.527716	1.727718	-0.010672
O	-3.617541	-0.139175	-0.013153
N	-0.249642	0.452941	-0.006653
H	0.518723	-2.765594	-0.009724
C	2.096905	-0.255847	-0.003218
O	2.386884	1.034861	-0.000084
C	3.788220	1.349883	0.003604
H	4.260680	0.942845	0.896488
H	4.264743	0.945490	-0.888316
H	3.844143	2.434205	0.005313
O	2.906741	-1.147411	-0.003574

8b-anion

C	-1.301390	-1.513396	0.002419
C	0.053440	-1.811892	-0.000118
C	0.652387	-0.545329	0.002156
N	-1.487276	-0.174571	0.005855
H	-2.147855	-2.186155	0.001950
N	-0.289206	0.421748	0.005631
H	0.537700	-2.775185	-0.002972
C	2.089593	-0.271194	0.000798
O	2.390382	1.035726	0.004690
C	3.785207	1.343464	0.002359
H	4.269728	0.938320	0.890614
H	4.265877	0.942371	-0.889805
H	3.849355	2.428632	0.004704
O	2.945591	-1.131939	-0.004869

8c

C	-1.533319	-0.545891	0.020156
C	-0.171738	-0.548613	0.032303
C	0.181923	0.823756	0.013294
N	-1.884140	0.776273	-0.004385
H	-2.270280	-1.331543	0.027389
N	-3.201963	1.275145	-0.023846
O	-3.324695	2.465809	-0.040368
O	-4.062354	0.433158	-0.022397
N	-0.856740	1.624159	-0.008462
H	0.484461	-1.402913	0.051469
C	1.564058	1.392994	-0.005226
F	1.559480	2.720261	0.088264
F	2.206018	1.068530	-1.135295
F	2.297239	0.913297	1.005835

8c-anion

C	-1.523639	-0.441583	0.016712
C	-0.152775	-0.504267	0.262815
C	0.260683	0.788003	-0.056414
N	-1.873089	0.785246	-0.410930
H	-2.266962	-1.218545	0.130589
N	-0.766932	1.549116	-0.457889
H	0.440001	-1.334442	0.611954
C	1.625739	1.350470	-0.029255
F	1.711026	2.514155	0.647502
F	2.117751	1.623871	-1.259368

F	2.504416	0.510229	0.544195

8d

C	-1.561785	-0.552737	-0.000074
C	-0.192726	-0.536562	0.000114
C	0.138843	0.829856	-0.000098
N	-1.906975	0.791222	-0.000037
N	-3.212066	1.344050	-0.000082
O	-3.283127	2.537956	-0.000034
O	-4.108178	0.542304	-0.000261
N	-0.882908	1.639134	0.000074
H	0.472615	-1.382705	0.000181
N	1.482059	1.387508	-0.000306
O	2.391913	0.583637	-0.000311
O	1.606272	2.591100	-0.000184
C	-2.498469	-1.713842	-0.000124
H	-3.148942	-1.643943	0.874185
H	-3.148308	-1.644409	-0.874938
C	-1.743193	-3.036235	0.000509
H	-1.113062	-3.131880	0.886381
H	-2.456679	-3.859733	0.000448
H	-1.112381	-3.132335	-0.884829

8d-anion

C	-1.479700	-0.499919	-0.012715
C	-0.094955	-0.540636	-0.048416
C	0.237958	0.818029	-0.001124
N	-1.901021	0.792464	0.052951
N	-0.843166	1.603381	0.059769
H	0.566878	-1.388877	-0.097735
N	1.548919	1.371881	-0.003096
O	2.495036	0.590655	-0.045587
O	1.692534	2.585839	0.036466
C	-2.464550	-1.630321	-0.049594
H	-3.185298	-1.499365	0.762741
H	-3.041345	-1.573685	-0.978205
C	-1.806189	-3.000756	0.056600
H	-1.248884	-3.097929	0.991118
H	-2.555834	-3.792963	0.026707
H	-1.109212	-3.167581	-0.767701

8e

C	-1.566162	-0.555332	0.000119
C	-0.198041	-0.544328	-0.000189
C	0.138652	0.820681	-0.000055
N	-1.907684	0.788121	-0.000252
N	-3.213128	1.341140	-0.000569
O	-3.284026	2.534839	-0.000776
O	-4.108607	0.538803	-0.000577
N	-0.881446	1.632979	-0.000395
H	0.457341	-1.398681	-0.000157
N	1.483077	1.375273	0.000160
O	2.391040	0.569255	0.000329
O	1.609988	2.578525	0.000132
C	-2.499640	-1.708922	0.000372
H	-3.137489	-1.700629	0.884127
H	-3.138788	-1.699882	-0.882447
H	-1.899804	-2.617272	-0.000475

8e-anion

C	-1.505855	-0.488422	-0.009560
C	-0.122357	-0.550709	0.009930
C	0.231350	0.803102	-0.000019
N	-1.908776	0.810818	-0.030901
N	-0.838242	1.605700	-0.024788
H	0.522344	-1.413717	0.026373
N	1.550372	1.338839	0.005506
O	2.485267	0.543647	0.021882
O	1.710429	2.551220	-0.006108
C	-2.501581	-1.603411	0.002194
H	-3.056368	-1.630595	0.943244
H	-3.229100	-1.488557	-0.804134
H	-2.003930	-2.565517	-0.122362

8f

C	-1.531591	-0.543939	0.000049
C	-0.168491	-0.548219	0.000158
C	0.160163	0.823065	0.000013
N	-1.880767	0.779134	-0.000116
H	-2.268322	-1.330137	0.000038
N	-3.210468	1.277843	-0.000351
O	-3.331043	2.466302	-0.000436
O	-4.061882	0.430368	-0.000417
N	-0.867829	1.630480	0.000011
H	0.494527	-1.396170	0.000249
N	1.500848	1.385548	-0.000073
O	2.413157	0.584897	-0.000163
O	1.618912	2.589476	-0.000196

8f-anion

C	-1.506430	-0.444721	0.000061
C	-0.125058	-0.532079	0.000038
C	0.239521	0.816585	0.000005
N	-1.902234	0.853190	-0.000186
H	-2.236236	-1.241789	0.000089
N	-0.821860	1.632997	0.000025
H	0.512286	-1.400093	0.000085
N	1.566610	1.338958	0.000023
O	2.491993	0.534382	-0.000076
O	1.736890	2.549049	-0.000119

8g

C	-1.439381	-1.431870	0.335821
C	-0.119540	-1.739710	0.216330
C	0.532178	-0.486111	0.077749
N	-1.497342	-0.060421	0.251943
N	-0.310862	0.523596	0.117889
H	0.319044	-2.723966	0.200981
C	1.997022	-0.286453	-0.071518
O	2.331938	0.986374	-0.186423
C	3.739832	1.243659	-0.316917
H	4.268093	0.874730	0.561085
H	4.125221	0.763070	-1.214902
H	3.833630	2.322788	-0.391453
O	2.765545	-1.213171	-0.078529
C	-2.618724	-2.343737	0.419716
O	-3.439513	-2.163459	-0.598540
C	-4.639278	-2.958093	-0.572605
H	-4.386267	-4.016618	-0.591198
H	-5.214238	-2.724156	0.322527

H	-5.191543	-2.680500	-1.464909
O	-2.742152	-3.159990	1.290234
N	-2.631576	0.746110	0.513312
O	-3.582476	0.156221	0.956160
O	-2.521969	1.911315	0.269615

8g-anion

C	-1.347685	-1.469286	0.000323
C	0.006238	-1.790906	0.000021
C	0.619875	-0.541843	0.000841
N	-1.512840	-0.121522	0.000587
N	-0.314480	0.442957	0.001336
H	0.464935	-2.765982	-0.000485
C	2.063187	-0.285805	0.001054
O	2.374661	1.015680	0.002797
C	3.773109	1.311306	0.002911
H	4.250973	0.902425	0.892964
H	4.250838	0.903783	-0.887828
H	3.846641	2.395626	0.003747
O	2.903919	-1.159187	-0.000205
C	-2.463918	-2.419865	-0.000171
O	-3.664754	-1.830025	0.000238
C	-4.785282	-2.716948	-0.000236
H	-4.777503	-3.344521	-0.891115
H	-4.777562	-3.345412	0.890014
H	-5.666566	-2.080657	0.000045
O	-2.326815	-3.624369	-0.000757

8h

C	-1.518881	-0.552752	-0.047761
C	-0.159966	-0.571366	-0.050628
C	0.151290	0.800656	0.014180
N	-1.909754	0.757827	0.043180
N	-0.888468	1.591713	0.045974
H	0.497214	-1.426138	-0.046878
N	1.487107	1.376971	0.031092
O	2.403495	0.584834	-0.011857
O	1.584130	2.579604	0.084705
N	-2.411798	-1.684060	0.108862
O	-2.086688	-2.697414	-0.464980
O	-3.368409	-1.540739	0.834360
N	-3.223991	1.313943	-0.195153
O	-3.986060	0.573666	-0.741033
O	-3.367246	2.434929	0.175553

8h-anion

C	-1.480311	-0.494920	-0.008488
C	-0.097667	-0.576955	0.005644
C	0.219231	0.771715	-0.001189
N	-1.928117	0.775205	-0.022122
N	-0.869485	1.564328	-0.017932
H	0.545209	-1.439408	0.017622
N	1.537876	1.334622	0.004579
O	2.478281	0.554694	0.016407
O	1.660509	2.545876	-0.002859
N	-2.396363	-1.597807	-0.007667
O	-1.917249	-2.721810	0.004094
O	-3.592257	-1.370026	-0.018121

12d-anion

C	-0.527735	-0.286844	-1.788986
C	-1.438910	-1.089725	-1.119049
C	-1.459454	-1.028752	0.265016
C	0.217127	0.522434	0.313175
C	0.320278	0.537786	-1.073098
H	-2.115062	-1.745608	-1.653666
H	-2.156986	-1.640311	0.826392
H	0.864927	1.154161	0.910937
H	1.043045	1.176452	-1.562564
N	-0.649824	-0.239941	0.975458
C	-0.487204	-0.334274	-3.288811
F	0.449352	0.466875	-3.803148
F	-1.659769	0.033044	-3.827365
F	-0.235571	-1.573378	-3.737476

12b

C	-0.559272	-0.294367	-1.810485
C	-1.424204	-1.134506	-1.101779
C	-1.454778	-1.102591	0.265822
C	0.217522	0.590224	0.283485
C	0.265297	0.572365	-1.082334
H	-2.085623	-1.817874	-1.616985
H	-2.098852	-1.721440	0.872819
H	0.824009	1.233329	0.903765
H	0.949662	1.243187	-1.584345
N	-0.637239	-0.245645	0.908555
N	-0.684911	-0.215011	2.387737
O	0.009106	0.606226	2.898171
O	-1.410586	-1.015708	2.886805
C	-0.496385	-0.327240	-3.296100
H	0.382376	-0.907033	-3.595936
H	-1.381577	-0.796599	-3.720307
H	-0.374967	0.679509	-3.696320

12b-anion

C	-0.555917	-0.292682	-1.817741
C	-1.407990	-1.126799	-1.100349
C	-1.410365	-1.070675	0.286140
C	0.183583	0.552848	0.303813
C	0.254778	0.567514	-1.080144
H	-2.068793	-1.815606	-1.613618
H	-2.071751	-1.716664	0.854973
H	0.810789	1.218836	0.887958
H	0.936528	1.247126	-1.578383
N	-0.632095	-0.249343	0.993139
C	-0.491674	-0.329264	-3.316028
H	0.361315	-0.931683	-3.639402
H	-1.394746	-0.769175	-3.738645
H	-0.359186	0.673118	-3.725123

12e

C	-0.596088	-0.002150	-1.831723
C	-1.316585	-0.976449	-1.145712
C	-1.307486	-0.955017	0.227384
C	0.102667	0.962754	0.226122
C	0.120057	0.976303	-1.147000
H	-1.877493	-1.742633	-1.661984
H	-1.835713	-1.668334	0.844259
H	0.627280	1.679445	0.842194
H	0.684066	1.739502	-1.664323

N	-0.604015	0.005459	0.846146
C	-0.591044	-0.006987	-3.270889
N	-0.587263	-0.010707	-4.415743
N	-0.607866	0.009468	2.352981
O	-0.009444	0.907558	2.844000
O	-1.208493	-0.886104	2.845940

12e-anion

C	-0.595905	-0.002365	-1.847095
C	-1.310979	-0.969329	-1.149486
C	-1.280381	-0.916978	0.235813
C	0.075621	0.924387	0.234682
C	0.114831	0.968673	-1.150678
H	-1.870629	-1.736755	-1.666876
H	-1.825278	-1.652858	0.816573
H	0.616933	1.663649	0.814488
H	0.677745	1.733040	-1.669045
N	-0.604491	0.005688	0.921056
C	-0.591204	-0.006719	-3.286679
N	-0.587573	-0.010106	-4.433499

12c

C	-0.542492	-0.299886	-1.785460
C	-1.420610	-1.135210	-1.104798
C	-1.459361	-1.097289	0.266650
C	0.225912	0.586216	0.291412
C	0.285706	0.565345	-1.079735
H	-2.079515	-1.816226	-1.624140
H	-2.108510	-1.709068	0.875526
H	0.831354	1.224492	0.917983
H	0.978872	1.227072	-1.578993
N	-0.638399	-0.242970	0.904146
N	-0.692982	-0.210773	2.395479
O	0.004568	0.606246	2.903501
O	-1.426484	-1.006197	2.886997
H	-0.504837	-0.321654	-2.867134

12c-anion

C	-0.545078	-0.297216	-1.804191
C	-1.418857	-1.120881	-1.109432
C	-1.426396	-1.059346	0.277446
C	0.201125	0.540577	0.301196
C	0.283149	0.552135	-1.084518
H	-2.083844	-1.800266	-1.626853
H	-2.098008	-1.691387	0.849545
H	0.835926	1.192618	0.892549
H	0.981712	1.212749	-1.581594
N	-0.635356	-0.246509	0.981013
H	-0.509842	-0.316692	-2.886939

12f

C	-0.564536	-0.298852	-1.768354
C	-1.444650	-1.141274	-1.119132
C	-1.468200	-1.096005	0.255617
C	0.227987	0.573642	0.271476
C	0.283627	0.563626	-1.103014
H	-2.106765	-1.812132	-1.647616
H	-2.120725	-1.704457	0.865550
H	0.845668	1.207302	0.891895
H	0.972412	1.217149	-1.618746
N	-0.638646	-0.248161	0.880521
N	-0.688388	-0.212636	2.393474
O	0.015995	0.602471	2.885645
O	-1.424506	-1.004745	2.876827
N	-0.536395	-0.312894	-3.253688
O	0.150470	0.519923	-3.796356
O	-1.206021	-1.153221	-3.805956

12f-anion

C	-0.569561	-0.293368	-1.783537
C	-1.440064	-1.132499	-1.119691
C	-1.429349	-1.066583	0.267203
C	0.197870	0.541071	0.279932
C	0.271348	0.565121	-1.106444
H	-2.099871	-1.805514	-1.647877
H	-2.094764	-1.700339	0.842095
H	0.837685	1.191649	0.864998
H	0.955096	1.222198	-1.624064
N	-0.631508	-0.252102	0.957055
N	-0.536504	-0.314509	-3.260465
O	0.180680	0.488153	-3.819336
O	-1.228831	-1.132814	-3.827704

12a

C	-0.412385	-0.289725	-1.751947
C	-1.349294	-1.119494	-1.100750
C	-1.452460	-1.083565	0.250120
C	0.267149	0.565011	0.370835
C	0.405943	0.561104	-0.982975
H	-1.987115	-1.778349	-1.673565
H	-2.148358	-1.685572	0.814872
H	0.857393	1.190627	1.023840
H	1.140736	1.216025	-1.426271
N	-0.646055	-0.245038	0.952137
N	-0.777336	-0.213501	2.408326
O	-0.079668	0.575314	2.969147
O	-1.568294	-0.979814	2.866584
O	-0.369672	-0.368200	-3.054016
C	0.557177	0.463770	-3.777776
H	1.579143	0.211693	-3.496963
H	0.386031	0.236286	-4.825000
H	0.344324	1.514016	-3.580017

12a-anion

C	-0.432824	-0.291936	-1.758565
C	-1.369570	-1.104455	-1.116904
C	-1.469563	-1.039404	0.257525
C	0.170058	0.527456	0.397306
C	0.359283	0.547312	-0.978673
H	-2.000639	-1.767277	-1.695259
H	-2.193035	-1.662549	0.772887
H	0.778269	1.174132	1.021972
H	1.103607	1.203666	-1.405954
N	-0.717735	-0.239438	1.026244
O	-0.365715	-0.380413	-3.096705
C	0.577168	0.452376	-3.766076
H	1.596229	0.210720	-3.457656
H	0.457701	0.244546	-4.826245
H	0.368923	1.506576	-3.573292

13a

C	-0.151101	-0.510376	-1.536454
C	-1.284324	-1.118611	-0.984734
C	-1.590815	-0.881340	0.342995
C	0.317605	0.533272	0.569195
C	0.657836	0.323310	-0.754659
N	-0.781256	-0.073983	1.052900
N	-1.141488	0.179516	2.529377
O	-1.848782	1.110899	2.678095
O	-0.649223	-0.599472	3.264404
Cl	1.205985	1.517854	1.619257
Cl	2.039993	1.081111	-1.397701
Cl	0.242596	-0.784828	-3.161263
Cl	-2.288555	-2.135471	-1.911414
Cl	-2.930435	-1.551065	1.128042

13a-anion

C	-0.139935	-0.524347	-1.542416
C	-1.275740	-1.110912	-0.988882
C	-1.562027	-0.818439	0.341427
C	0.248459	0.527782	0.554943
C	0.649255	0.316784	-0.760615
N	-0.820394	-0.025280	1.078648
Cl	1.163616	1.562193	1.597628
Cl	2.058952	1.060500	-1.397999
Cl	0.282851	-0.835945	-3.174955
Cl	-2.280082	-2.150468	-1.914899
Cl	-2.950797	-1.504197	1.113773

13b

C	-0.149214	-0.511069	-1.548631
C	-1.233938	-1.168729	-0.981902
C	-1.531861	-0.933777	0.337028
C	0.274044	0.573547	0.522716
C	0.616909	0.365457	-0.789345
N	-0.781277	-0.073755	1.048726
N	-1.149170	0.185903	2.553661
O	-1.280601	1.327624	2.790863
O	-1.226162	-0.807259	3.173748
F	-2.534639	-1.491201	0.932948
F	-1.972243	-1.988232	-1.686829
F	0.150878	-0.714586	-2.793945
F	1.652802	0.977267	-1.305365
F	0.943789	1.360008	1.300587

13b-anion

C	-0.147349	-0.513308	-1.549874
C	-1.264450	-1.108242	-0.992298
C	-1.528923	-0.841843	0.336727
C	0.263817	0.487987	0.549192
C	0.642775	0.308636	-0.766147
N	-0.789552	-0.068437	1.086272
F	-2.596604	-1.395839	0.893917
F	-2.044093	-1.899538	-1.714121
F	0.161243	-0.724650	-2.812667
F	1.718607	0.894707	-1.270438
F	1.003338	1.270780	1.321823

20d''

C	4.040962	-0.766945	-1.011255
C	2.944527	-1.600903	-0.844276
C	1.804968	-1.105685	-0.228199
C	1.796979	0.208349	0.212407
C	2.891018	1.045166	0.053811
C	4.024047	0.551274	-0.569102
H	2.971983	-2.625142	-1.191203
H	0.932853	-1.732785	-0.083840
H	2.848265	2.065188	0.413466
H	4.889639	1.187937	-0.707564
N	0.616649	0.737606	0.838701
S	-0.570407	1.586071	-0.093082
O	0.147735	1.847228	-1.304931
O	-1.076122	2.650339	0.714416
C	-1.830155	0.392861	-0.397690
C	-2.989547	0.430612	0.359937
C	-1.618872	-0.542178	-1.405135
C	-3.976661	-0.510478	0.106247
H	-3.117851	1.176604	1.132336
C	-2.606475	-1.480566	-1.645934
H	-0.708457	-0.533652	-1.990944
C	-3.771680	-1.457231	-0.886054
H	-4.891211	-0.503764	0.683784
H	-2.471563	-2.223833	-2.422133
N	0.263460	0.238946	2.083972
O	-0.858339	0.476890	2.471507
O	1.112210	-0.378240	2.682481
C	-4.827647	-2.482692	-1.185024
F	-5.416632	-2.257943	-2.370282
F	-5.796878	-2.503740	-0.266475
F	-4.312833	-3.719246	-1.244999
C	5.284721	-1.264883	-1.686372
F	6.369768	-1.107731	-0.911996
F	5.536716	-0.595562	-2.824124
F	5.216346	-2.560482	-2.006031

20d''-anion

C	4.747475	-0.406915	-0.680438
C	4.061290	-1.617213	-0.632868
C	2.680874	-1.630794	-0.636553
C	1.925582	-0.438579	-0.688081
C	2.648225	0.777824	-0.737795
C	4.029794	0.784893	-0.732754
H	4.607141	-2.551894	-0.592166
H	2.147577	-2.573242	-0.597603
H	2.119257	1.720756	-0.779792
H	4.557613	1.732126	-0.772071
N	0.558331	-0.568505	-0.682989
S	-0.350749	0.725251	-0.758925
O	-0.278706	1.422892	-2.043960
O	-0.242465	1.608783	0.401833
C	-1.969956	-0.025829	-0.685245
C	-2.810037	0.281661	0.369093
C	-2.376821	-0.871506	-1.712451
C	-4.087502	-0.268128	0.408795
H	-2.469947	0.945772	1.152684
C	-3.644694	-1.421294	-1.672697
H	-1.706677	-1.098165	-2.532423
C	-4.492312	-1.113838	-0.609724
H	-4.753282	-0.033716	1.228914
H	-3.978459	-2.084438	-2.462303

C	-5.865842	-1.711288	-0.609157
F	-6.603913	-1.271752	-1.644154
F	-6.554527	-1.429947	0.502323
F	-5.833090	-3.050015	-0.718632
C	6.232010	-0.352674	-0.679945
F	6.731047	0.342118	0.365495
F	6.733642	0.257077	-1.776536
F	6.800192	-1.566647	-0.631874

20d

C	4.159445	-0.669888	-0.988264
C	3.100607	-1.554137	-0.838620
C	1.931234	-1.115571	-0.235923
C	1.854643	0.194834	0.209049
C	2.912453	1.080793	0.070065
C	4.076136	0.643281	-0.539238
H	3.179095	-2.573667	-1.191468
H	1.088527	-1.784638	-0.108832
H	2.817641	2.096787	0.431749
H	4.912618	1.320611	-0.663024
N	0.639183	0.671445	0.809220
S	-0.566922	1.465512	-0.157278
O	0.167879	1.728302	-1.360137
O	-1.104760	2.533712	0.626133
C	-1.787470	0.239297	-0.454879
C	-2.966831	0.269929	0.276952
C	-1.536236	-0.718002	-1.432091
C	-3.925875	-0.698132	0.020180
H	-3.123581	1.032648	1.027821
C	-2.503007	-1.683440	-1.668452
H	-0.613037	-0.702046	-1.998008
C	-3.690940	-1.672052	-0.944130
H	-4.854384	-0.692812	0.576283
H	-2.332466	-2.439587	-2.423999
H	-4.442644	-2.428109	-1.135506
N	0.278074	0.158376	2.042530
O	-0.854541	0.371236	2.414476
O	1.129021	-0.446403	2.653204
C	5.429370	-1.106497	-1.656363
F	6.499458	-0.927538	-0.865848
F	5.669155	-0.403450	-2.775972
F	5.412966	-2.396703	-2.004367

20d-anion

C	4.701850	-0.456548	-0.722840
C	3.989598	-1.651683	-0.667089
C	2.609550	-1.634700	-0.651025
C	1.878344	-0.426034	-0.690378
C	2.628198	0.774442	-0.751326
C	4.009408	0.750637	-0.765378
H	4.515613	-2.598066	-0.634376
H	2.056243	-2.565326	-0.605843
H	2.119070	1.728386	-0.784660
H	4.557452	1.686153	-0.810743
N	0.510617	-0.527849	-0.661205
S	-0.374613	0.786955	-0.733356
O	-0.312631	1.458392	-2.033773
O	-0.201445	1.689485	0.406250
C	-2.006468	0.084149	-0.593400
C	-2.797065	0.419517	0.493600
C	-2.477454	-0.760064	-1.592448
C	-4.083066	-0.104002	0.587892
H	-2.407177	1.084106	1.253920
C	-3.758654	-1.281668	-1.489828
H	-1.847042	-1.008305	-2.438096
C	-4.561836	-0.953579	-0.399867
H	-4.706620	0.154143	1.435197
H	-4.133246	-1.944318	-2.260447
H	-5.562210	-1.362487	-0.323998
C	6.186534	-0.433697	-0.737892
F	6.710227	0.253172	0.301063
F	6.690675	0.163187	-1.840867
F	6.730514	-1.658922	-0.692246

20b''

C	4.106785	-0.744152	-0.995480
C	2.970739	-1.549338	-0.868828
C	1.818860	-1.067333	-0.272005
C	1.805388	0.235611	0.210431
C	2.915225	1.054363	0.101858
C	4.062595	0.558648	-0.504092
H	2.994268	-2.565997	-1.245123
H	0.937187	-1.690595	-0.170723
H	2.876039	2.066280	0.486501
H	4.936182	1.194092	-0.591980
N	0.615173	0.760195	0.819500
S	-0.581113	1.581547	-0.135738
O	0.145123	1.825895	-1.348368
O	-1.082298	2.667955	0.649239
C	-1.835854	0.392918	-0.421597
C	-2.999959	0.429378	0.336233
C	-1.637694	-0.553146	-1.420689
C	-3.982978	-0.511406	0.083746
H	-3.127658	1.181105	1.103708
C	-2.631875	-1.490444	-1.649932
H	-0.730895	-0.549405	-2.012945
C	-3.810910	-1.485961	-0.902520
H	-4.899753	-0.492337	0.661533
H	-2.494864	-2.232417	-2.427736
N	0.250189	0.271759	2.060630
O	-0.872778	0.521404	2.443307
O	1.086477	-0.353615	2.672163
C	-4.874120	-2.518433	-1.139172
H	-4.768074	-2.975169	-2.122840
H	-4.799760	-3.310528	-0.388983
H	-5.869113	-2.079830	-1.054579
C	5.343520	-1.285578	-1.655430
H	5.665016	-2.210398	-1.172236
H	5.147687	-1.517418	-2.704910
H	6.161127	-0.566849	-1.609661

20b''-anion

C	4.791011	-0.375923	-0.698902
C	4.079539	-1.578381	-0.653063
C	2.696435	-1.603180	-0.635151
C	1.932504	-0.417866	-0.663033
C	2.652582	0.790347	-0.710316
C	4.044429	0.797328	-0.728297
H	4.624967	-2.517500	-0.630212
H	2.169566	-2.550711	-0.599374

H	2.122369	1.734034	-0.735915
H	4.560095	1.752224	-0.768522
N	0.552481	-0.558573	-0.638147
S	-0.359043	0.727654	-0.714723
O	-0.263683	1.450221	-1.989589
O	-0.268798	1.609914	0.454035
C	-1.978008	-0.016566	-0.671070
C	-2.833591	0.256207	0.383906
C	-2.387621	-0.832667	-1.718104
C	-4.108174	-0.298777	0.392842
H	-2.502568	0.901397	1.187826
C	-3.659877	-1.383288	-1.697390
H	-1.715787	-1.035043	-2.543996
C	-4.538272	-1.126905	-0.641705
H	-4.779829	-0.082240	1.216639
H	-3.980728	-2.019529	-2.514976
C	-5.907948	-1.745859	-0.608919
H	-6.235733	-2.030056	-1.609128
H	-5.906204	-2.647669	0.009337
H	-6.638978	-1.057184	-0.183016
C	6.296925	-0.362502	-0.699975
H	6.698397	-0.690974	0.262799
H	6.700912	-1.032710	-1.462382
H	6.678984	0.640671	-0.894327

20b

C	4.223156	-0.656972	-0.988505
C	3.123162	-1.514289	-0.890510
C	1.938735	-1.090302	-0.313480
C	1.855398	0.207198	0.176025
C	2.927802	1.077537	0.094596
C	4.108494	0.639406	-0.491274
H	3.200477	-2.525653	-1.273959
H	1.085266	-1.754376	-0.233873
H	2.834190	2.084634	0.482930
H	4.952126	1.316156	-0.561520
N	0.629192	0.669966	0.763023
S	-0.581699	1.453473	-0.198422
O	0.133536	1.695490	-1.417707
O	-1.109026	2.538886	0.570348
C	-1.814502	0.230478	-0.465810
C	-2.981867	0.269511	0.284467
C	-1.584179	-0.733461	-1.441313
C	-3.948977	-0.696347	0.049252
H	-3.122964	1.036447	1.034119
C	-2.558647	-1.696219	-1.657149
H	-0.669910	-0.724423	-2.021604
C	-3.734353	-1.676527	-0.913502
H	-4.868004	-0.684496	0.620916
H	-2.403066	-2.456836	-2.411466
H	-4.492157	-2.430487	-1.088413
N	0.279368	0.192512	2.012177
O	-0.854140	0.406164	2.385629
O	1.135871	-0.390761	2.636394
C	5.502116	-1.139961	-1.611756
H	5.946099	-1.934087	-1.006733
H	5.317528	-1.553364	-2.605275
H	6.227197	-0.331486	-1.700392

20b-anion

C	4.739934	-0.421945	-0.727082
C	4.004107	-1.609541	-0.673847
C	2.620954	-1.605676	-0.647689
C	1.882075	-0.404893	-0.674610
C	2.626167	0.788027	-0.731385
C	4.017803	0.766262	-0.756597
H	4.530423	-2.559522	-0.650964
H	2.074541	-2.541805	-0.605825
H	2.114974	1.742191	-0.755694
H	4.552871	1.710168	-0.801857
N	0.499138	-0.516706	-0.638364
S	-0.386173	0.786274	-0.710609
O	-0.330070	1.475826	-2.005389
O	-0.234873	1.694203	0.431563
C	-2.018417	0.075872	-0.585352
C	-2.819221	0.401959	0.497126
C	-2.481269	-0.764462	-1.591633
C	-4.104261	-0.126267	0.579999
H	-2.436677	1.063916	1.263486
C	-3.761609	-1.290858	-1.501496
H	-1.844275	-1.005655	-2.434373
C	-4.573913	-0.971826	-0.415573
H	-4.734441	0.125308	1.424463
H	-4.128569	-1.949973	-2.278886
H	-5.573599	-1.384082	-0.349094
C	6.245713	-0.439540	-0.739352
H	6.647144	-0.813898	0.206417
H	6.630381	-1.086723	-1.531190
H	6.646915	0.562563	-0.897406

20e’’

C	4.021293	-0.858781	-1.052161
C	2.921221	-1.692751	-0.846000
C	1.798499	-1.184455	-0.218880
C	1.801529	0.140256	0.197329
C	2.891508	0.973032	0.003007
C	4.014218	0.470849	-0.633202
H	2.948727	-2.721989	-1.178322
H	0.928064	-1.806749	-0.046008
H	2.856394	1.999140	0.345452
H	4.878648	1.099221	-0.802649
N	0.632393	0.682417	0.833322
S	-0.557359	1.532026	-0.095934
O	0.164098	1.811610	-1.301227
O	-1.076037	2.581020	0.722349
C	-1.807412	0.331551	-0.421537
C	-2.976705	0.359734	0.324674
C	-1.579099	-0.593395	-1.433058
C	-3.956685	-0.579080	0.052711
H	-3.116814	1.100311	1.100238
C	-2.557315	-1.535549	-1.697307
H	-0.662308	-0.575935	-2.008515
C	-3.736055	-1.520995	-0.951526
H	-4.881930	-0.585536	0.613046
H	-2.414304	-2.272200	-2.476501
N	0.271264	0.162443	2.070075
O	-0.854649	0.387899	2.451350
O	1.120298	-0.455773	2.666176
C	-4.750774	-2.502263	-1.229151
N	-5.559222	-3.284905	-1.452164

C	5.187899	-1.383051	-1.709421
N	6.118591	-1.798871	-2.236281

20e''-anion

C	3.537083	-1.404332	-0.805725
C	3.434454	-1.380546	0.592407
C	2.469582	-0.611232	1.197625
C	1.559843	0.174458	0.448693
C	1.687876	0.139877	-0.960578
C	2.654068	-0.637035	-1.568868
H	4.115651	-1.971952	1.192366
H	2.385936	-0.596309	2.277618
H	1.039464	0.741330	-1.581383
H	2.732066	-0.648206	-2.649582
N	0.634896	0.879269	1.176180
S	-0.481742	1.750614	0.471313
O	0.002135	2.657900	-0.563540
O	-1.289697	2.349927	1.521719
C	-1.568525	0.592087	-0.370959
C	-2.194215	-0.393014	0.386366
C	-1.763784	0.679441	-1.740507
C	-3.027728	-1.304766	-0.234520
H	-2.027297	-0.447035	1.455237
C	-2.599419	-0.230487	-2.372254
H	-1.265584	1.454597	-2.308342
C	-3.225296	-1.217902	-1.615121
H	-3.523673	-2.079972	0.335159
H	-2.765286	-0.178809	-3.440544
C	-4.088941	-2.165871	-2.263740
N	-4.779237	-2.924107	-2.780026
C	4.535835	-2.200591	-1.442384
N	5.342597	-2.842670	-1.954325

20e

C	4.110163	-0.766716	-1.037297
C	3.033843	-1.637320	-0.859299
C	1.884936	-1.173056	-0.245249
C	1.837179	0.145651	0.186750
C	2.903707	1.014399	0.019359
C	4.052914	0.556632	-0.603049
H	3.099207	-2.660987	-1.203468
H	1.033423	-1.826396	-0.096361
H	2.830254	2.035360	0.371438
H	4.898473	1.215504	-0.749556
N	0.641117	0.646640	0.804693
S	-0.562859	1.464820	-0.147954
O	0.169277	1.731343	-1.351442
O	-1.082982	2.530005	0.650779
C	-1.797505	0.254439	-0.450389
C	-2.969169	0.285608	0.293984
C	-1.564417	-0.691764	-1.442881
C	-3.939040	-0.670685	0.034164
H	-3.111967	1.039925	1.056059
C	-2.542158	-1.645238	-1.682373
H	-0.646791	-0.676447	-2.017850
C	-3.722432	-1.633413	-0.945640
H	-4.861826	-0.664904	0.599723
H	-2.385720	-2.392600	-2.449586
H	-4.482667	-2.380257	-1.139430
N	0.279626	0.122448	2.034481
O	-0.845320	0.351462	2.418389
O	1.124762	-0.504885	2.630114
C	5.303288	-1.245983	-1.680828
N	6.255039	-1.626963	-2.196479

20e-anion

C	4.729244	-0.354962	-0.463527
C	4.014816	-1.511665	-0.115976
C	2.641281	-1.510429	-0.147897
C	1.902277	-0.360263	-0.526487
C	2.646980	0.795606	-0.875617
C	4.025465	0.793255	-0.842197
H	4.552055	-2.405949	0.176841
H	2.090054	-2.403698	0.120788
H	2.132442	1.699720	-1.172150
H	4.571334	1.689864	-1.111535
N	0.543025	-0.475091	-0.517939
S	-0.355656	0.772280	-0.937089
O	-0.231237	1.120640	-2.352402
O	-0.243694	1.909629	-0.024508
C	-1.979754	0.078105	-0.711781
C	-2.735209	0.450002	0.388762
C	-2.467517	-0.821775	-1.651182
C	-4.003630	-0.095877	0.557027
H	-2.333468	1.160002	1.100602
C	-3.732505	-1.364451	-1.475259
H	-1.863752	-1.091897	-2.509322
C	-4.499713	-1.002277	-0.371332
H	-4.601864	0.188614	1.414092
H	-4.122085	-2.068445	-2.200524
H	-5.487369	-1.427374	-0.238236
C	6.154501	-0.349393	-0.429474
N	7.305887	-0.346162	-0.401292

20d'

C	3.877148	-1.106767	-1.081455
C	2.714124	-1.836332	-0.855889
C	1.630818	-1.237105	-0.230736
C	1.736480	0.089490	0.165015
C	2.888433	0.828417	-0.047049
C	3.965423	0.220932	-0.679290
H	4.720056	-1.577789	-1.572288
H	2.650007	-2.871047	-1.168301
H	0.715318	-1.786550	-0.039775
H	2.931152	1.860388	0.278879
H	4.873019	0.784207	-0.856229
N	0.615746	0.728496	0.801939
S	-0.559153	1.579354	-0.141586
O	0.152981	1.802294	-1.364783
O	-1.038394	2.671579	0.645448
C	-1.847899	0.409753	-0.422614
C	-2.981155	0.442485	0.374631
C	-1.682443	-0.507068	-1.455084
C	-3.982419	-0.489374	0.140923
H	-3.080397	1.178402	1.160723
C	-2.684278	-1.434696	-1.677552
H	-0.794011	-0.493536	-2.073160
C	-3.819330	-1.420560	-0.873524
H	-4.876188	-0.486962	0.750327
H	-2.582153	-2.163562	-2.472424

N	0.260270	0.286194	2.069917
O	-0.848890	0.571088	2.463569
O	1.097447	-0.332999	2.682427
C	-4.884834	-2.442946	-1.147105
F	-5.483180	-2.233509	-2.330350
F	-5.845515	-2.445715	-0.219541
F	-4.377158	-3.683587	-1.193213

20d'-anion

C	3.273076	-1.838778	-0.523970
C	3.218764	-1.537958	0.835891
C	2.335127	-0.585803	1.313546
C	1.464389	0.114500	0.454302
C	1.536569	-0.194996	-0.918503
C	2.424548	-1.158053	-1.386731
H	3.962537	-2.584550	-0.899674
H	3.871035	-2.053415	1.532256
H	2.293768	-0.361062	2.373564
H	0.910731	0.329538	-1.627753
H	2.450394	-1.371638	-2.449775
N	0.609576	1.025731	1.061092
S	-0.443719	1.835807	0.224375
O	0.084508	2.537853	-0.944113
O	-1.213080	2.656214	1.152330
C	-1.619969	0.645668	-0.441654
C	-2.153057	-0.311003	0.411864
C	-1.987215	0.693002	-1.778972
C	-3.068808	-1.232319	-0.074721
H	-1.850022	-0.340971	1.451675
C	-2.905673	-0.224687	-2.268403
H	-1.553289	1.440325	-2.430676
C	-3.439310	-1.179935	-1.412244
H	-3.487666	-1.982688	0.583175
H	-3.200336	-0.198704	-3.311050
C	-4.399298	-2.181091	-1.976165
F	-5.354536	-1.601863	-2.721362
F	-5.021902	-2.893406	-1.030181
F	-3.784944	-3.067026	-2.782183

20b'

C	3.885901	-1.086772	-1.086599
C	2.724443	-1.818492	-0.859790
C	1.642090	-1.222968	-0.229239
C	1.745975	0.102566	0.170980
C	2.896712	0.843007	-0.042599
C	3.973144	0.239531	-0.679830
H	4.727922	-1.554836	-1.581786
H	2.660723	-2.852172	-1.175798
H	0.728312	-1.775106	-0.038128
H	2.938471	1.874341	0.285434
H	4.879193	0.805067	-0.857745
N	0.625337	0.741469	0.807458
S	-0.564200	1.571245	-0.153738
O	0.163736	1.794032	-1.369362
O	-1.043252	2.670171	0.626702
C	-1.838029	0.401569	-0.431622
C	-2.982030	0.427054	0.359079
C	-1.674045	-0.521888	-1.456860
C	-3.974395	-0.504870	0.114404
H	-3.087112	1.164191	1.143831
C	-2.678866	-1.450511	-1.678944
H	-0.784109	-0.509964	-2.073716
C	-3.834757	-1.458701	-0.898017
H	-4.874279	-0.496424	0.718763
H	-2.565631	-2.176250	-2.475475
N	0.263151	0.287376	2.065674
O	-0.845819	0.574255	2.460096
O	1.094580	-0.342868	2.678250
C	-4.916582	-2.472734	-1.130505
H	-4.711578	-3.070984	-2.017531
H	-4.996095	-3.143966	-0.271928
H	-5.884700	-1.983122	-1.251626

20b'-anion

C	3.338409	-1.746685	-0.553736
C	3.273108	-1.472357	0.811464
C	2.358748	-0.558423	1.305326
C	1.466015	0.131808	0.458696
C	1.549529	-0.152047	-0.920020
C	2.468372	-1.077582	-1.404258
H	4.051729	-2.462970	-0.942242
H	3.941496	-1.978672	1.499343
H	2.310897	-0.354035	2.369221
H	0.908220	0.365199	-1.620467
H	2.501534	-1.270732	-2.471071
N	0.589320	1.008521	1.079677
S	-0.483870	1.810512	0.250577
O	0.043029	2.546418	-0.899274
O	-1.258142	2.607243	1.197246
C	-1.633591	0.617979	-0.435517
C	-2.212114	-0.320877	0.413969
C	-1.950642	0.627833	-1.783274
C	-3.112338	-1.242295	-0.094168
H	-1.953122	-0.330544	1.466750
C	-2.858965	-0.301702	-2.283992
H	-1.488172	1.355988	-2.438164
C	-3.452021	-1.245165	-1.451046
H	-3.561235	-1.974883	0.568425
H	-3.105633	-0.291774	-3.340082
C	-4.442263	-2.244173	-1.981639
H	-4.532168	-2.172572	-3.065529
H	-4.143981	-3.262930	-1.725530
H	-5.429851	-2.076604	-1.544875

20e'

C	4.074755	-0.846744	-1.032156
C	2.956049	-1.662315	-0.892500
C	1.810325	-1.169355	-0.285948
C	1.810280	0.138558	0.179029
C	2.916541	0.962602	0.051653
C	4.056214	0.461544	-0.562972
H	2.975161	-2.681370	-1.257506
H	0.926765	-1.786146	-0.164284
H	2.876758	1.977259	0.428302
H	4.929094	1.092797	-0.671834
N	0.625784	0.667364	0.800348
S	-0.559810	1.513377	-0.127649
O	0.150117	1.775437	-1.344074
O	-1.069633	2.578544	0.677072
C	-1.823403	0.320097	-0.432861

C	-2.984141	0.357719	0.326025
C	-1.612688	-0.610876	-1.442464
C	-3.971917	-0.577796	0.070783
H	-3.111291	1.102494	1.099792
C	-2.598409	-1.549735	-1.690647
H	-0.702199	-0.600595	-2.028040
C	-3.767882	-1.526457	-0.930639
H	-4.890258	-0.576946	0.642431
H	-2.467738	-2.291108	-2.467607
N	0.271361	0.180049	2.049667
O	-0.856033	0.408854	2.429365
O	1.122132	-0.421955	2.660310
C	-4.789945	-2.505073	-1.189585
N	-5.604378	-3.285747	-1.397535
H	4.966871	-1.235408	-1.507883

20e'-anion

C	3.415195	-1.313539	-1.080197
C	2.513845	-1.889361	-0.191665
C	1.591940	-1.099366	0.483792
C	1.549794	0.288199	0.292868
C	2.469887	0.857700	-0.599965
C	3.385693	0.063931	-1.277711
H	2.524686	-2.960447	-0.024414
H	0.886622	-1.546506	1.175998
H	2.456184	1.929578	-0.749790
H	4.088315	0.526224	-1.962183
N	0.637904	1.050419	1.046198
S	-0.480630	1.824555	0.277544
O	-0.049028	2.532241	-0.926703
O	-1.254173	2.605924	1.234061
C	-1.595440	0.554028	-0.333710
C	-2.642474	0.119535	0.470515
C	-1.345774	-0.041549	-1.564983
C	-3.455847	-0.915133	0.041732
H	-2.820696	0.597663	1.425409
C	-2.149319	-1.083994	-1.999086
H	-0.533476	0.316575	-2.184990
C	-3.202687	-1.515382	-1.193222
H	-4.281638	-1.258911	0.651547
H	-1.969486	-1.558746	-2.955310
C	-4.041485	-2.590652	-1.643095
N	-4.711162	-3.451071	-2.003697
H	4.134815	-1.927416	-1.607952

20c

C	4.044153	-0.936304	-1.059409
C	2.922386	-1.738124	-0.872853
C	1.791038	-1.216559	-0.263167
C	1.807321	0.106353	0.157618
C	2.917338	0.916457	-0.016481
C	4.042892	0.386818	-0.633424
H	4.924851	-1.347094	-1.537784
H	2.927777	-2.768813	-1.204416
H	0.905871	-1.822693	-0.104542
H	2.890538	1.943502	0.326251
H	4.917990	1.007468	-0.779696
N	0.636812	0.667972	0.776110
S	-0.567971	1.467025	-0.184029
O	0.151607	1.706679	-1.401010

O	-1.080949	2.553274	0.592300
C	-1.812985	0.258844	-0.459379
C	-2.966344	0.284634	0.313614
C	-1.606116	-0.681526	-1.463068
C	-3.941909	-0.671269	0.072972
H	-3.091414	1.035097	1.082520
C	-2.589385	-1.633919	-1.684579
H	-0.702633	-0.662658	-2.059732
C	-3.750385	-1.627899	-0.918071
H	-4.850429	-0.669294	0.661352
H	-2.451331	-2.376093	-2.460352
H	-4.515171	-2.373675	-1.097608
N	0.287690	0.207258	2.034572
O	-0.835696	0.448789	2.418826
O	1.140294	-0.385047	2.655162

20c-anion

C	3.470116	-1.354069	-1.032716
C	2.570084	-1.919578	-0.136047
C	1.627231	-1.126999	0.506786
C	1.561101	0.254172	0.276318
C	2.479892	0.812429	-0.626184
C	3.416471	0.016166	-1.271730
H	4.206416	-1.969676	-1.534835
H	2.598398	-2.985026	0.062713
H	0.922875	-1.566887	1.204690
H	2.448018	1.878935	-0.808208
H	4.117214	0.471167	-1.963119
N	0.631383	1.021047	0.999910
S	-0.495033	1.752860	0.194518
O	-0.048806	2.462524	-1.005453
O	-1.297288	2.534484	1.129561
C	-1.570580	0.456262	-0.423739
C	-2.589745	-0.027173	0.388050
C	-1.325640	-0.114418	-1.666730
C	-3.375368	-1.084322	-0.051633
H	-2.766201	0.430135	1.354225
C	-2.110378	-1.178909	-2.098923
H	-0.533943	0.279509	-2.292479
C	-3.134805	-1.662857	-1.294321
H	-4.176579	-1.456730	0.575430
H	-1.923503	-1.626145	-3.068024
H	-3.747777	-2.488699	-1.634922

20f'

C	4.133458	-0.874656	-1.072205
C	3.020583	-1.707210	-1.004799
C	1.848272	-1.251830	-0.419542
C	1.816267	0.035888	0.096579
C	2.915761	0.877490	0.039380
C	4.082306	0.414034	-0.553796
H	5.046344	-1.234443	-1.531070
H	3.065356	-2.710715	-1.408665
H	0.968429	-1.882075	-0.356114
H	2.850612	1.876404	0.453487
H	4.950279	1.058940	-0.607539
N	0.606476	0.522133	0.702400
S	-0.553556	1.426181	-0.193231
O	0.118108	1.613217	-1.444558
O	-0.969091	2.540599	0.598478

C	-1.898864	0.302790	-0.415751
C	-3.068070	0.489534	0.304501
C	-1.735880	-0.721825	-1.338608
C	-4.121478	-0.385760	0.093231
H	-3.156437	1.300161	1.014862
C	-2.784898	-1.601352	-1.545105
H	-0.811539	-0.831155	-1.891508
C	-3.950894	-1.408294	-0.823445
H	-5.052964	-0.274872	0.629485
H	-2.699491	-2.412395	-2.253920
N	0.271106	0.078249	1.969609
O	-0.839959	0.353890	2.368329
O	1.113730	-0.543071	2.571890
N	-5.073153	-2.341155	-1.045709
O	-4.935231	-3.198727	-1.892762
O	-6.070300	-2.198395	-0.369586

20f’-anion

C	3.376725	-1.272776	-1.076208
C	2.480365	-1.841476	-0.178023
C	1.565351	-1.045498	0.499650
C	1.525692	0.340963	0.301180
C	2.440516	0.903467	-0.601217
C	3.349446	0.103605	-1.281100
H	2.489638	-2.911671	-0.005126
H	0.863588	-1.486969	1.199090
H	2.428443	1.974518	-0.756956
H	4.048295	0.560308	-1.973128
N	0.620643	1.109738	1.056610
S	-0.501629	1.881578	0.292639
O	-0.081795	2.573398	-0.924403
O	-1.263942	2.674491	1.247952
C	-1.625139	0.605974	-0.292603
C	-2.667952	0.183875	0.524040
C	-1.382909	-0.007839	-1.516738
C	-3.484534	-0.857317	0.116449
H	-2.841624	0.675123	1.472844
C	-2.188128	-1.056887	-1.932558
H	-0.574689	0.340199	-2.147330
C	-3.224139	-1.459079	-1.105553
H	-4.307120	-1.196284	0.730176
H	-2.019677	-1.548315	-2.880485
N	-4.084437	-2.564782	-1.541962
O	-3.847690	-3.086447	-2.614543
O	-4.992255	-2.906838	-0.808651
H	4.090873	-1.891474	-1.605769

20a’

C	3.871767	-1.144019	-1.021583
C	2.696847	-1.855617	-0.800118
C	1.615683	-1.236396	-0.190588
C	1.733698	0.092643	0.194338
C	2.898210	0.812833	-0.013834
C	3.973497	0.185762	-0.629868
H	4.712561	-1.630026	-1.501351
H	2.621637	-2.892138	-1.104154
H	0.691572	-1.773104	-0.004811
H	2.951270	1.847603	0.301466
H	4.889913	0.735847	-0.803029
N	0.614196	0.757442	0.805739
S	-0.554157	1.583201	-0.190834
O	0.204483	1.787264	-1.391399
O	-1.036058	2.694576	0.570644
C	-1.828827	0.430035	-0.483120
C	-2.991730	0.465514	0.286720
C	-1.659996	-0.502272	-1.499071
C	-3.987282	-0.450021	0.032677
H	-3.106606	1.205069	1.067785
C	-2.657941	-1.428813	-1.749182
H	-0.758884	-0.503183	-2.099697
C	-3.825081	-1.405448	-0.980257
H	-4.904551	-0.447708	0.607510
H	-2.522544	-2.150694	-2.541190
N	0.220169	0.313958	2.057022
O	-0.894981	0.612382	2.425674
O	1.032297	-0.319085	2.693096
O	-4.847230	-2.257094	-1.147771
C	-4.727517	-3.259542	-2.154421
H	-3.877715	-3.913048	-1.948647
H	-5.649910	-3.832648	-2.111609
H	-4.622233	-2.807231	-3.142359

20a’-anion

C	3.378991	-1.698996	-0.354914
C	3.255823	-1.346244	0.988174
C	2.311903	-0.418321	1.391108
C	1.444594	0.207761	0.470674
C	1.588858	-0.152901	-0.884865
C	2.537087	-1.091639	-1.277352
H	4.115504	-2.426619	-0.672300
H	3.902094	-1.801888	1.730473
H	2.217693	-0.154468	2.438729
H	0.970277	0.315219	-1.637991
H	2.616689	-1.345169	-2.329068
N	0.532855	1.105385	1.003266
S	-0.562201	1.790414	0.098271
O	-0.051626	2.454539	-1.102049
O	-1.379127	2.627168	0.971462
C	-1.643091	0.495823	-0.499913
C	-2.260864	-0.346639	0.424337
C	-1.866318	0.317461	-1.852507
C	-3.096842	-1.353852	-0.010584
H	-2.081635	-0.211446	1.484847
C	-2.710208	-0.695145	-2.304534
H	-1.379076	0.970681	-2.565828
C	-3.326660	-1.533561	-1.380126
H	-3.584165	-2.018435	0.692528
H	-2.870845	-0.817806	-3.366470
O	-4.158509	-2.548071	-1.709064
C	-4.434075	-2.752650	-3.087869
H	-3.521727	-2.993276	-3.638623
H	-5.118824	-3.596082	-3.134791
H	-4.909504	-1.872453	-3.527037

20f

C	4.137440	-0.686303	-0.987847
C	3.088937	-1.582425	-0.853989
C	1.925470	-1.142770	-0.247789
C	1.853593	0.168110	0.204999
C	2.911288	1.053366	0.069840

C	4.077774	0.621931	-0.540476
H	3.179174	-2.595449	-1.218953
H	1.081300	-1.810250	-0.124261
H	2.819333	2.067516	0.436588
H	4.920000	1.287321	-0.664405
N	0.641279	0.645415	0.808987
S	-0.557517	1.462237	-0.152095
O	0.182124	1.723893	-1.352068
O	-1.081237	2.530042	0.640470
C	-1.790025	0.250119	-0.454172
C	-2.971141	0.294274	0.274119
C	-1.544837	-0.711361	-1.428873
C	-3.938750	-0.664878	0.016393
H	-3.122629	1.060116	1.022919
C	-2.520286	-1.667696	-1.665926
H	-0.619748	-0.705874	-1.992026
C	-3.710098	-1.643240	-0.944908
H	-4.868775	-0.649428	0.569761
H	-2.354795	-2.427282	-2.419103
H	-4.468268	-2.392756	-1.136434
N	0.272970	0.113527	2.032565
O	-0.853754	0.341870	2.411209
O	1.114042	-0.519819	2.627754
N	5.374860	-1.148178	-1.640238
O	5.403480	-2.286257	-2.062586
O	6.300038	-0.366082	-1.721507

20f-anion

C	3.648103	-1.204118	-0.782806
C	3.521601	-1.295206	0.604947
C	2.520436	-0.599643	1.233635
C	1.601505	0.212532	0.515666
C	1.776055	0.291121	-0.892126
C	2.778672	-0.411108	-1.524811
H	4.205071	-1.912807	1.171757
H	2.405058	-0.666864	2.308521
H	1.129609	0.920297	-1.486438
H	2.896206	-0.343751	-2.598172
N	0.635170	0.821071	1.257390
S	-0.511264	1.685100	0.567546
O	-0.023673	2.716606	-0.342147
O	-1.393133	2.139020	1.632164
C	-1.470408	0.550220	-0.432426
C	-2.012459	-0.577406	0.175001
C	-1.664640	0.798587	-1.782329
C	-2.759967	-1.465236	-0.584463
H	-1.847041	-0.758199	1.230789
C	-2.418229	-0.094492	-2.537626
H	-1.227613	1.680229	-2.234449
C	-2.963032	-1.223670	-1.940835
H	-3.184702	-2.346634	-0.119826
H	-2.575168	0.093407	-3.592787
H	-3.546897	-1.919246	-2.531556
N	4.688257	-1.938113	-1.452525
O	5.449164	-2.621768	-0.779760
O	4.775493	-1.855387	-2.670788

20f’’

C	4.016436	-0.764637	-1.037024
C	2.944151	-1.623026	-0.853984
C	1.815854	-1.132968	-0.220800
C	1.803614	0.187725	0.207859
C	2.884271	1.035342	0.024344
C	4.015256	0.552855	-0.613498
H	2.989548	-2.645259	-1.201108
H	0.952759	-1.767164	-0.056247
H	2.837310	2.058028	0.374990
H	4.875564	1.185544	-0.778590
N	0.626322	0.711436	0.843400
S	-0.566460	1.557815	-0.081471
O	0.146967	1.831566	-1.292464
O	-1.083811	2.608539	0.734562
C	-1.817841	0.353027	-0.393993
C	-2.983957	0.386955	0.356497
C	-1.593834	-0.577425	-1.401117
C	-3.968469	-0.551014	0.093026
H	-3.121127	1.130871	1.129191
C	-2.574682	-1.520190	-1.658854
H	-0.679422	-0.565157	-1.980318
C	-3.735260	-1.481501	-0.904660
H	-4.892905	-0.558025	0.652297
H	-2.443356	-2.262306	-2.433189
N	0.272810	0.190587	2.081988
O	-0.851752	0.414039	2.467691
O	1.126132	-0.426278	2.672616
N	-4.783254	-2.482894	-1.186533
O	-4.563724	-3.301998	-2.053947
O	-5.805071	-2.429895	-0.535031
N	5.216975	-1.278499	-1.719475
O	5.187187	-2.416915	-2.140498
O	6.170813	-0.534922	-1.824980

20f’’-anion

C	3.526086	-1.273613	-0.798347
C	3.451312	-1.279476	0.595011
C	2.483185	-0.531035	1.216438
C	1.554402	0.252491	0.483291
C	1.670597	0.238098	-0.930377
C	2.638828	-0.517720	-1.556137
H	4.148075	-1.871307	1.172880
H	2.407783	-0.529950	2.296791
H	1.009897	0.839276	-1.537769
H	2.714847	-0.520012	-2.635162
N	0.629660	0.931012	1.224643
S	-0.509121	1.798216	0.542088
O	-0.047412	2.721801	-0.486519
O	-1.311389	2.370924	1.609714
C	-1.585606	0.628884	-0.297475
C	-2.186834	-0.371221	0.460070
C	-1.795069	0.723121	-1.664518
C	-3.009885	-1.295079	-0.158101
H	-2.009852	-0.429534	1.526856
C	-2.621926	-0.196279	-2.295632
H	-1.315276	1.509166	-2.232919
C	-3.208175	-1.185211	-1.526861
H	-3.489334	-2.083867	0.403905
H	-2.803256	-0.146987	-3.359935
N	-4.078132	-2.168169	-2.190349
O	-4.265576	-2.050292	-3.385062
O	-4.564861	-3.049069	-1.509407

N	4.538075	-2.057083	-1.460478
O	5.319538	-2.702310	-0.775130
O	4.579271	-2.048866	-2.683124

20a

C	4.243514	-0.713595	-0.877455
C	3.145340	-1.572526	-0.804253
C	1.957056	-1.111466	-0.254964
C	1.871381	0.186949	0.217677
C	2.963221	1.044723	0.154189
C	4.147188	0.595308	-0.392917
H	3.203137	-2.588575	-1.167528
H	1.095868	-1.767115	-0.189566
H	2.874496	2.056756	0.530576
H	5.012757	1.242699	-0.456903
N	0.638315	0.667497	0.765640
S	-0.550371	1.433169	-0.235879
O	0.185903	1.637102	-1.449835
O	-1.084448	2.543603	0.491795
C	-1.785364	0.209926	-0.491958
C	-2.965010	0.270922	0.237039
C	-1.541858	-0.778651	-1.439103
C	-3.931286	-0.697756	0.008894
H	-3.116159	1.056248	0.965407
C	-2.515565	-1.743623	-1.648131
H	-0.617729	-0.785923	-2.003556
C	-3.703813	-1.701905	-0.925708
H	-4.860045	-0.669053	0.564016
H	-2.349804	-2.522655	-2.381181
H	-4.461252	-2.457465	-1.095293
N	0.270178	0.248642	2.031283
O	-0.869557	0.476367	2.376733
O	1.117367	-0.303886	2.694728
O	5.437525	-1.059663	-1.399155
C	5.571572	-2.367747	-1.942472
H	4.861665	-2.525564	-2.757276
H	6.586481	-2.428254	-2.327376
H	5.428101	-3.127849	-1.171638

20a-anion

C	3.644993	-0.897206	-1.103749
C	2.852955	-1.582566	-0.189461
C	1.868558	-0.894947	0.519774
C	1.652579	0.473059	0.352877
C	2.473786	1.144968	-0.568074
C	3.446094	0.472314	-1.286649
H	2.981857	-2.643255	-0.020488
H	1.248071	-1.435402	1.226470
H	2.332353	2.208074	-0.715399
H	4.073887	0.999899	-1.995873
N	0.673488	1.118941	1.138038
S	-0.525837	1.781396	0.378123
O	-0.157680	2.651391	-0.741538
O	-1.425073	2.375012	1.362869
C	-1.426515	0.429530	-0.385093
C	-2.360050	-0.281300	0.360305
C	-1.110355	0.036554	-1.679775
C	-2.984758	-1.389023	-0.197819
H	-2.596083	0.036876	1.368783
C	-1.731809	-1.079167	-2.230835
H	-0.388100	0.605763	-2.252124
C	-2.668362	-1.791716	-1.491942
H	-3.719420	-1.939453	0.377704
H	-1.484712	-1.387907	-3.239574
H	-3.153513	-2.658753	-1.923934
O	4.632715	-1.467883	-1.850966
C	4.849044	-2.857821	-1.682024
H	3.959344	-3.432928	-1.952569
H	5.666052	-3.121078	-2.350422
H	5.134243	-3.091405	-0.652741

20a’’

C	4.114148	-0.808799	-0.872506
C	2.974617	-1.609383	-0.772623
C	1.821764	-1.083458	-0.206277
C	1.811729	0.220577	0.259295
C	2.945111	1.019759	0.169291
C	4.093868	0.506516	-0.396810
H	2.972527	-2.630008	-1.127535
H	0.929383	-1.693857	-0.121817
H	2.915035	2.037475	0.538932
H	4.990420	1.107695	-0.481736
N	0.615667	0.772350	0.823100
S	-0.547910	1.582139	-0.186136
O	0.225962	1.810670	-1.372667
O	-1.070543	2.680553	0.568788
C	-1.794262	0.404009	-0.505357
C	-2.984771	0.441045	0.219616
C	-1.576503	-0.546390	-1.494598
C	-3.961855	-0.488528	-0.054107
H	-3.133500	1.192459	0.983466
C	-2.556138	-1.487167	-1.764926
H	-0.652448	-0.550249	-2.059525
C	-3.752820	-1.459886	-1.042770
H	-4.899599	-0.484960	0.486604
H	-2.384688	-2.223081	-2.536953
N	0.208880	0.315009	2.063022
O	-0.928624	0.567501	2.399388
O	1.025220	-0.289333	2.721534
O	-4.760822	-2.324204	-1.233902
C	-4.602708	-3.326977	-2.234395
H	-3.754992	-3.974420	-2.002886
H	-5.521375	-3.907324	-2.219623
H	-4.470663	-2.874417	-3.219156
O	5.279917	-1.219695	-1.411820
C	5.333253	-2.531340	-1.959602
H	4.599950	-2.650112	-2.760215
H	6.335502	-2.644435	-2.365500
H	5.165825	-3.284959	-1.187210

20a’’-anion

C	3.115771	-1.120066	-1.132810
C	2.414994	-1.549091	-0.011242
C	1.609561	-0.646590	0.682715
C	1.484676	0.687669	0.295123
C	2.210155	1.097887	-0.835545
C	3.007223	0.211406	-1.537800
H	2.479372	-2.572620	0.332457
H	1.061146	-0.985508	1.555097
H	2.137112	2.129405	-1.156421

H	3.565433	0.536596	-2.408566
N	0.694868	1.568013	1.066661
S	-0.600930	2.123543	0.380559
O	-0.403954	2.711015	-0.947426
O	-1.299273	2.973561	1.342035
C	-1.660209	0.709940	0.089950
C	-2.310801	0.111761	1.168808
C	-1.764342	0.150037	-1.170808
C	-3.067032	-1.026546	0.977217
H	-2.222357	0.545082	2.158425
C	-2.520407	-1.003029	-1.376886
H	-1.256195	0.616945	-2.005558
C	-3.175586	-1.591369	-0.299304
H	-3.584416	-1.500334	1.802844
H	-2.588994	-1.422080	-2.371043
O	-3.938942	-2.706287	-0.387801
C	-4.072832	-3.311679	-1.666027
H	-3.104236	-3.648573	-2.043267
H	-4.725056	-4.170162	-1.524346
H	-4.527212	-2.622598	-2.381844
O	3.928270	-1.913506	-1.887225
C	4.043609	-3.272800	-1.504259
H	3.077168	-3.781497	-1.556125
H	4.729941	-3.729876	-2.213977
H	4.452412	-3.367162	-0.494718

21d’

C	-4.883924	-1.335718	0.789532
C	-4.733013	-0.327776	-0.151501
C	-3.497616	-0.139555	-0.753058
C	-2.455642	-0.976352	-0.386126
C	-2.592943	-1.992667	0.550324
C	-3.828601	-2.167173	1.148144
H	-5.567335	0.305642	-0.421570
H	-3.353175	0.635005	-1.494107
H	-1.756080	-2.630493	0.802208
H	-3.969667	-2.949480	1.883361
S	-0.888651	-0.756260	-1.148881
N	-0.001844	0.233343	-0.013701
S	1.065800	-0.374559	1.229273
O	-0.158940	-1.983271	-1.155799
O	-1.009994	0.050238	-2.320801
O	0.547998	-1.677067	1.500887
O	1.078065	0.649287	2.224553
C	2.633129	-0.487750	0.441598
C	2.947790	-1.657981	-0.231792
C	3.504048	0.589963	0.535653
C	4.186468	-1.748111	-0.849890
H	2.247788	-2.481898	-0.270180
C	4.736979	0.487579	-0.083220
H	3.228117	1.480761	1.083312
C	5.062731	-0.676448	-0.771133
H	4.459964	-2.649333	-1.381868
H	5.441683	1.308388	-0.027081
N	-0.114925	1.632402	-0.155204
O	0.891557	2.271074	0.009175
O	-1.209972	2.050530	-0.428081
C	-6.205903	-1.524833	1.477807
F	-7.216177	-0.961758	0.809182
F	-6.201832	-0.982802	2.706568
F	-6.507524	-2.820303	1.633091
C	6.406805	-0.739337	-1.439241
F	7.396656	-0.442959	-0.584538
F	6.498853	0.143791	-2.446273
F	6.673752	-1.943339	-1.949510

21d’-anion

C	-4.327076	-1.683552	0.696496
C	-4.267152	-0.312423	0.459817
C	-3.145497	0.223965	-0.146101
C	-2.100081	-0.619496	-0.504346
C	-2.156263	-1.981134	-0.270391
C	-3.284093	-2.521507	0.336606
H	-5.093321	0.327805	0.745860
H	-3.082631	1.287738	-0.339999
H	-1.326311	-2.613700	-0.555861
H	-3.340926	-3.585068	0.527511
S	-0.668434	0.088231	-1.307002
N	0.001460	1.098929	-0.256964
S	0.798074	0.497289	0.998257
O	0.192608	-1.024198	-1.668426
O	-1.181510	0.910320	-2.386386
O	0.108615	-0.603203	1.648335
O	1.164334	1.632550	1.823295
C	2.330780	-0.170609	0.367815
C	2.627187	-1.507910	0.556345
C	3.208574	0.682851	-0.290474
C	3.831222	-2.012594	0.078104
H	1.922251	-2.151407	1.065880
C	4.404542	0.180841	-0.770558
H	2.957549	1.727057	-0.431121
C	4.706223	-1.165786	-0.582746
H	4.075102	-3.057423	0.218982
H	5.100083	0.829968	-1.289316
C	-5.562095	-2.231182	1.342852
F	-6.653138	-2.039120	0.581061
F	-5.821514	-1.635389	2.519298
F	-5.484495	-3.544517	1.582619
C	6.018664	-1.671595	-1.097620
F	7.056807	-1.164681	-0.408324
F	6.220712	-1.328337	-2.380299
F	6.125313	-3.002755	-1.028457

21d

C	-4.813060	-1.427545	0.778218
C	-4.702675	-0.393022	-0.137759
C	-3.474188	-0.140496	-0.731603
C	-2.399647	-0.942216	-0.383240
C	-2.496252	-1.985386	0.529281
C	-3.724453	-2.225404	1.117738
H	-5.561161	0.213027	-0.393945
H	-3.359589	0.657456	-1.452803
H	-1.633324	-2.592192	0.769955
H	-3.835666	-3.030891	1.833496
S	-0.844162	-0.643681	-1.145142
N	0.005230	0.360328	-0.001870
S	1.116170	-0.224652	1.225897
O	-0.068902	-1.842388	-1.177738
O	-1.007030	0.173102	-2.305709
O	0.636023	-1.545145	1.485215

O	1.087637	0.788719	2.232739
C	2.674819	-0.274365	0.429357
C	3.016461	-1.423432	-0.273488
C	3.517710	0.824566	0.534003
C	4.249357	-1.460237	-0.905519
H	2.335505	-2.262939	-0.319427
C	4.748090	0.766019	-0.102839
H	3.220199	1.696291	1.101587
C	5.107689	-0.368885	-0.821916
H	4.540067	-2.343064	-1.460205
H	5.426293	1.606804	-0.035153
H	6.069634	-0.405510	-1.318472
N	-0.159978	1.753725	-0.126896
O	0.823407	2.428189	0.035819
O	-1.272630	2.136071	-0.384222
C	-6.131938	-1.729569	1.430953
F	-7.091853	-0.872824	1.073632
F	-6.044260	-1.687705	2.768635
F	-6.566411	-2.959943	1.117534

21d-anion

C	-4.363123	-1.668080	0.653368
C	-4.244220	-0.291824	0.475140
C	-3.101861	0.221056	-0.111808
C	-2.093329	-0.649851	-0.508418
C	-2.205767	-2.015863	-0.327006
C	-3.355110	-2.533053	0.259970
H	-5.041819	0.370742	0.789822
H	-2.994655	1.288622	-0.261319
H	-1.403123	-2.670367	-0.639704
H	-3.457748	-3.600413	0.406071
S	-0.641091	0.027651	-1.302788
N	0.023825	1.045599	-0.260663
S	0.804012	0.449690	1.011800
O	0.207208	-1.102143	-1.640106
O	-1.133652	0.837103	-2.402266
O	0.105159	-0.654637	1.647988
O	1.129883	1.592345	1.846090
C	2.354794	-0.199924	0.418545
C	2.707929	-1.508518	0.702705
C	3.200509	0.637579	-0.298307
C	3.936098	-1.989878	0.261111
H	2.027440	-2.140719	1.258575
C	4.421622	0.149264	-0.738828
H	2.903749	1.657041	-0.515531
C	4.789818	-1.164060	-0.458510
H	4.221004	-3.012120	0.478515
H	5.086178	0.792082	-1.303073
H	5.743923	-1.542144	-0.805605
C	-5.613849	-2.188880	1.291836
F	-6.717437	-1.735272	0.673662
F	-5.724096	-1.804567	2.575986
F	-5.684019	-3.524479	1.282490

21b’

C	-4.929281	-1.345471	0.813438
C	-4.747995	-0.292047	-0.081687
C	-3.516980	-0.065995	-0.678603
C	-2.469274	-0.921128	-0.370416
C	-2.615118	-1.988416	0.509922
C	-3.849704	-2.188131	1.098301
H	-5.580222	0.361211	-0.315327
H	-3.374125	0.752287	-1.372011
H	-1.779627	-2.642716	0.723487
H	-3.984613	-3.013579	1.787764
S	-0.914351	-0.661699	-1.119854
N	0.008737	0.234873	0.070940
S	1.109290	-0.469533	1.239705
O	-0.189483	-1.888949	-1.227358
O	-1.028632	0.230889	-2.231066
O	0.583884	-1.786000	1.421948
O	1.129416	0.474560	2.313320
C	2.655622	-0.525724	0.429789
C	2.953580	-1.630558	-0.362063
C	3.547896	0.522267	0.604814
C	4.180769	-1.669942	-0.997014
H	2.239842	-2.436668	-0.471666
C	4.773600	0.457236	-0.039557
H	3.290688	1.365665	1.231949
C	5.104430	-0.630470	-0.847294
H	4.433826	-2.523712	-1.615232
H	5.485981	1.263068	0.090029
N	-0.094048	1.634931	0.034255
O	0.919650	2.257503	0.226823
O	-1.190140	2.083923	-0.188218
C	-6.255527	-1.578432	1.475329
H	-6.588986	-2.605912	1.317650
H	-6.170934	-1.425577	2.553913
H	-7.014209	-0.899241	1.088654
C	6.427715	-0.696497	-1.550954
H	6.962595	-1.608205	-1.277079
H	6.280429	-0.718167	-2.633109
H	7.050564	0.162077	-1.302829

21b’-anion

C	-4.372676	-1.690803	0.709489
C	-4.272452	-0.318580	0.465424
C	-3.152405	0.216851	-0.149974
C	-2.112821	-0.625614	-0.524461
C	-2.185704	-1.987662	-0.289465
C	-3.315694	-2.513357	0.326044
H	-5.084633	0.335694	0.762978
H	-3.084481	1.282584	-0.335220
H	-1.362773	-2.628236	-0.579567
H	-3.373908	-3.579579	0.515441
S	-0.685373	0.071296	-1.330019
N	-0.006923	1.098108	-0.298558
S	0.797651	0.520561	0.965218
O	0.177506	-1.043507	-1.686856
O	-1.190994	0.886026	-2.422084
O	0.113096	-0.572828	1.636239
O	1.139059	1.677419	1.775418
C	2.336068	-0.141035	0.360148
C	2.647345	-1.476561	0.562366
C	3.221834	0.702362	-0.294742
C	3.861873	-1.968641	0.105057
H	1.941517	-2.121578	1.069967
C	4.431434	0.197577	-0.751019
H	2.970600	1.745232	-0.449742
C	4.769238	-1.142294	-0.558251

H	4.109217	-3.012944	0.263575
H	5.125475	0.854469	-1.263284
C	-5.606949	-2.264207	1.347579
H	-6.384344	-2.426793	0.596001
H	-5.396642	-3.223395	1.821307
H	-6.013492	-1.583678	2.096975
C	6.075106	-1.694862	-1.058196
H	6.607819	-2.215063	-0.259610
H	5.906484	-2.417148	-1.860567
H	6.717349	-0.902913	-1.443282

21b

C	-4.854541	-1.439098	0.776301
C	-4.711320	-0.379485	-0.118468
C	-3.486420	-0.101268	-0.705607
C	-2.405992	-0.911053	-0.388039
C	-2.512222	-1.981602	0.494094
C	-3.741690	-2.233631	1.072532
H	-5.568892	0.237156	-0.359763
H	-3.372593	0.722663	-1.397795
H	-1.650291	-2.597361	0.717204
H	-3.846472	-3.062698	1.762944
S	-0.858528	-0.588988	-1.127854
N	0.016631	0.351396	0.068570
S	1.146519	-0.300852	1.233913
O	-0.080888	-1.784308	-1.224108
O	-1.000841	0.295027	-2.242433
O	0.672622	-1.634448	1.432178
O	1.145517	0.651856	2.299693
C	2.691896	-0.310136	0.407552
C	3.014502	-1.416208	-0.369409
C	3.545326	0.774128	0.564773
C	4.237638	-1.423782	-1.021272
H	2.325753	-2.246046	-0.456421
C	4.766349	0.745139	-0.092154
H	3.261963	1.613045	1.186401
C	5.106686	-0.346488	-0.883878
H	4.513289	-2.274007	-1.631832
H	5.452873	1.574730	0.016810
H	6.061188	-0.359863	-1.395657
N	-0.140137	1.747041	0.018428
O	0.847517	2.410741	0.206875
O	-1.252761	2.148760	-0.210985
C	-6.175395	-1.730288	1.425566
H	-6.473379	-2.764487	1.241899
H	-6.101405	-1.600777	2.507866
H	-6.954704	-1.068607	1.049894

21b-anion

C	-4.364070	-1.676663	0.689976
C	-4.253184	-0.309412	0.428860
C	-3.122185	0.211660	-0.181122
C	-2.084044	-0.641108	-0.531817
C	-2.168239	-2.000611	-0.280740
C	-3.307369	-2.511026	0.328207
H	-5.063755	0.353963	0.709489
H	-3.046012	1.274683	-0.378542
H	-1.345723	-2.649457	-0.552944
H	-3.374452	-3.574448	0.530863
S	-0.642010	0.035905	-1.327967
N	0.024608	1.080537	-0.305655
S	0.800665	0.524712	0.983861
O	0.223980	-1.087681	-1.647802
O	-1.126124	0.831045	-2.443400
O	0.093755	-0.543986	1.670465
O	1.148404	1.694882	1.771259
C	2.342858	-0.170130	0.416505
C	2.636541	-1.500645	0.664949
C	3.238425	0.653501	-0.254098
C	3.853422	-2.018435	0.233867
H	1.918001	-2.121265	1.184781
C	4.448246	0.129404	-0.684923
H	2.989701	1.691663	-0.440750
C	4.756007	-1.206463	-0.440731
H	4.091961	-3.057832	0.424070
H	5.152010	0.762387	-1.211459
H	5.701629	-1.612259	-0.779785
C	-5.603861	-2.241062	1.325697
H	-6.297992	-2.597072	0.559538
H	-5.362925	-3.087945	1.969451
H	-6.121348	-1.485445	1.917326

21e’

C	-4.885463	-1.276578	0.826758
C	-4.747425	-0.255232	-0.112263
C	-3.521170	-0.060889	-0.724566
C	-2.473264	-0.900791	-0.377175
C	-2.595557	-1.926463	0.550188
C	-3.821239	-2.112936	1.164069
H	-5.591325	0.374642	-0.360438
H	-3.386495	0.720568	-1.460211
H	-1.754041	-2.564594	0.785355
H	-3.956112	-2.900386	1.893440
S	-0.915875	-0.672655	-1.160782
N	-0.015010	0.313453	-0.034581
S	1.053710	-0.293360	1.206851
O	-0.186602	-1.899419	-1.183752
O	-1.056405	0.139658	-2.326119
O	0.550787	-1.604423	1.463416
O	1.054320	0.721050	2.211328
C	2.625509	-0.383893	0.421340
C	2.929454	-1.527889	-0.304165
C	3.506451	0.677240	0.571595
C	4.167614	-1.606409	-0.916340
H	2.218832	-2.339594	-0.385254
C	4.746429	0.591412	-0.037688
H	3.235371	1.546068	1.156137
C	5.063874	-0.546943	-0.777780
H	4.441468	-2.481496	-1.490414
H	5.463801	1.395109	0.061212
N	-0.132664	1.714088	-0.165344
O	0.874717	2.351599	-0.003365
O	-1.230261	2.132347	-0.426167
C	-6.161495	-1.476147	1.461093
N	-7.179883	-1.634065	1.964704
C	6.354190	-0.637444	-1.408171
N	7.382872	-0.712760	-1.909967

21e’-anion

C	-4.449054	-1.678949	0.713869

C	-4.381692	-0.305514	0.471315
C	-3.244881	0.220768	-0.115281
C	-2.195427	-0.626837	-0.446873
C	-2.256583	-1.989658	-0.207238
C	-3.393751	-2.525151	0.377288
H	-5.213129	0.333772	0.738415
H	-3.175271	1.282891	-0.315458
H	-1.420818	-2.623185	-0.472393
H	-3.465475	-3.586872	0.574918
S	-0.742543	0.079307	-1.214868
N	-0.073635	1.043241	-0.120896
S	0.732582	0.381942	1.098000
O	0.104043	-1.035546	-1.599934
O	-1.231768	0.943275	-2.271403
O	0.081927	-0.789826	1.657211
O	1.052588	1.465565	2.006518
C	2.295121	-0.176099	0.431327
C	2.600824	-1.526995	0.429453
C	3.182373	0.774116	-0.058565
C	3.824667	-1.944703	-0.070341
H	1.887282	-2.243339	0.814348
C	4.404179	0.365003	-0.561919
H	2.921996	1.825476	-0.046319
C	4.718821	-0.995633	-0.562911
H	4.087105	-2.994765	-0.080688
H	5.112336	1.085705	-0.949861
C	-5.630436	-2.229031	1.319825
N	-6.574792	-2.665803	1.804808
C	5.988440	-1.426394	-1.080730
N	7.002294	-1.771927	-1.493795

21e

C	-4.872023	-1.371905	0.734564
C	-4.746502	-0.314122	-0.164826
C	-3.513146	-0.057758	-0.738899
C	-2.446340	-0.875752	-0.397554
C	-2.556354	-1.937888	0.489138
C	-3.788606	-2.185867	1.066764
H	-5.604555	0.297448	-0.410416
H	-3.386295	0.755562	-1.440581
H	-1.699593	-2.556498	0.721909
H	-3.913922	-3.002499	1.765057
S	-0.878856	-0.569064	-1.135596
N	-0.022067	0.362227	0.059085
S	1.089943	-0.296851	1.252146
O	-0.125383	-1.779087	-1.218557
O	-1.022192	0.302538	-2.257915
O	0.602028	-1.625669	1.443655
O	1.070966	0.662419	2.310487
C	2.643565	-0.314030	0.445210
C	2.971452	-1.425266	-0.322369
C	3.496494	0.770165	0.606444
C	4.201321	-1.438149	-0.960891
H	2.282417	-2.254644	-0.411875
C	4.724222	0.735119	-0.037229
H	3.208404	1.612933	1.220720
C	5.070581	-0.361452	-0.819545
H	4.481925	-2.291883	-1.564207
H	5.411278	1.563810	0.074733
H	6.030524	-0.379284	-1.320920
N	-0.181557	1.761278	0.011524
O	0.802961	2.422140	0.215756
O	-1.292022	2.160690	-0.229886
C	-6.153276	-1.631562	1.336003
N	-7.174937	-1.837481	1.815132

21e-anion

C	-4.460529	-1.700167	0.638627
C	-4.393499	-0.321723	0.425841
C	-3.250891	0.219167	-0.135813
C	-2.195154	-0.618323	-0.473292
C	-2.256312	-1.986005	-0.262649
C	-3.398837	-2.536481	0.297036
H	-5.229760	0.310002	0.696088
H	-3.181339	1.285372	-0.312781
H	-1.415589	-2.611509	-0.531153
H	-3.470160	-3.602338	0.471314
S	-0.734177	0.107813	-1.209214
N	-0.072383	1.038087	-0.086950
S	0.732805	0.344148	1.120955
O	0.107654	-0.999989	-1.625079
O	-1.218323	1.001731	-2.244338
O	0.074008	-0.843078	1.640210
O	1.022166	1.408285	2.064683
C	2.303519	-0.183137	0.463370
C	2.620378	-1.530867	0.420677
C	3.195153	0.784098	0.016258
C	3.858793	-1.919133	-0.078691
H	1.905094	-2.262678	0.773309
C	4.427279	0.388745	-0.484085
H	2.928051	1.833524	0.059755
C	4.759117	-0.962416	-0.530333
H	4.117668	-2.970378	-0.114401
H	5.129672	1.133995	-0.836841
H	5.722845	-1.269102	-0.918714
C	-5.648132	-2.265942	1.217239
N	-6.598047	-2.716191	1.678641

21c

C	-4.913273	-1.235444	0.808487
C	-4.762125	-0.204825	-0.112551
C	-3.534256	0.008895	-0.721073
C	-2.482553	-0.832698	-0.384478
C	-2.610696	-1.873450	0.526910
C	-3.844688	-2.067316	1.127885
H	-5.875181	-1.394433	1.280645
H	-5.600758	0.432813	-0.360881
H	-3.394493	0.803354	-1.442067
H	-1.766088	-2.510352	0.755779
H	-3.972279	-2.870141	1.842459
S	-0.927123	-0.585521	-1.149215
N	-0.025747	0.386128	-0.003100
S	1.064298	-0.240075	1.216178
O	-0.185049	-1.805628	-1.193659
O	-1.055946	0.247311	-2.303668
O	0.556861	-1.553793	1.458023
O	1.060472	0.757178	2.240376
C	2.625916	-0.314782	0.424997
C	2.935103	-1.449653	-0.314999
C	3.503718	0.751089	0.572152

C	4.171204	-1.505779	-0.939665
H	2.226866	-2.263798	-0.395292
C	4.737362	0.673212	-0.056646
H	3.228682	1.613131	1.165292
C	5.065236	-0.447844	-0.811545
H	4.437504	-2.378408	-1.522122
H	5.442990	1.487486	0.045739
H	6.029857	-0.500263	-1.301489
N	-0.143648	1.783001	-0.114687
O	0.861894	2.423850	0.055798
O	-1.241817	2.206868	-0.370727

21c-anion

C	-4.453790	-1.739351	0.671126
C	-4.383277	-0.363186	0.473548
C	-3.250649	0.199269	-0.097247
C	-2.194461	-0.626064	-0.462663
C	-2.252002	-1.996555	-0.270639
C	-3.391690	-2.553871	0.298663
H	-5.339862	-2.175811	1.116136
H	-5.212607	0.270996	0.761999
H	-3.186476	1.268815	-0.258946
H	-1.411970	-2.614600	-0.559992
H	-3.446425	-3.624574	0.453132
S	-0.749172	0.112559	-1.203292
N	-0.079287	1.057015	-0.090877
S	0.734473	0.383369	1.118106
O	0.108837	-0.982871	-1.624839
O	-1.236633	1.006317	-2.239314
O	0.077633	-0.786521	1.678509
O	1.048562	1.465389	2.034932
C	2.293431	-0.174826	0.455296
C	2.606023	-1.524216	0.460852
C	3.182184	0.770102	-0.042612
C	3.835701	-1.937040	-0.040597
H	1.893203	-2.238836	0.851703
C	4.405879	0.350723	-0.544498
H	2.919309	1.821522	-0.036910
C	4.732931	-1.002470	-0.542262
H	4.090110	-2.990036	-0.039299
H	5.105882	1.079041	-0.935465
H	5.689954	-1.327897	-0.932075

21f

C	-4.823914	-1.386699	0.748913
C	-4.726074	-0.339091	-0.149723
C	-3.495429	-0.090776	-0.735338
C	-2.426176	-0.904449	-0.391296
C	-2.529603	-1.956100	0.508314
C	-3.759507	-2.202074	1.094476
H	-5.589412	0.263762	-0.392488
H	-3.375379	0.712740	-1.449205
H	-1.671290	-2.570810	0.744712
H	-3.887885	-3.009581	1.800796
S	-0.864191	-0.609734	-1.149736
N	-0.010913	0.369535	0.004606
S	1.089836	-0.243388	1.233124
O	-0.103861	-1.817060	-1.194893
O	-1.024318	0.219553	-2.301196
O	0.594321	-1.561579	1.472919
O	1.064747	0.758745	2.250791
C	2.649768	-0.297976	0.440750
C	2.982910	-1.441623	-0.275078
C	3.501856	0.792183	0.561530
C	4.217396	-1.481635	-0.903726
H	2.294914	-2.274642	-0.333232
C	4.733791	0.730023	-0.071866
H	3.210266	1.659798	1.138430
C	5.085309	-0.399298	-0.803692
H	4.501965	-2.360176	-1.468312
H	5.419750	1.563324	0.008645
H	6.048573	-0.438680	-1.297472
N	-0.168730	1.766541	-0.098218
O	0.817891	2.431838	0.078673
O	-1.278711	2.158519	-0.352483
N	-6.137499	-1.654319	1.368537
O	-6.232816	-2.621215	2.094737
O	-7.046923	-0.892524	1.115422

21f-anion

C	-4.386481	-1.676836	0.674855
C	-4.345594	-0.308433	0.454124
C	-3.217947	0.224260	-0.145758
C	-2.171795	-0.617669	-0.501959
C	-2.227929	-1.983101	-0.275852
C	-3.354020	-2.528369	0.322255
H	-5.176550	0.319253	0.743188
H	-3.153885	1.288684	-0.334420
H	-1.396291	-2.613567	-0.560257
H	-3.426575	-3.589467	0.514175
S	-0.730327	0.097395	-1.288663
N	-0.078490	1.097709	-0.224643
S	0.701152	0.487092	1.044270
O	0.127662	-1.015856	-1.652711
O	-1.241115	0.922845	-2.366734
O	0.007226	-0.636940	1.650795
O	1.004955	1.619463	1.899316
C	2.262843	-0.134358	0.453599
C	2.542872	-1.488477	0.533709
C	3.184284	0.765161	-0.068468
C	3.773774	-1.952864	0.083200
H	1.805093	-2.167468	0.941149
C	4.408509	0.293667	-0.519946
H	2.946556	1.821196	-0.119744
C	4.703338	-1.064684	-0.443355
H	4.003098	-3.009832	0.142154
H	5.133780	0.986326	-0.929046
H	5.660440	-1.429671	-0.796178
N	-5.580727	-2.248549	1.314433
O	-5.608453	-3.449021	1.500890
O	-6.479191	-1.491657	1.625071

21f'

C	-4.859850	-1.301358	0.791981
C	-4.737105	-0.270607	-0.123013
C	-3.507407	-0.076686	-0.730821
C	-2.464260	-0.924957	-0.390111
C	-2.593237	-1.961609	0.523665
C	-3.822271	-2.152629	1.131739
H	-5.580563	0.361682	-0.360734

H	-3.369410	0.711665	-1.458184
H	-1.755960	-2.606603	0.754366
H	-3.970140	-2.946167	1.849960
S	-0.902360	-0.693623	-1.167341
N	-0.006474	0.279059	-0.027451
S	1.051054	-0.343659	1.213833
O	-0.176976	-1.922107	-1.198170
O	-1.038814	0.129526	-2.324955
O	0.543196	-1.654987	1.456722
O	1.054361	0.662163	2.226294
C	2.626448	-0.433873	0.431807
C	2.934493	-1.578348	-0.290786
C	3.504116	0.629612	0.582738
C	4.175284	-1.656839	-0.900370
H	2.226744	-2.392338	-0.372916
C	4.745510	0.548403	-0.026157
H	3.231728	1.498355	1.166448
C	5.045699	-0.590660	-0.752682
H	4.458227	-2.528373	-1.473216
H	5.463556	1.350361	0.067508
N	-0.119646	1.682795	-0.145453
O	0.889083	2.314884	0.026256
O	-1.214960	2.105643	-0.406505
N	-6.171723	-1.508162	1.438373
O	-6.289523	-2.455839	2.186171
O	-7.056899	-0.718908	1.184192
N	6.371444	-0.676230	-1.398751
O	6.649651	-1.702183	-1.982881
O	7.107253	0.283680	-1.307491

21f'-anion

C	-4.431734	-1.647943	0.684323
C	-4.386538	-0.281953	0.451348
C	-3.247937	0.244650	-0.133157
C	-2.196053	-0.601724	-0.460698
C	-2.255741	-1.964910	-0.222000
C	-3.393445	-2.503634	0.359668
H	-5.222682	0.348476	0.718594
H	-3.180019	1.306661	-0.333419
H	-1.418715	-2.597781	-0.483819
H	-3.470406	-3.562736	0.560549
S	-0.738148	0.106788	-1.218680
N	-0.069546	1.054402	-0.110830
S	0.730416	0.373766	1.100859
O	0.104573	-1.007718	-1.611729
O	-1.222044	0.983770	-2.266021
O	0.075361	-0.803760	1.641464
O	1.055053	1.442285	2.024654
C	2.292603	-0.181035	0.428214
C	2.595585	-1.532609	0.412744
C	3.182353	0.772992	-0.049796
C	3.820421	-1.948820	-0.087810
H	1.880770	-2.252491	0.788077
C	4.405246	0.368403	-0.555697
H	2.924661	1.824622	-0.027375
C	4.696619	-0.987310	-0.560066
H	4.087510	-2.995891	-0.111268
H	5.118803	1.085679	-0.935691
N	-5.638578	-2.213092	1.306807
O	-5.674766	-3.413038	1.494162
O	-6.537503	-1.451148	1.602943
N	5.997238	-1.423177	-1.091465
O	6.252695	-2.610886	-1.071071
O	6.749738	-0.573076	-1.524045

21a

C	-4.807147	-1.407956	0.780751
C	-4.687083	-0.294247	-0.055648
C	-3.475923	-0.036042	-0.674568
C	-2.403499	-0.889102	-0.454431
C	-2.511649	-2.008341	0.372003
C	-3.715129	-2.263473	0.986514
H	-5.522019	0.368105	-0.232101
H	-3.371648	0.820035	-1.328306
H	-1.663709	-2.663698	0.521729
H	-3.838419	-3.124243	1.631252
S	-0.886568	-0.564403	-1.233906
N	0.029654	0.405180	-0.087563
S	1.079225	-0.237175	1.151473
O	-0.099178	-1.753572	-1.333387
O	-1.061355	0.306359	-2.354815
O	0.561823	-1.552704	1.363196
O	1.055786	0.745369	2.190148
C	2.662071	-0.310075	0.402701
C	2.988522	-1.441956	-0.334275
C	3.537996	0.752973	0.577799
C	4.240613	-1.498480	-0.926245
H	2.280338	-2.253610	-0.436423
C	4.787656	0.675220	-0.018891
H	3.249866	1.612585	1.168340
C	5.133149	-0.443056	-0.770033
H	4.519897	-2.368962	-1.505876
H	5.492074	1.487422	0.105592
H	6.110290	-0.495433	-1.234504
N	-0.108936	1.800937	-0.155455
O	0.891231	2.449348	0.024001
O	-1.214264	2.221061	-0.386826
O	-5.933511	-1.740093	1.425795
C	-7.071113	-0.894540	1.271639
H	-7.391002	-0.862592	0.228619
H	-7.853783	-1.338636	1.881018
H	-6.853501	0.113957	1.628339

21a-anion

C	-4.466625	-1.605809	0.514996
C	-4.395067	-0.233584	0.270945
C	-3.236507	0.300695	-0.278033
C	-2.161607	-0.520293	-0.577648
C	-2.230128	-1.886409	-0.334307
C	-3.379561	-2.428804	0.207956
H	-5.225282	0.419818	0.498663
H	-3.177735	1.365272	-0.472254
H	-1.382124	-2.516939	-0.568117
H	-3.455965	-3.491126	0.404770
S	-0.693554	0.196983	-1.274259
N	-0.023735	1.124492	-0.143812
S	0.743630	0.432779	1.082661
O	0.156147	-0.910665	-1.682761
O	-1.133907	1.110609	-2.314772
O	0.037974	-0.708495	1.643605

O	1.081996	1.509983	1.997355
C	2.292869	-0.188977	0.453771
C	2.566302	-1.545870	0.501848
C	3.213945	0.714254	-0.062652
C	3.787758	-2.009180	0.024554
H	1.829219	-2.228406	0.904768
C	4.428955	0.244861	-0.540674
H	2.981955	1.772506	-0.090893
C	4.716249	-1.116652	-0.496360
H	4.010641	-3.068683	0.058297
H	5.152835	0.941288	-0.946008
H	5.666105	-1.480582	-0.869396
O	-5.545604	-2.223957	1.044163
C	-6.672534	-1.420981	1.370264
H	-7.074409	-0.931413	0.480349
H	-7.416015	-2.101375	1.778451
H	-6.413499	-0.670523	2.120465

21a'

C	-4.922368	-1.303633	0.845200
C	-4.773286	-0.276378	-0.090664
C	-3.550054	-0.096121	-0.714327
C	-2.497418	-0.944321	-0.404413
C	-2.633424	-1.977713	0.523314
C	-3.846654	-2.151886	1.147033
H	-5.595180	0.379449	-0.337820
H	-3.419375	0.696149	-1.439821
H	-1.797487	-2.628618	0.745177
H	-3.991416	-2.942855	1.871748
S	-0.963945	-0.716397	-1.188085
N	-0.014119	0.232758	-0.056819
S	1.099670	-0.417745	1.133967
O	-0.237392	-1.945926	-1.256048
O	-1.096127	0.128140	-2.334939
O	0.583818	-1.731232	1.363533
O	1.109666	0.564255	2.174186
C	2.638864	-0.487333	0.329974
C	2.934764	-1.593785	-0.467649
C	3.540057	0.553436	0.499509
C	4.153396	-1.644738	-1.103157
H	2.217646	-2.396535	-0.581048
C	4.769537	0.497448	-0.134894
H	3.286792	1.400199	1.124003
C	5.077085	-0.601759	-0.941551
H	4.420464	-2.488219	-1.726962
H	5.473933	1.304378	0.004032
N	-0.118034	1.627202	-0.148819
O	0.896891	2.257585	0.015134
O	-1.214837	2.070053	-0.383634
O	-6.062402	-1.554521	1.503433
C	-7.193582	-0.732472	1.224604
H	-7.484206	-0.817528	0.175815
H	-7.993522	-1.105515	1.858497
H	-6.984132	0.309992	1.471418
O	6.236770	-0.745519	-1.597758
C	7.225601	0.269946	-1.443245
H	7.527566	0.359328	-0.397978
H	8.073967	-0.049331	-2.042769
H	6.856535	1.229228	-1.810804

21a'-anion

C	-4.461398	-1.640424	0.489130
C	-4.414869	-0.260663	0.284112
C	-3.270372	0.308583	-0.259199
C	-2.184864	-0.485034	-0.592233
C	-2.228039	-1.858229	-0.386732
C	-3.363219	-2.435501	0.150137
H	-5.253893	0.371575	0.537824
H	-3.230847	1.379295	-0.422037
H	-1.371456	-2.466905	-0.646271
H	-3.419736	-3.504091	0.317641
S	-0.737379	0.276083	-1.287329
N	-0.087998	1.211731	-0.154333
S	0.696952	0.532063	1.071514
O	0.136136	-0.808391	-1.709211
O	-1.206054	1.184476	-2.320705
O	0.006650	-0.619732	1.631862
O	1.009594	1.618990	1.984532
C	2.249277	-0.066210	0.449061
C	2.518112	-1.428890	0.421598
C	3.189885	0.843265	-0.005854
C	3.734286	-1.877865	-0.057336
H	1.773424	-2.130613	0.774600
C	4.413564	0.402821	-0.493895
H	2.974617	1.905252	0.015867
C	4.687382	-0.965618	-0.517804
H	3.966472	-2.935488	-0.084883
H	5.136015	1.125230	-0.846955
O	-5.525706	-2.291996	1.008574
C	-6.667461	-1.519290	1.355107
H	-7.078162	-1.013572	0.478316
H	-7.398260	-2.223959	1.744705
H	-6.423056	-0.784509	2.125467
O	5.846150	-1.497020	-0.967161
C	6.840059	-0.601215	-1.446783
H	7.162266	0.083375	-0.658879
H	7.679464	-1.220409	-1.753858
H	6.472506	-0.032487	-2.303896

9e

C	-1.556039	-0.536557	-0.130508
C	-0.203088	-0.510946	0.020618
C	0.145107	0.851433	0.232791
N	-1.928294	0.765315	-0.004698
H	-2.262356	-1.330933	-0.306998
H	1.125238	1.275498	0.393036
N	-3.250589	1.240438	-0.081576
O	-3.406179	2.417411	0.072305
O	-4.083236	0.397074	-0.295593
N	-0.916995	1.619623	0.215837
C	0.710298	-1.677372	-0.021761
F	1.399415	-1.819595	1.119816
F	1.621416	-1.572615	-0.999871
F	0.040252	-2.816332	-0.225017

9e-anion

C	-1.568383	-0.486179	-0.147227
C	-0.181680	-0.509512	0.025202
C	0.150627	0.831170	0.246789
N	-2.015348	0.765965	-0.035291

H	-2.239029	-1.311384	-0.342690
H	1.123535	1.267354	0.428957
N	-0.952894	1.579729	0.208466
C	0.715172	-1.665266	-0.017614
F	1.461002	-1.815479	1.103304
F	1.623905	-1.619239	-1.023436
F	0.049382	-2.824866	-0.179722

9d

C	-1.566598	-0.546127	-0.000024
C	-0.206946	-0.503661	-0.000005
C	0.155549	0.870158	-0.000006
N	-1.932533	0.769932	-0.000197
H	-2.278306	-1.354472	0.000062
H	1.146332	1.298654	0.000052
N	-3.252100	1.240641	-0.000269
O	-3.401360	2.430300	-0.000413
O	-4.100858	0.383733	-0.000169
N	-0.909684	1.636008	-0.000350
Cl	0.865971	-1.842555	0.000215

9d-anion

C	-1.542306	-0.468976	0.000066
C	-0.152064	-0.499497	0.000080
C	0.237304	0.835800	-0.000259
N	-1.950867	0.808541	-0.000458
H	-2.238703	-1.295603	0.000045
H	1.234490	1.252659	-0.000297
N	-0.858189	1.610807	0.000680
Cl	0.878070	-1.903287	0.000216

9f

C	-1.561725	-0.553905	-0.000042
C	-0.193066	-0.526314	0.000002
C	0.168722	0.854370	-0.000082
N	-1.918571	0.753293	-0.000179
H	-2.277318	-1.359953	0.000001
H	1.159965	1.281119	-0.000065
N	-3.246724	1.230561	-0.000267
O	-3.385882	2.418629	-0.000347
O	-4.093077	0.374828	-0.000223
N	-0.892554	1.619954	-0.000207
C	0.677460	-1.648452	0.000130
N	1.393548	-2.544736	0.000239

9f-anion

C	-1.532294	-0.481972	0.000046
C	-0.126390	-0.530527	0.000000
C	0.247074	0.826006	0.000077
N	-1.938514	0.781176	0.000100
H	-2.229831	-1.307751	0.000109
H	1.242941	1.246300	0.000073
N	-0.838142	1.590473	-0.000319
C	0.707170	-1.667146	0.000013
N	1.387032	-2.599337	0.000039

9a

C	-1.555570	-0.540889	-0.121309
C	-0.201312	-0.538993	0.017709
C	0.133993	0.836951	0.217839
N	-1.936945	0.773350	-0.000836
H	-2.273393	-1.326084	-0.288164
H	1.116187	1.262373	0.367231
N	-3.240280	1.248651	-0.073119
O	-3.402925	2.431497	0.069780
O	-4.088132	0.410001	-0.275018
N	-0.917069	1.622137	0.206357
C	0.734161	-1.699817	-0.023342
H	1.254344	-1.810361	0.929845
H	0.194136	-2.623075	-0.229808
H	1.489919	-1.560322	-0.798118

9a-anion

C	-1.539873	-0.451206	0.009080
C	-0.142994	-0.517588	0.002769
C	0.219614	0.831839	-0.005803
N	-1.960573	0.826441	0.004100
H	-2.253593	-1.266200	0.018398
H	1.216058	1.257270	-0.010877
N	-0.868204	1.624855	-0.005119
C	0.748610	-1.725016	-0.003232
H	0.516438	-2.409762	0.816438
H	0.664747	-2.295800	-0.932264
H	1.795153	-1.431582	0.102966

9b

C	-1.554320	-0.538344	-0.000014
C	-0.194629	-0.508657	0.000036
C	0.148863	0.872693	0.000011
N	-1.934532	0.777295	-0.000246
H	-2.276327	-1.337107	0.000066
H	0.471596	-1.354795	0.000185
H	1.132673	1.317139	0.000078
N	-3.250969	1.239447	-0.000411
O	-3.411820	2.429618	-0.000383
O	-4.100435	0.380628	-0.000074
N	-0.911686	1.647861	-0.000370

9b-anion

C	-1.535158	-0.461237	0.000018
C	-0.138969	-0.517684	0.000034
C	0.228036	0.830974	-0.000016
N	-1.955463	0.813577	0.000040
H	-2.248078	-1.275459	0.000050
H	0.499037	-1.388161	0.000064
H	1.218997	1.265900	-0.000042
N	-0.861718	1.615698	-0.000090

9c

C	-1.569856	-0.550545	-0.000007
C	-0.213611	-0.491835	0.000017
C	0.158993	0.874896	-0.000004
N	-1.932787	0.769794	-0.000181
H	-2.277030	-1.362168	0.000081
H	1.149511	1.303329	0.000043
N	-3.249618	1.240302	-0.000274
O	-3.399872	2.430456	-0.000441
O	-4.099935	0.384138	-0.000187
N	-0.911893	1.635934	-0.000336
F	0.622028	-1.521853	0.000173

9c-anion

C	-1.545082	-0.472258	0.000008
C	-0.159847	-0.488937	0.000044
C	0.241311	0.837417	-0.000045
N	-1.951124	0.812083	0.000026
H	-2.241676	-1.298119	0.000031
H	1.238568	1.253028	-0.000077
N	-0.861663	1.611340	-0.000023
F	0.643713	-1.584277	0.000100

9g

C	-1.567121	-0.553983	0.000023
C	-0.202481	-0.504285	0.000071
C	0.174639	0.861423	0.000023
N	-1.917960	0.751408	-0.000209
H	-2.277852	-1.364809	0.000086
H	1.165262	1.288797	0.000068
N	-3.251003	1.232803	-0.000339
O	-3.383578	2.420043	-0.000458
O	-4.095664	0.377662	-0.000144
N	-0.892386	1.621391	-0.000341
N	0.673163	-1.637249	0.000247
O	1.868333	-1.414178	-0.000071
O	0.170045	-2.743676	0.000045

9g-anion

C	-1.545293	-0.479202	0.000015
C	-0.142857	-0.511646	0.000026
C	0.247766	0.835985	-0.000046
N	-1.944743	0.781005	0.000010
H	-2.240770	-1.305554	0.000042
H	1.245308	1.249876	-0.000073
N	-0.834338	1.595785	-0.000005
N	0.688156	-1.640540	0.000069
O	1.906868	-1.476222	0.000052
O	0.171570	-2.756308	0.000092

32b

C	-1.392431	0.336717	0.555597
O	-0.095770	0.860185	0.153828
N	0.888482	-0.070351	0.010417
O	0.651459	-1.212974	0.298779
O	1.922301	0.385583	-0.395947
C	-2.122639	-0.295919	-0.603193
H	-3.115650	-0.595988	-0.264167
H	-2.239094	0.419245	-1.418093
H	-1.606043	-1.182266	-0.970817
H	-1.251950	-0.339882	1.395771
H	-1.892828	1.238796	0.903031

32b-anion

C	-1.311514	0.387449	0.527812
O	-0.073089	0.779638	0.139951
C	-2.134461	-0.284356	-0.586564
H	-3.127029	-0.597486	-0.240930
H	-2.263301	0.407657	-1.424857
H	-1.606054	-1.167195	-0.960146
H	-1.301208	-0.340591	1.378981
H	-1.945684	1.227647	0.910753

32a

C	0.033055	-1.054296	0.917482
O	0.831176	-1.021599	-0.298918
N	1.813759	-0.080563	-0.328623
O	2.023073	0.570298	0.660639
O	2.386087	-0.018367	-1.382956
C	-0.946960	0.097738	1.005463
H	-0.421357	1.014975	1.278964
H	-1.620247	-0.141241	1.836555
H	-0.472686	-2.012700	0.812376
H	0.700474	-1.100991	1.776002
C	-1.773381	0.337842	-0.261821
H	-1.092399	0.654649	-1.059996
C	-2.765097	1.470058	-0.010300
H	-3.330287	1.704792	-0.914875
H	-3.478047	1.184879	0.769267
H	-2.254955	2.379490	0.316292
C	-2.501339	-0.922223	-0.723477
H	-1.810234	-1.709563	-1.031628
H	-3.130709	-1.318465	0.079846
H	-3.146604	-0.699230	-1.576179

32a-anion

C	0.093049	-1.142563	0.685653
O	0.872763	-1.041601	-0.418363
C	-0.898774	0.023539	0.883344
H	-0.319885	0.938953	1.065313
H	-1.506534	-0.156539	1.783970
H	-0.519442	-2.079947	0.713919
H	0.676490	-1.192736	1.641192
C	-1.829254	0.270355	-0.304761
H	-1.194453	0.471921	-1.173305
C	-2.717925	1.487564	-0.056262
H	-3.342944	1.713728	-0.924884
H	-3.384045	1.309996	0.794817
H	-2.121652	2.376055	0.169200
C	-2.687042	-0.952523	-0.621641
H	-2.073579	-1.808814	-0.908030
H	-3.284205	-1.241844	0.250124
H	-3.377637	-0.744034	-1.443627

33a

C	-1.334624	0.327458	0.519554
O	-0.215419	0.997724	-0.093563
H	-0.986812	-0.367568	1.280340
H	-1.895236	1.142134	0.970196
H	-1.929828	-0.179280	-0.237038
N	0.655002	0.162135	-0.725998
O	0.428076	-1.018036	-0.708389
O	1.577893	0.730098	-1.242185

33a-anion

C	-1.301336	0.385634	0.544891
O	-0.090532	0.817920	0.109294
H	-1.259615	-0.354445	1.382314
H	-1.973016	1.191211	0.934021
H	-1.922279	-0.126371	-0.232391

16b

N	-3.587898	-0.599790	0.628550
C	-2.208183	-0.914861	0.469541
C	-1.461662	0.173592	-0.135511
C	-2.070121	1.297993	-0.558054
C	-3.478735	1.499784	-0.433093
C	-4.207490	0.535411	0.150380
H	-3.956189	2.403222	-0.784424
O	-1.751247	-1.987663	0.783832
H	-0.398201	0.008672	-0.242910
H	-5.275135	0.591388	0.305403
C	-1.270918	2.403141	-1.193385
F	-1.414515	3.548546	-0.514075
F	-1.684679	2.638686	-2.445216
F	0.032454	2.133285	-1.248760
N	-4.435697	-1.568061	1.248407
O	-5.513635	-1.727043	0.734696
O	-4.001003	-2.111750	2.221877

16b-anion

N	-3.600666	-0.644599	0.595693
C	-2.241179	-0.827774	0.521987
C	-1.450710	0.214448	-0.096829
C	-2.057904	1.335506	-0.586772
C	-3.448701	1.500065	-0.502162
C	-4.143582	0.468413	0.100492
H	-3.950633	2.378424	-0.884059
O	-1.705319	-1.864419	0.977160
H	-0.378912	0.076847	-0.156160
H	-5.225011	0.546396	0.191786
C	-1.254912	2.427329	-1.223427
F	-1.383216	3.594662	-0.564842
F	-1.649378	2.670803	-2.487193
F	0.055998	2.162358	-1.277161

16c

N	-3.597806	-0.606602	0.632464
C	-2.228986	-0.935457	0.426112
C	-1.485398	0.160302	-0.171499
C	-2.113871	1.287651	-0.544412
C	-3.506234	1.517150	-0.388808
C	-4.220592	0.541667	0.198052
H	-3.977789	2.431984	-0.709581
O	-1.776003	-2.020160	0.697470
H	-0.425911	0.005814	-0.319156
H	-5.282834	0.603927	0.385668
N	-4.434815	-1.580712	1.266128
O	-5.522310	-1.734043	0.774373
O	-3.976281	-2.128557	2.225227
N	-1.302576	2.362091	-1.169602
O	-0.103771	2.205849	-1.230202
O	-1.899950	3.332503	-1.580673

16c-anion

N	-3.601582	-0.653508	0.604933
C	-2.246530	-0.850889	0.479559
C	-1.467587	0.197632	-0.142730
C	-2.099985	1.325262	-0.574106
C	-3.475169	1.520236	-0.445358
C	-4.151817	0.473943	0.158961
H	-3.974309	2.412596	-0.786917
O	-1.706525	-1.900944	0.890462
H	-0.400755	0.067879	-0.253996
H	-5.227624	0.560005	0.292546
N	-1.289860	2.391011	-1.206966
O	-0.088533	2.231995	-1.291857
O	-1.871358	3.378322	-1.612243

16a

N	-3.599734	-0.610981	0.644420
C	-2.228987	-0.943336	0.422663
C	-1.495244	0.147275	-0.180560
C	-2.091270	1.299368	-0.560367
C	-3.491336	1.509618	-0.373302
C	-4.219692	0.544886	0.213306
H	-3.971587	2.425587	-0.683357
O	-1.788744	-2.039158	0.693017
H	-0.440383	-0.035326	-0.336096
H	-1.499482	2.079694	-1.023908
H	-5.280094	0.609330	0.409825
N	-4.437098	-1.580053	1.265285
O	-5.530238	-1.725860	0.776186
O	-3.987250	-2.145276	2.221105

16a-anion

N	-3.602188	-0.649880	0.617748
C	-2.250304	-0.852665	0.469271
C	-1.478782	0.187628	-0.168258
C	-2.075035	1.340510	-0.607794
C	-3.457619	1.517549	-0.439916
C	-4.145743	0.486696	0.174609
H	-3.970016	2.410820	-0.770273
O	-1.715337	-1.914347	0.880158
H	-0.413527	0.025305	-0.286217
H	-1.478773	2.112882	-1.084442
H	-5.220319	0.581403	0.324058

17c

C	-0.546666	-0.296220	-1.682466
N	-1.543253	-0.959163	-1.099839
C	-1.574874	-0.927315	0.195335
C	0.345091	0.418700	0.234714
N	0.404538	0.396044	-1.059996
N	-0.637665	-0.242158	0.896875
N	-0.691412	-0.215089	2.435718
O	-1.407731	0.621032	2.855405
O	-0.008389	-1.037390	2.931713
Cl	1.472192	1.272738	1.147373
Cl	-2.768518	-1.738203	1.062019
Cl	-0.487748	-0.334679	-3.367788

17c-anion

C	-0.555694	-0.288596	-1.674536
N	-1.403521	-1.117824	-1.094501
C	-1.384542	-1.046925	0.223657
C	0.171849	0.516800	0.245720
N	0.261385	0.554520	-1.070771
N	-0.626130	-0.255480	0.960046
Cl	1.201766	1.582135	1.121021
Cl	-2.456480	-2.094264	1.069588
Cl	-0.506837	-0.311436	-3.395019

17c

C	-0.538066	-0.306657	-1.678463
N	-1.484370	-1.039188	-1.113818
C	-1.513961	-0.980158	0.172800
C	0.266915	0.487857	0.207221
N	0.349574	0.469354	-1.078772
N	-0.651508	-0.228044	0.895757
N	-0.689269	-0.218009	2.492014
O	-0.620736	0.866191	2.925180
O	-0.775628	-1.298210	2.932507
F	-0.475050	-0.353656	-2.958254
F	-2.386789	-1.631996	0.841321
F	1.075633	1.189657	0.904968

17c-anion

C	-0.554415	-0.289866	-1.660665
N	-1.402102	-1.119414	-1.095330
C	-1.375771	-1.038122	0.215893
C	0.163470	0.508084	0.237812
N	0.263716	0.552965	-1.071633
N	-0.627311	-0.254246	0.959552
F	-0.519060	-0.306232	-2.967764
F	-2.190977	-1.833402	0.858097
F	0.945428	1.318006	0.902642

17a

C	-0.552580	-0.291604	-1.678468
N	-1.404295	-1.126213	-1.078625
C	-1.435600	-1.097833	0.225000
C	0.223277	0.568011	0.248926
N	0.271824	0.552983	-1.054395
H	-2.095749	-1.736523	0.800003
H	0.850418	1.222920	0.842313
N	-0.630564	-0.250239	0.897354
N	-0.699923	-0.204260	2.399312
O	0.009116	0.606670	2.890978
O	-1.455426	-0.983042	2.873568
H	-0.526633	-0.301617	-2.761399

17a-anion

C	-0.547113	-0.296702	-1.690303
N	-1.403179	-1.116475	-1.090153
C	-1.407893	-1.047678	0.236714
C	0.177512	0.534596	0.259713
N	0.266308	0.548133	-1.065937
H	-2.092438	-1.699733	0.768646
H	0.823057	1.209910	0.810924
N	-0.640616	-0.241297	0.962842
H	-0.506874	-0.320779	-2.773855

23b

C	-2.051556	-2.012385	-0.116421
C	-0.670547	-2.099210	-0.106698
C	0.106118	-0.954452	-0.098482
C	-0.537259	0.274782	-0.102392
C	-1.923271	0.314510	-0.113707
C	-2.728439	-0.812979	-0.119701
C	-0.129237	-3.483786	-0.109124
H	1.186293	-1.019849	-0.089919
H	0.029144	1.195415	-0.096985
H	-3.808305	-0.742681	-0.127347
S	-2.824919	-3.594122	-0.120826
O	-3.477586	-3.869159	1.118106
O	-3.464840	-3.873048	-1.365824
O	1.015536	-3.807535	-0.103716
N	-1.251045	-4.358589	-0.114858
N	-1.196847	-5.746459	-0.154954
O	-2.276373	-6.291547	-0.130968
O	-0.116197	-6.262294	-0.209276
N	-2.587381	1.634313	-0.119983
O	-1.880436	2.618131	-0.122209
O	-3.799397	1.651988	-0.122786

23b-anion

C	-1.990073	-2.014322	-0.122551
C	-0.611361	-2.056814	-0.105398
C	0.132438	-0.889711	-0.091189
C	-0.546421	0.321841	-0.095080
C	-1.935671	0.320377	-0.112475
C	-2.703396	-0.836980	-0.126288
C	-0.100904	-3.485066	-0.107430
H	1.214430	-0.921166	-0.077816
H	-0.010410	1.260404	-0.084819
H	-3.784327	-0.799937	-0.140174
S	-2.555108	-3.706257	-0.140466
O	-3.298542	-3.987247	1.074946
O	-3.268779	-3.970114	-1.377507
O	1.098694	-3.731564	-0.092579
N	-1.098149	-4.395053	-0.127652
N	-2.635331	1.615055	-0.117421
O	-1.963274	2.626595	-0.116504
O	-3.849969	1.603751	-0.122319

26a

C	-2.017456	-2.075645	-0.122879
C	-0.640056	-2.148566	-0.088911
C	0.082872	-0.960803	-0.073231
C	-0.599670	0.246307	-0.093944
C	-1.994611	0.289663	-0.129425
C	-2.727920	-0.884149	-0.143824
C	-0.015395	-3.530939	-0.067339
H	1.165853	-0.981229	-0.045466
H	-0.038809	1.172595	-0.083037
H	-2.506871	1.242909	-0.145942
H	-3.810781	-0.875184	-0.170821
S	-2.777208	-3.649224	-0.136680
O	-3.474149	-3.926396	1.086910
O	-3.431624	-3.924881	-1.383724
N	-1.225290	-4.403183	-0.111056
N	-1.181956	-5.755585	-0.100413
O	-2.253877	-6.333259	-0.126849
O	-0.091655	-6.287017	-0.068160
C	0.851828	-3.739082	-1.307460
H	1.322462	-4.720173	-1.292332
H	0.254448	-3.632285	-2.214244
H	1.635408	-2.980245	-1.311685
C	0.760103	-3.735137	1.232557
H	0.101781	-3.610208	2.093562
H	1.218356	-4.721752	1.262217

H	1.550931	-2.985501	1.283550

26a-anion

C	-2.001409	-2.046536	-0.145358
C	-0.620553	-2.081686	-0.170510
C	0.087749	-0.885132	-0.163763
C	-0.616841	0.315154	-0.152183
C	-2.011972	0.328242	-0.133050
C	-2.724134	-0.865977	-0.120171
C	-0.072104	-3.508159	-0.179651
H	1.172319	-0.880747	-0.175802
H	-0.075181	1.253625	-0.160593
H	-2.541740	1.273113	-0.127408
H	-3.807974	-0.870532	-0.095865
S	-2.565384	-3.736749	-0.079244
O	-3.040740	-3.950439	1.299073
O	-3.622472	-4.003757	-1.060470
N	-1.202838	-4.406665	-0.465422
C	0.976441	-3.676803	-1.277942
H	1.314301	-4.715464	-1.304588
H	0.553879	-3.424724	-2.252588
H	1.844440	-3.038847	-1.093834
C	0.563103	-3.810605	1.186407
H	-0.180161	-3.708650	1.979924
H	0.939695	-4.836244	1.193888
H	1.395613	-3.133475	1.395951

25b

C	-2.070708	-1.991007	-0.112363
C	-0.693382	-2.065283	-0.122166
N	0.100596	-1.008468	-0.125759
C	-0.505786	0.177620	-0.120500
C	-1.893130	0.342416	-0.112020
C	-2.718392	-0.768527	-0.107051
C	-0.132623	-3.450666	-0.127822
H	0.140364	1.047498	-0.122889
H	-2.312574	1.339297	-0.108832
H	-3.797934	-0.683117	-0.099655
S	-2.831251	-3.572357	-0.104144
O	-3.475021	-3.849588	1.139668
O	-3.477787	-3.863915	-1.343651
O	1.011997	-3.767570	-0.144441
N	-1.253132	-4.330604	-0.104360
N	-1.192774	-5.716803	-0.141722
O	-2.268978	-6.268821	-0.110219
O	-0.109598	-6.228434	-0.199396

25b-anion

C	-2.077698	-1.962720	-0.111388
C	-0.700790	-2.037503	-0.120175
N	0.098209	-0.980410	-0.125461
C	-0.512954	0.208724	-0.121865
C	-1.896711	0.374673	-0.113062
C	-2.721980	-0.742769	-0.107477
C	-0.218921	-3.482347	-0.122412
H	0.132364	1.080271	-0.125986
H	-2.313746	1.373004	-0.110420
H	-3.801793	-0.654723	-0.100283
S	-2.682081	-3.632770	-0.105969
O	-3.410015	-3.889530	1.125328
O	-3.425996	-3.892347	-1.327048
O	0.970237	-3.769512	-0.129775
N	-1.245113	-4.361889	-0.114474

25a

C	-1.967002	-1.967825	-0.112840
C	-0.597831	-2.109156	-0.120114
C	0.170728	-0.953648	-0.123742
C	-0.512394	0.251025	-0.121901
C	-1.907977	0.260463	-0.115632
N	-2.652097	-0.851199	-0.109891
C	-0.104938	-3.504530	-0.121896
H	0.023596	1.190302	-0.124815
H	-2.449591	1.198479	-0.114570
S	-2.812469	-3.533116	-0.100876
O	-3.464892	-3.777403	1.145182
O	-3.469014	-3.799059	-1.340839
O	1.027115	-3.871170	-0.133095
N	-1.258753	-4.345867	-0.100383
N	-1.249422	-5.732250	-0.147589
O	-2.345566	-6.243838	-0.106448
O	-0.187684	-6.285259	-0.222969
H	1.252609	-1.002240	-0.127801

25a-anion

C	-1.998548	-1.934856	-0.111521
C	-0.632691	-2.098906	-0.121020
C	0.160104	-0.964563	-0.126278
C	-0.498954	0.259013	-0.121893
C	-1.891909	0.296436	-0.112456
N	-2.664057	-0.799148	-0.107031
C	-0.248867	-3.559086	-0.123876
H	0.054348	1.188882	-0.125751
H	-2.410331	1.248434	-0.109077
S	-2.717149	-3.580787	-0.106345
O	-3.457042	-3.793992	1.126327
O	-3.475776	-3.793217	-1.327737
O	0.924750	-3.913363	-0.131802
N	-1.319733	-4.385509	-0.116690
H	1.241623	-1.031572	-0.133731

23a

C	-2.028289	-1.997330	-0.112879
C	-0.647853	-2.104885	-0.121841
C	0.135557	-0.963037	-0.124982
C	-0.504502	0.269885	-0.120361
C	-1.894952	0.357553	-0.112469
C	-2.687933	-0.785948	-0.107925
C	-0.122624	-3.488742	-0.126145
H	1.215370	-1.041680	-0.130595
H	0.085716	1.177193	-0.122536
H	-2.371038	1.329746	-0.109313
H	-3.768760	-0.724284	-0.100953
S	-2.818522	-3.565208	-0.103881
O	-3.470363	-3.834410	1.139081
O	-3.474376	-3.850305	-1.341623
O	1.017385	-3.833888	-0.139723
N	-1.256435	-4.356187	-0.104918
N	-1.220480	-5.739803	-0.143139
O	-2.305726	-6.275470	-0.107501

O	-0.146916	-6.273210	-0.206212

23a-anion

C	-1.978988	-2.003461	-0.115614
C	-0.601011	-2.061758	-0.116023
C	0.146006	-0.895880	-0.115774
C	-0.534555	0.318627	-0.115108
C	-1.928729	0.359893	-0.114700
C	-2.679477	-0.813713	-0.114974
C	-0.104253	-3.491331	-0.116845
H	1.228640	-0.936026	-0.116117
H	0.023702	1.246855	-0.114909
H	-2.435425	1.317208	-0.114171
H	-3.762443	-0.787503	-0.114680
S	-2.557546	-3.686410	-0.116416
O	-3.292469	-3.960876	1.109108
O	-3.292876	-3.959460	-1.342015
O	1.093746	-3.756650	-0.117289
N	-1.108674	-4.396774	-0.117041

36

C	-2.410317	-0.704079	-0.242077
H	-2.506859	-0.725987	-1.327785
H	-3.245138	-0.174191	0.207671
H	-2.377094	-1.733088	0.120145
C	-1.146219	-0.021178	0.135522
O	-0.968630	0.901345	0.863294
O	-0.071519	-0.639960	-0.511719
N	1.159189	-0.019254	-0.160706
O	1.708046	-0.499999	0.777804
O	1.487506	0.873616	-0.873485

36-anion

C	-2.421235	-0.726587	-0.242077
H	-2.574812	-0.715659	-1.324758
H	-3.281142	-0.254492	0.232130
H	-2.355040	-1.770458	0.067886
C	-1.117326	0.018543	0.077187
O	-1.222691	1.185672	0.521213
O	-0.052403	-0.600449	-0.157691

11

C	-4.689972	-0.356723	0.006714
C	-3.328751	-0.574436	0.003644
C	-2.514330	0.557055	-0.000188
C	-3.054638	1.842914	-0.000913
C	-4.427553	2.077001	0.002221
C	-5.223229	0.946613	0.006027
H	-5.367925	-1.200370	0.009758
H	-2.899974	-1.568305	0.004189
H	-4.847384	3.070999	0.001828
H	-6.298867	1.071160	0.008623
N	-1.918595	2.624770	-0.004987
N	-0.784956	1.869273	-0.006718
N	-1.128856	0.651671	-0.003698
N	-1.820354	4.013030	-0.007492
O	-2.876832	4.595655	-0.005665
O	-0.715343	4.481473	-0.011072

11-anion

C	-4.697757	-0.357054	0.006812
C	-3.345471	-0.611896	0.003769
C	-2.477007	0.496342	-0.000167
C	-2.979059	1.810906	-0.000951
C	-4.364758	2.059742	0.002144
C	-5.204152	0.969558	0.005971
H	-5.399196	-1.182984	0.009914
H	-2.963257	-1.626432	0.004415
H	-4.753351	3.071856	0.001447
H	-6.277285	1.119237	0.008350
N	-1.908767	2.649798	-0.005125
N	-0.832222	1.878582	-0.006499
N	-1.120384	0.584826	-0.003819

18

C	-2.778025	-3.042755	0.628120
C	-1.519007	-2.430798	1.196588
C	-0.771451	-1.563185	0.519965
C	-1.186063	-1.056115	-0.818841
C	-2.534735	-1.472789	-1.295897
C	-3.275561	-2.343458	-0.616071
O	-0.482652	-0.328716	-1.465881
Br	0.858872	-0.884056	1.147968
Br	-1.042396	-3.053930	2.904970
Br	-4.994962	-2.857755	-1.176450
Br	-3.079045	-0.675720	-2.901974
C	-2.589338	-4.534713	0.373541
H	-1.806608	-4.647523	-0.377908
H	-2.293081	-5.046413	1.286401
H	-3.510767	-4.981502	0.007453
N	-3.876479	-2.813272	1.700148
O	-4.098981	-1.659914	1.986622
O	-4.425662	-3.778093	2.164471

18-anion

C	-2.569916	-3.190099	0.460453
C	-1.417629	-2.602540	0.999793
C	-0.833987	-1.462322	0.478560
C	-1.353504	-0.778289	-0.675937
C	-2.509036	-1.426451	-1.237308
C	-3.069645	-2.566591	-0.690934
O	-0.845887	0.260759	-1.149354
Br	0.719335	-0.678645	1.230417
Br	-0.600292	-3.463206	2.498706
Br	-4.569194	-3.372475	-1.562241
Br	-3.203125	-0.594762	-2.793707
C	-3.205229	-4.418897	1.055390
H	-2.784078	-5.337367	0.634286
H	-3.058202	-4.452963	2.132260
H	-4.276258	-4.432424	0.869338

30

C	-2.488429	1.604262	0.350439
C	-0.979370	1.546516	0.049891
C	-1.370064	3.755767	0.152053
C	-2.775659	3.121915	0.292956
H	-3.081991	1.068406	-0.388856
H	-2.728590	1.200725	1.333544
H	-3.421044	3.395055	-0.544003
C	-0.429302	2.737963	0.870100

C	-0.782429	2.017648	-1.409018
H	0.215488	1.780132	-1.775721
H	-1.501479	1.540474	-2.075861
C	-0.997979	3.557569	-1.326601
H	-0.092353	4.110857	-1.573168
H	-1.794358	3.914256	-1.980369
H	-0.524074	0.582025	0.273408
C	-0.613928	2.611970	2.384302
H	-1.637305	2.431988	2.704609
H	-0.000113	1.783774	2.747382
H	-0.263437	3.520009	2.882607
C	1.054910	3.028566	0.635367
H	1.326815	3.989600	1.081571
H	1.654659	2.260137	1.128790
H	1.343603	3.050581	-0.413532
C	-1.395589	5.187063	0.657464
H	-0.496781	5.484210	1.198831
H	-1.621924	5.908922	-0.127568
S	-2.760016	5.332490	1.817330
O	-3.747544	6.261870	1.338176
O	-2.326436	5.408467	3.182386
N	-3.334670	3.705901	1.517288
N	-4.655459	3.485028	1.797660
O	-5.141903	4.138082	2.699335
O	-5.220709	2.627454	1.150684

30-anion

C	-2.589451	1.730120	0.231573
C	-1.078120	1.597332	-0.024870
C	-1.328536	3.830967	0.119712
C	-2.801977	3.260790	0.161253
H	-3.178912	1.197608	-0.517174
H	-2.867242	1.346067	1.214132
H	-3.282708	3.509952	-0.796072
C	-0.499478	2.732401	0.851425
C	-0.794150	2.105289	-1.461536
H	0.186657	1.784181	-1.813886
H	-1.534361	1.718008	-2.164389
C	-0.868018	3.658140	-1.332128
H	0.102904	4.123331	-1.509347
H	-1.580302	4.103901	-2.029071
H	-0.674225	0.603192	0.176118
C	-0.789889	2.586568	2.346204
H	-1.849439	2.620357	2.583845
H	-0.374770	1.641929	2.711332
H	-0.300231	3.390828	2.903843
C	1.015076	2.911751	0.711959
H	1.320911	3.876826	1.128974
H	1.532934	2.132915	1.279060
H	1.373712	2.861993	-0.315135
C	-1.361677	5.213715	0.743188
H	-0.743446	5.300600	1.637359
H	-1.134562	6.026859	0.055824
S	-3.107697	5.379128	1.262857
O	-3.735676	6.190911	0.201447
O	-3.230157	5.992765	2.590395
N	-3.515958	3.860726	1.281739

26b

C	-1.962046	-2.027367	-0.088205
C	-0.578990	-2.027783	-0.040292
C	0.143470	-0.851338	-0.085600
C	-0.570821	0.338824	-0.162156
C	-1.960791	0.339184	-0.209694
C	-2.679331	-0.850693	-0.182411
S	0.200110	-3.597797	0.077011
H	1.226158	-0.857968	-0.066471
H	-0.031378	1.276388	-0.195482
H	-2.495894	1.277272	-0.279847
H	-3.760798	-0.856890	-0.237640
S	-2.747228	-3.597708	-0.025482
O	-3.097603	-3.956049	1.313501
O	-3.673992	-3.760356	-1.097046
O	0.458832	-3.954671	1.437041
N	-1.256456	-4.438718	-0.458607
O	1.197986	-3.760605	-0.928491
N	-1.266591	-5.819079	-0.215868
O	-2.351077	-6.341646	-0.189547
O	-0.188644	-6.343709	-0.104836

26b-anion

C	-1.969787	-2.002118	-0.139498
C	-0.594225	-2.014240	-0.096635
C	0.139430	-0.841213	-0.079321
C	-0.563763	0.357560	-0.113798
C	-1.957710	0.369844	-0.155155
C	-2.682567	-0.816497	-0.164631
S	0.045499	-3.679723	-0.021004
H	1.222109	-0.855716	-0.046835
H	-0.020525	1.294222	-0.110698
H	-2.483726	1.315934	-0.183101
H	-3.765348	-0.812246	-0.197419
S	-2.643338	-3.656202	-0.120062
O	-3.241138	-3.874399	1.186322
O	-3.542975	-3.838078	-1.243863
O	0.535153	-3.897831	1.330079
N	-1.295320	-4.488604	-0.359715
O	1.026004	-3.886725	-1.069829

15c

N	-3.679759	-0.602586	0.611766
C	-2.369108	-1.074636	0.404549
C	-1.528779	-0.076208	-0.279827
C	-2.076887	1.111571	-0.632859
C	-3.432306	1.417136	-0.303668
N	-4.200972	0.596129	0.319082
H	-3.860280	2.376777	-0.565114
O	-1.990682	-2.163044	0.758885
O	-0.279713	-0.375567	-0.610571
Cl	-1.151237	2.295694	-1.474153
N	-4.627988	-1.456812	1.283027
O	-5.332922	-0.916508	2.087780
O	-4.618043	-2.609521	0.958887
C	0.555253	-1.104249	0.316904
H	0.305541	-0.840692	1.343268
H	0.449969	-2.173562	0.159570
H	1.569206	-0.784403	0.089247

15c-anion

N	-3.789367	-0.635496	0.496825

C	-2.543270	-1.087229	0.164232
C	-1.588583	-0.127898	-0.379487
C	-2.000234	1.155093	-0.552678
C	-3.312333	1.516396	-0.195518
N	-4.141805	0.634790	0.309161
H	-3.670392	2.530581	-0.321080
O	-2.229728	-2.287236	0.312621
O	-0.354536	-0.529836	-0.765340
Cl	-0.931880	2.362217	-1.216839
C	0.480344	-0.972028	0.310298
H	0.694246	-0.136338	0.982063
H	0.002830	-1.785470	0.854802
H	1.406916	-1.317956	-0.143774

19-anion

C	-3.894295	0.878397	1.494165
N	-4.157820	0.480164	2.787614
O	-4.636408	-0.654395	2.924278
O	-3.927639	1.226681	3.732368
N	-3.145498	1.966026	1.097994
O	-3.014942	2.123790	-0.123287
O	-2.662068	2.730232	1.925957
N	-4.231284	-0.087069	0.454423
O	-3.320363	-0.683481	-0.079509
O	-5.404302	-0.241480	0.188968

19

C	-3.923777	0.885388	1.289009
N	-3.909126	0.323657	2.709758
O	-4.554632	-0.676085	2.849515
O	-3.282894	0.947325	3.517803
N	-2.768364	1.877334	1.136083
O	-1.738774	1.453320	0.701525
O	-3.034670	2.981111	1.523041
N	-3.818622	-0.306438	0.344682
O	-2.877513	-1.014483	0.565726
O	-4.683884	-0.423983	-0.475096
N	-5.217230	1.650009	1.056968
O	-5.264627	2.225107	0.007902
O	-6.029557	1.598897	1.937791

44

C	-2.385762	2.592982	1.162714
C	-1.798530	1.336140	1.198460
C	-1.275130	0.834955	0.017220
C	-1.307772	1.521493	-1.185968
C	-1.897816	2.777723	-1.195374
C	-2.434189	3.307584	-0.028053
H	-2.805047	3.010192	2.068984
H	-1.754275	0.764366	2.115648
H	-0.888784	1.093258	-2.086525
H	-1.937488	3.339322	-2.119612
H	-2.894553	4.287302	-0.047133
I	-0.421560	-1.053981	0.057339
N	-2.434137	-1.926899	-0.305863
O	-3.388052	-1.219923	-0.486999
O	-2.442307	-3.134962	-0.302759
O	3.578319	0.517261	0.843153
C	2.663610	-0.197617	0.516637
O	1.425371	0.135504	0.435975
C	2.979817	-1.669703	0.142508
F	2.570876	-1.941920	-1.103222
F	4.273183	-1.938208	0.216816
F	2.336537	-2.514896	0.961188

44-anion

C	-3.032209	2.089818	0.746481
C	-2.659070	0.778361	0.477374
C	-1.387868	0.529157	-0.021099
C	-0.486041	1.557894	-0.256847
C	-0.876129	2.863983	0.018984
C	-2.143502	3.132912	0.519366
H	-4.022617	2.290353	1.136445
H	-3.350976	-0.034575	0.654408
H	0.503725	1.344945	-0.646608
H	-0.178662	3.673198	-0.160118
H	-2.438173	4.152761	0.732751
I	-0.813376	-1.446542	-0.426735
O	2.964729	-1.702243	-1.788445
C	2.723052	-0.661406	-1.169961
O	2.403459	0.471130	-1.554134
C	2.927808	-0.778709	0.375532
F	2.657303	-1.997836	0.861462
F	4.218054	-0.524824	0.686126
F	2.192976	0.088957	1.084736

3d

C	-3.766227	1.323290	-0.019186
N	-1.514131	1.215758	0.648288
N	-3.018935	0.159267	-0.557869
C	-1.683599	0.063338	-0.160224
O	-0.863235	-0.761797	-0.383245
N	-3.631416	-0.813867	-1.324652
O	-4.805499	-0.631850	-1.554239
O	-2.961591	-1.740307	-1.702335
C	-2.660033	2.014656	0.774873
O	-2.742543	3.043454	1.365292
N	-0.263553	1.552971	1.209872
O	-0.308285	2.049821	2.301229
O	0.700860	1.320050	0.535739
C	-4.241272	2.271013	-1.114845
H	-3.427024	2.508769	-1.800486
H	-5.067797	1.825334	-1.663624
H	-4.589170	3.189243	-0.641273
C	-4.868454	0.919445	0.956656
H	-5.717091	0.505875	0.417267
H	-4.499085	0.191231	1.680193
H	-5.193208	1.814953	1.487232

3d-anion

C	-3.739650	1.330256	-0.117976
N	-1.353984	1.419068	0.188247
N	-3.038481	0.192929	-0.722868
C	-1.596473	0.291754	-0.514448
O	-0.806321	-0.524227	-0.927175
N	-3.679584	-0.795657	-1.372778
O	-4.897645	-0.692753	-1.465180
O	-3.038373	-1.724037	-1.826480
C	-2.510193	2.063234	0.454338
O	-2.633072	3.111991	1.070491

C	-4.430771	2.223287	-1.141651
H	-3.761796	2.447982	-1.974908
H	-5.336029	1.754441	-1.523516
H	-4.698342	3.159129	-0.649469
C	-4.667294	0.922838	1.020385
H	-5.564091	0.437300	0.638699
H	-4.158481	0.248792	1.712474
H	-4.957095	1.823531	1.562930

35

C	-4.409998	0.142657	0.065328
H	-3.642398	-0.333062	-0.543122
H	-4.971234	0.847620	-0.548089
H	-5.095197	-0.614223	0.445741
C	-3.797246	0.881300	1.245830
O	-2.883822	-0.122730	1.782852
C	-4.875998	1.375275	2.197756
H	-5.702767	1.749885	1.594308
H	-5.240592	0.532915	2.786653
H	-4.536810	2.162854	2.864551
N	-2.376987	-0.012987	3.064649
O	-1.686899	-0.947486	3.352637
O	-2.654990	0.939366	3.731234
C	-2.931217	2.029645	0.722653
F	-2.088763	1.621244	-0.232677
F	-3.704296	2.984241	0.195374
F	-2.185947	2.598836	1.673400

35-anion

C	-4.457583	0.149892	-0.000009
H	-3.694249	-0.232726	-0.680960
H	-5.079956	0.873894	-0.533672
H	-5.088115	-0.687531	0.305790
C	-3.789881	0.721300	1.272424
O	-2.985753	-0.161376	1.891066
C	-4.894435	1.285648	2.196094
H	-5.532559	2.033664	1.716661
H	-5.519826	0.446127	2.506787
H	-4.445080	1.722295	3.090374
C	-2.954042	1.929303	0.811646
F	-1.967864	1.588808	-0.043415
F	-3.666549	2.891153	0.167345
F	-2.345617	2.564654	1.834672

34a

C	-4.390190	0.037889	0.030320
H	-3.589292	-0.391789	-0.569904
H	-4.970834	0.728328	-0.580386
H	-5.044111	-0.751964	0.399285
C	-3.823845	0.797711	1.224155
O	-3.036487	-0.223822	1.900890
C	-4.926099	1.352546	2.111338
H	-5.562363	1.996493	1.503714
H	-5.520801	0.514608	2.476566
H	-4.540572	1.928846	2.948222
C	-2.927637	1.866545	0.724695
N	-2.279229	2.698032	0.278562
N	-2.311450	0.156599	3.011340
O	-1.831610	-0.771819	3.590327
O	-2.220592	1.325465	3.262611

34a-anion

C	-4.308718	0.010989	0.122196
H	-3.411884	-0.385216	-0.357807
H	-4.879251	0.590177	-0.609688
H	-4.919559	-0.829634	0.460358
C	-3.913477	0.833321	1.369525
O	-3.158684	0.157643	2.241795
C	-5.192623	1.437036	1.992018
H	-5.773186	2.035328	1.283747
H	-5.811468	0.607303	2.342647
H	-4.928333	2.058105	2.850266
C	-3.124127	2.036700	0.823422
N	-2.462764	2.910508	0.468482

34b

C	-4.396747	0.002175	0.033306
H	-3.589083	-0.431144	-0.554618
H	-4.966368	0.698881	-0.580447
H	-5.059074	-0.782420	0.397760
C	-3.846931	0.748782	1.244735
O	-3.060285	-0.253795	1.926651
C	-4.951378	1.326496	2.114473
H	-5.564858	1.986974	1.501508
H	-5.564260	0.492873	2.458285
H	-4.567745	1.885050	2.964494
C	-2.947240	1.820495	0.748514
N	-2.309378	2.646310	0.301074
N	-2.341365	0.156068	3.042037
O	-1.916283	-0.756988	3.676529
O	-2.206822	1.334031	3.221008
B	-1.377718	3.849692	-0.334666
F	-0.111703	3.581300	0.098532
F	-1.525572	3.738539	-1.687220
F	-1.895875	5.005111	0.175240

34b-anion

C	-4.489346	-0.038510	0.031229
H	-3.715866	-0.436060	-0.627530
H	-5.152322	0.614571	-0.541897
H	-5.070348	-0.873709	0.429316
C	-3.843124	0.671827	1.235875
O	-2.950640	-0.041733	1.894628
C	-4.935610	1.319583	2.108574
H	-5.596267	1.979803	1.540878
H	-5.527339	0.506123	2.534798
H	-4.476310	1.879195	2.924812
C	-3.074119	1.892386	0.604222
N	-2.351776	2.699379	0.232989
B	-1.352055	3.827500	-0.275126
F	-0.163094	3.643364	0.395522
F	-1.207443	3.653508	-1.633911
F	-1.929374	5.040959	0.028538

45c

S	-1.987328	1.367448	0.028002
C	-1.569414	2.421555	1.405962
H	-1.850081	3.448377	1.181464
H	-2.065105	2.018713	2.287364
H	-0.486071	2.316486	1.510696

C	-1.387786	2.269649	-1.392122
H	-1.722046	1.739599	-2.282102
H	-1.735369	3.299835	-1.344865
H	-0.299088	2.212038	-1.310050
N	-3.811922	1.766145	-0.113244
O	-4.230358	1.789686	-1.227779
O	-4.370271	1.908776	0.928536

45c-anion

S	-2.233018	1.463344	-0.002323
C	-1.537504	2.384866	1.391689
H	-1.790845	3.442127	1.316134
H	-1.970081	1.978037	2.305011
H	-0.454958	2.262983	1.424649
C	-1.350945	2.294060	-1.346614
H	-1.678446	1.844411	-2.283351
H	-1.590439	3.357256	-1.357521
H	-0.275338	2.155235	-1.239522

45e

Se	-1.936322	1.224383	0.022873
C	-1.546103	2.403972	1.490956
H	-1.859083	3.409656	1.221346
H	-2.063747	2.007253	2.360391
H	-0.465269	2.334108	1.615631
C	-1.356039	2.300684	-1.459026
H	-1.698373	1.804125	-2.363039
H	-1.749059	3.306790	-1.338660
H	-0.267651	2.279388	-1.391174
N	-3.860626	1.744789	-0.100938
O	-4.292777	1.888333	-1.207319
O	-4.433642	1.840971	0.945510

45e-anion

Se	-2.249196	1.332195	-0.001621
C	-1.534987	2.384648	1.473816
H	-1.850696	3.420741	1.370874
H	-1.938294	1.967361	2.394323
H	-0.449266	2.315163	1.481384
C	-1.355932	2.314068	-1.426277
H	-1.620418	1.832169	-2.365394
H	-1.698500	3.346588	-1.431524
H	-0.278896	2.269732	-1.278732

45a

P	-1.999285	1.548253	0.010597
C	-1.556981	2.378926	1.508428
H	-1.842208	3.428894	1.439730
H	-2.040918	1.887168	2.351708
H	-0.470853	2.291213	1.600287
C	-1.370990	2.302538	-1.463083
H	-1.726439	1.754575	-2.335045
H	-1.687436	3.345749	-1.492557
H	-0.281076	2.245350	-1.403216
C	-1.785022	-0.211763	0.075136
H	-2.317802	-0.606597	0.941136
H	-2.145813	-0.657176	-0.852208
H	-0.711460	-0.389582	0.182725
N	-3.823574	1.755817	-0.114013
O	-4.299950	1.666360	-1.221654
O	-4.428982	1.939608	0.915369

45a-anion

P	-2.419202	1.586811	-0.016355
C	-1.556676	2.340434	1.425946
H	-1.733890	3.417530	1.432500
H	-1.961422	1.925051	2.350740
H	-0.480627	2.153454	1.389173
C	-1.369034	2.248526	-1.378136
H	-1.655735	1.777778	-2.320368
H	-1.529103	3.323951	-1.474317
H	-0.309785	2.057284	-1.188608
C	-1.745978	-0.125084	0.078758
H	-2.157338	-0.631126	0.954093
H	-2.047718	-0.683952	-0.809194
H	-0.655202	-0.120436	0.145770

33b

C	-0.189154	0.732659	1.155854
H	-0.279093	1.187321	2.139101
H	0.397227	1.364990	0.494490
H	-1.164425	0.514568	0.725033
O	0.599324	-0.475890	1.241202
N	0.076825	-1.435043	2.082142
O	0.727900	-2.439205	2.107638
O	-0.931554	-1.175559	2.673107
B	0.873813	-1.585830	-0.946130
F	2.039091	-2.115089	-0.667234
F	-0.229874	-2.258729	-0.707844
F	0.806667	-0.412718	-1.531823

33b-anion

C	-0.322040	0.521820	0.864786
H	-0.434348	0.962148	1.857359
H	-0.031209	1.317834	0.169614
H	-1.297823	0.132873	0.551570
O	0.648379	-0.494540	0.930035
B	0.876259	-1.208080	-0.280227
F	1.875870	-2.174044	-0.060624
F	-0.290688	-1.871821	-0.742606
F	1.302063	-0.358090	-1.335063

40

O	-2.845431	-0.202515	1.781719
N	-2.205777	0.161879	3.034213
O	-1.297417	-0.512979	3.377465
O	-2.682164	1.147760	3.478566
N	-2.908015	-1.637022	1.559873
O	-3.054024	-2.313418	2.519011
O	-2.862857	-1.885412	0.405593

40-anion

O	0.640967	-0.536147	1.097716
N	0.266627	-1.352275	1.962705
O	-0.394180	-2.355272	1.629266
O	0.552677	-1.165114	3.161285

41

C	-5.278966	-1.475582	-0.190402
C	-3.119708	-0.423986	0.279815

C	-5.143379	0.615176	1.081211
H	-2.078887	-0.632607	0.485103
H	-5.352459	-0.927322	-1.127078
H	-5.794295	-2.423567	-0.258794
H	-5.573656	1.154845	1.914222
H	-5.230272	1.187719	0.156277
H	-3.210701	0.125980	-0.658472
N	-3.748081	0.326123	1.345083
N	-3.850062	-1.675551	0.055646
N	-5.878311	-0.626622	0.837212
C	-3.142429	0.755566	2.502849
O	-3.766148	1.391641	3.332628
C	-1.682816	0.428202	2.674320
H	-1.518222	-0.649688	2.659445
H	-1.092867	0.876724	1.872791
H	-1.358657	0.832021	3.629462
N	-3.512048	-2.733019	0.883569
O	-4.264499	-3.682568	0.912443
O	-2.452005	-2.664567	1.473738
N	-6.414381	-1.264237	1.942464
O	-6.511823	-0.621918	2.967137
O	-6.814298	-2.400044	1.790293

42-anion

C	-5.192959	-1.439907	-0.290837
C	-3.231935	-0.273048	-0.095470
C	-5.057054	0.362392	1.395504
H	-2.145757	-0.299544	-0.110030
H	-5.618817	-0.652348	-0.941795
H	-5.717434	-2.375641	-0.462730
H	-5.343970	0.702092	2.382562
H	-5.464450	1.042070	0.644324
H	-3.585323	0.487838	-0.821454
N	-3.620100	0.289956	1.235699
N	-3.802313	-1.560559	-0.322153
N	-5.683255	-0.926522	1.092873
C	-2.715119	0.562500	2.203663
O	-1.506758	0.421720	2.021571
C	-3.230142	1.086010	3.527192
H	-3.790632	2.012668	3.390325
H	-3.885813	0.358209	4.005988
H	-2.372429	1.279662	4.166207
N	-5.616890	-1.841884	2.088629
O	-5.298133	-1.475919	3.216359
O	-5.923160	-2.995073	1.820427

10b

C	-1.194381	-1.701730	-0.031150
C	0.167798	-1.585623	0.045471
C	-0.463794	0.403942	0.393785
N	-1.579321	-0.387959	0.197512
H	-0.539094	1.463980	0.574571
N	0.603572	-0.314329	0.304195
N	-2.890314	0.124408	0.202444
O	-3.750727	-0.593760	-0.225459
O	-3.003988	1.242063	0.630041
N	1.130111	-2.647867	-0.091687
O	2.288161	-2.396121	0.166713
O	0.726754	-3.736397	-0.463645
C	-2.120321	-2.846476	-0.214766
H	-2.598635	-2.798421	-1.194437
H	-1.560786	-3.771951	-0.136756
H	-2.900226	-2.825394	0.546161

10b-anion

C	-1.149896	-1.668875	-0.079266
C	0.247016	-1.597041	0.008714
C	-0.495441	0.329312	0.317437
N	-1.612329	-0.423056	0.120323
H	-0.550841	1.392877	0.506622
N	0.653293	-0.322126	0.263086
N	1.156258	-2.658854	-0.126863
O	2.361616	-2.445172	-0.019301
O	0.704083	-3.785224	-0.353114
C	-2.045768	-2.832114	-0.334874
H	-1.825782	-3.299389	-1.296675
H	-1.920249	-3.604136	0.426945
H	-3.082898	-2.496198	-0.332060

15b

N	-3.664273	-0.583315	0.585471
C	-2.325016	-1.029554	0.431724
C	-1.545257	-0.067658	-0.314198
C	-2.054313	1.132029	-0.708887
C	-3.421716	1.433580	-0.355020
N	-4.172805	0.605552	0.279054
H	-3.876270	2.381253	-0.608560
O	-1.925946	-2.069659	0.894160
N	-4.600275	-1.416364	1.297717
O	-5.329588	-0.849915	2.061922
O	-4.558482	-2.584806	1.038308
O	-1.284084	1.973235	-1.376647
C	-1.828877	3.232503	-1.803516
H	-2.675241	3.072624	-2.471243
H	-2.119215	3.833752	-0.942007
H	-1.022126	3.723377	-2.339432
Cl	0.086539	-0.489467	-0.640226

15b-anion

N	-3.646838	-0.568164	0.759159
C	-2.375107	-0.984313	0.429390
C	-1.568694	-0.068688	-0.333101
C	-2.050496	1.156548	-0.707504
C	-3.369356	1.466456	-0.306929
N	-4.096131	0.616308	0.390736
H	-3.836051	2.409540	-0.553889
O	-1.966253	-2.106594	0.786889
O	-1.265635	1.983369	-1.419674
C	-1.813472	3.240183	-1.809843
H	-2.687488	3.102284	-2.448993
H	-2.078612	3.839349	-0.936860
H	-1.028347	3.742563	-2.369443
Cl	0.042935	-0.543959	-0.777004

45d

S	-1.975728	1.545800	-0.046867
N	-3.812862	1.458771	-0.691078
O	-4.059937	2.147244	-1.624485
O	-4.501427	0.743609	-0.036283
C	-1.665847	-0.175745	0.089802

C	-1.130800	-0.580914	1.309979
C	-1.979522	-1.071243	-0.933062
C	-0.868686	-1.930891	1.492703
H	-0.923679	0.136142	2.095203
C	-1.722296	-2.412373	-0.721248
H	-2.418504	-0.733220	-1.864697
C	-1.163367	-2.837129	0.483395
H	-0.446134	-2.271040	2.429002
H	-1.960682	-3.129568	-1.495737
H	-0.965867	-3.890994	0.636368
C	-1.175766	2.205249	-1.456952
C	-0.470783	1.431340	-2.376561
C	-1.287601	3.593105	-1.571088
C	0.112275	2.075467	-3.453516
H	-0.351142	0.364329	-2.249312
C	-0.708685	4.211436	-2.665737
H	-1.817349	4.174422	-0.824608
C	-0.015050	3.453768	-3.602555
H	0.672148	1.498277	-4.177705
H	-0.791507	5.284377	-2.779854
H	0.444755	3.943178	-4.452237

45d-anion

S	-1.784638	1.667956	0.244761
C	-1.577936	-0.100909	0.161981
C	-1.002576	-0.754313	1.246521
C	-2.039298	-0.831893	-0.931592
C	-0.893747	-2.141355	1.240024
H	-0.637631	-0.182272	2.091288
C	-1.907157	-2.212546	-0.940633
H	-2.496045	-0.321156	-1.771506
C	-1.339096	-2.871252	0.146760
H	-0.446512	-2.645567	2.088110
H	-2.258746	-2.777754	-1.795508
H	-1.244615	-3.950220	0.138310
C	-1.097575	2.225069	-1.303542
C	0.019962	1.627194	-1.882043
C	-1.675034	3.344009	-1.897683
C	0.546201	2.145822	-3.057323
H	0.477241	0.761051	-1.418354
C	-1.129581	3.868218	-3.063672
H	-2.550958	3.802173	-1.453263
C	-0.022079	3.269146	-3.649770
H	1.411857	1.673788	-3.506540
H	-1.583193	4.740172	-3.519331
H	0.395668	3.672333	-4.564092

45f

Se	-1.955361	1.675199	0.154874
N	-3.872779	1.525270	-0.567255
O	-4.136503	2.172793	-1.531718
O	-4.573196	0.804727	0.079814
C	-1.645467	-0.201547	0.187441
C	-1.105186	-0.693940	1.369615
C	-1.974370	-1.024246	-0.886541
C	-0.865909	-2.058541	1.465036
H	-0.876735	-0.030237	2.194283
C	-1.738053	-2.382799	-0.765764
H	-2.404196	-0.619818	-1.795918
C	-1.181588	-2.895759	0.403345
H	-0.441185	-2.463503	2.374400
H	-1.990362	-3.041164	-1.587043
H	-0.999068	-3.960155	0.485998
C	-1.155880	2.303692	-1.450250
C	-0.382308	1.492535	-2.272826
C	-1.342326	3.660970	-1.701872
C	0.196263	2.063909	-3.396039
H	-0.208784	0.450275	-2.043116
C	-0.765572	4.208418	-2.836660
H	-1.924916	4.278055	-1.028226
C	0.000032	3.411208	-3.679929
H	0.808321	1.452034	-4.046232
H	-0.906729	5.259543	-3.052881
H	0.457814	3.846479	-4.559669

45f-anion

Se	-1.675114	1.774347	0.436703
C	-1.523920	-0.134829	0.263200
C	-1.055362	-0.862332	1.351787
C	-1.926679	-0.790729	-0.896998
C	-0.994793	-2.250407	1.280246
H	-0.734745	-0.348734	2.250536
C	-1.842735	-2.174697	-0.968658
H	-2.302000	-0.224909	-1.741794
C	-1.381186	-2.908323	0.119938
H	-0.632842	-2.813726	2.132091
H	-2.149276	-2.682185	-1.875592
H	-1.323969	-3.988469	0.062052
C	-1.030716	2.320710	-1.290602
C	0.089971	1.730008	-1.868080
C	-1.676015	3.372598	-1.932207
C	0.552046	2.186013	-3.095580
H	0.599463	0.916911	-1.363982
C	-1.194220	3.838097	-3.151531
H	-2.554043	3.824627	-1.486255
C	-0.084620	3.243775	-3.737929
H	1.420111	1.719504	-3.545968
H	-1.698359	4.659595	-3.646451
H	0.283515	3.600848	-4.692025

45b

P	-1.861011	1.518466	0.004604
N	-3.703147	1.698880	-0.298100
O	-4.082738	1.319127	-1.381123
O	-4.389505	2.142252	0.587669
C	-1.633535	-0.238550	0.108955
C	-1.076403	-0.798603	1.259672
C	-1.978032	-1.033893	-0.990472
C	-0.876212	-2.169916	1.311234
H	-0.791174	-0.179648	2.100480
C	-1.777646	-2.400879	-0.918091
H	-2.388344	-0.591536	-1.890243
C	-1.230089	-2.966480	0.230073
H	-0.441478	-2.612427	2.198121
H	-2.043844	-3.025033	-1.761387
H	-1.072362	-4.037087	0.277376
C	-1.552101	2.409733	1.500031
C	-2.198705	2.031720	2.682723
C	-0.651354	3.476399	1.476830
C	-1.939869	2.737769	3.843345

H	-2.894673	1.201501	2.693970
C	-0.400154	4.169862	2.651241
H	-0.149203	3.759714	0.560017
C	-1.043469	3.803254	3.826052
H	-2.438352	2.458384	4.762548
H	0.297195	4.997581	2.644711
H	-0.844910	4.350509	4.739660
C	-1.150601	2.233540	-1.452217
C	-0.083561	1.582188	-2.071107
C	-1.630337	3.458420	-1.924396
C	0.506757	2.169943	-3.180494
H	0.282552	0.634858	-1.694102
C	-1.036633	4.025409	-3.038014
H	-2.453889	3.962856	-1.430524
C	0.029277	3.382208	-3.661917
H	1.336447	1.676046	-3.669562
H	-1.404289	4.969197	-3.419615
H	0.489879	3.832315	-4.532861

45b-anion

P	-2.440449	1.515565	-0.156017
C	-1.760898	-0.179783	0.045219
C	-0.784542	-0.531307	0.975875
C	-2.271484	-1.163911	-0.804646
C	-0.322686	-1.841344	1.048906
H	-0.378113	0.213793	1.650057
C	-1.801197	-2.469011	-0.740650
H	-3.042926	-0.905902	-1.523448
C	-0.825671	-2.810742	0.189443
H	0.435064	-2.102652	1.778200
H	-2.202503	-3.219776	-1.410931
H	-0.463049	-3.829964	0.247879
C	-1.807437	2.366648	1.347224
C	-2.617410	2.325845	2.484083
C	-0.583316	3.031917	1.413004
C	-2.205577	2.923269	3.668714
H	-3.578409	1.823161	2.441163
C	-0.175498	3.638765	2.595477
H	0.060340	3.077007	0.541689
C	-0.983497	3.583613	3.725233
H	-2.843012	2.882482	4.543951
H	0.776980	4.154038	2.633918
H	-0.663800	4.057898	4.645424
C	-1.347278	2.214704	-1.459751
C	-0.240596	1.562263	-1.999876
C	-1.676645	3.489293	-1.928921
C	0.526743	2.178086	-2.984458
H	0.033807	0.571468	-1.656222
C	-0.903081	4.108915	-2.900025
H	-2.544498	4.002668	-1.526160
C	0.201506	3.451689	-3.432701
H	1.384204	1.660156	-3.397767
H	-1.167203	5.100689	-3.247229
H	0.802160	3.929531	-4.197338

31a

S	-0.787271	-0.648065	-1.329416
O	-0.788158	-2.069675	-1.509534
O	-0.296497	0.196051	-2.384218
N	0.263517	-0.261624	-0.007667
C	1.425721	0.652595	-0.146715
H	1.307814	1.029040	-1.161871
C	1.307974	1.842280	0.795333
H	1.492872	1.572770	1.833593
H	2.030677	2.602362	0.497397
C	2.735451	-0.118948	-0.102533
C	3.841523	0.368115	0.585328
C	2.851448	-1.315769	-0.808032
C	5.044811	-0.330891	0.567569
H	3.776337	1.292946	1.144278
C	4.049841	-2.015347	-0.823167
H	1.998500	-1.713351	-1.348977
C	5.153226	-1.523493	-0.134116
H	5.897323	0.060204	1.110059
H	4.121284	-2.945688	-1.373686
H	6.089231	-2.068597	-0.143694
H	0.310775	2.274729	0.706298
C	-2.363346	-0.068076	-0.805584
H	-2.690044	-0.647286	0.052777
H	-2.268102	0.992822	-0.581709
H	-3.023221	-0.229380	-1.659587
N	0.107825	-0.967186	1.156714
O	-0.882619	-1.665413	1.262162
O	0.963473	-0.830116	2.003046

31a-anion

S	-0.798552	-0.084139	-1.289713
O	-0.304438	-0.879794	-2.435560
O	-1.386501	1.190221	-1.754179
N	0.199748	0.039509	-0.092822
C	1.417615	0.805060	-0.396665
H	1.427467	1.133371	-1.446272
C	1.471636	2.067169	0.458935
H	1.488660	1.812608	1.522293
H	2.346862	2.681671	0.233882
C	2.645480	-0.087848	-0.238895
C	3.743817	0.242349	0.551310
C	2.672731	-1.299974	-0.936186
C	4.840510	-0.615213	0.644849
H	3.758730	1.170731	1.108880
C	3.764990	-2.149749	-0.853863
H	1.811427	-1.561292	-1.543073
C	4.857682	-1.810639	-0.057467
H	5.682152	-0.341461	1.270840
H	3.767476	-3.081284	-1.408386
H	5.710545	-2.475338	0.012567
H	0.577544	2.661703	0.266723
C	-2.121834	-1.020243	-0.560791
H	-1.734198	-1.981325	-0.229798
H	-2.532312	-0.459628	0.276112
H	-2.876965	-1.159818	-1.333141

31e

S	-0.893402	-0.297413	-1.054278
O	-0.256889	-0.945524	-2.162074
O	-1.583211	0.931195	-1.290338
N	0.385256	0.038366	0.049439
N	0.344935	-0.289911	1.377426
O	1.351988	-0.080752	2.009463
O	-0.680388	-0.771767	1.813314

C	1.590889	0.701855	-0.525817
H	1.323250	0.833794	-1.576286
C	1.757044	2.105033	0.047422
H	1.919518	2.105346	1.121509
H	2.601533	2.574808	-0.454760
C	2.836519	-0.163089	-0.483807
C	3.843845	0.037718	0.456728
C	2.986433	-1.165027	-1.440290
C	4.980777	-0.760511	0.445023
H	3.745398	0.808802	1.210756
C	4.118833	-1.968962	-1.446452
H	2.210220	-1.316950	-2.181695
C	5.118240	-1.768524	-0.501715
H	5.757221	-0.597494	1.182558
H	4.222867	-2.746384	-2.193650
H	6.003761	-2.392755	-0.505546
O	0.608319	2.887113	-0.279670
C	-0.355463	3.000074	0.656161
C	-1.471896	3.873444	0.181790
H	-1.091119	4.887408	0.043564
H	-1.836256	3.515292	-0.780661
H	-2.272552	3.876714	0.915958
O	-0.284446	2.473094	1.736615
C	-2.028787	-1.491091	-0.326658
C	-3.281969	-1.073392	0.140340
C	-1.749647	-2.854684	-0.450559
C	-4.266829	-2.021866	0.368698
C	-2.749491	-3.787311	-0.211741
C	-4.006184	-3.364231	0.164524
H	-5.241906	-1.708735	0.718255
H	-2.534349	-4.842457	-0.314657
C	-0.379774	-3.436502	-0.757242
F	0.607453	-2.679786	-0.266651
F	-0.161279	-3.619459	-2.057378
F	-0.254861	-4.635950	-0.177928
C	-3.663713	0.357570	0.498944
F	-4.657725	0.346142	1.398933
F	-4.117511	1.053289	-0.545213
F	-2.665547	1.033153	1.061346
C	-5.114099	-4.358615	0.381520
F	-6.012196	-4.311171	-0.611134
F	-5.778579	-4.115747	1.516251
F	-4.659139	-5.610784	0.444247

31e-anion

S	-0.983844	-0.096909	-1.363598
O	-0.571030	-0.713764	-2.626223
O	-1.966659	0.962730	-1.591589
N	0.142871	0.174956	-0.346007
C	1.410293	0.651862	-0.881574
H	1.384122	0.765016	-1.970486
C	1.717610	2.029237	-0.287631
H	1.823667	1.961895	0.794419
H	2.629267	2.444085	-0.720639
C	2.569870	-0.270364	-0.550421
C	2.653249	-0.900455	0.690275
C	3.594003	-0.465411	-1.473054
C	3.737461	-1.711990	0.999009
H	1.853777	-0.762895	1.409616
C	4.681155	-1.279159	-1.168728
H	3.534942	0.018083	-2.443140
C	4.755434	-1.904869	0.069175
H	3.789015	-2.198774	1.966076
H	5.465391	-1.427558	-1.901885
H	5.598718	-2.541560	0.309052
O	0.651440	2.920011	-0.619614
C	-0.203526	3.277446	0.350884
C	-1.411578	3.952187	-0.220707
H	-1.108924	4.752148	-0.897519
H	-1.965744	3.206983	-0.794765
H	-2.028341	4.350196	0.580992
O	-0.003672	3.097420	1.526372
C	-1.963356	-1.364277	-0.480767
C	-3.042882	-1.003022	0.341987
C	-1.718365	-2.729248	-0.683402
C	-3.896431	-1.971663	0.850746
C	-2.584629	-3.691169	-0.173649
C	-3.680182	-3.306566	0.568370
H	-4.736420	-1.685236	1.470030
H	-2.399602	-4.740177	-0.356922
C	-0.521343	-3.295168	-1.431447
F	0.610946	-2.633017	-1.217479
F	-0.720268	-3.388684	-2.750397
F	-0.280896	-4.557960	-1.016801
C	-3.376112	0.417630	0.773184
F	-4.072641	0.387501	1.931537
F	-4.166403	1.060586	-0.093334
F	-2.312947	1.172470	1.022410
C	-4.651708	-4.318022	1.098086
F	-5.851788	-4.210988	0.505096
F	-4.865717	-4.163733	2.412769
F	-4.235888	-5.573360	0.911016

31d

S	-0.537039	-1.515629	-0.906544
O	-0.256375	-2.899710	-0.683815
O	-0.295946	-0.916596	-2.187663
N	0.442721	-0.572235	0.146553
N	0.541184	-0.999999	1.456649
O	1.462213	-0.567004	2.106004
O	-0.314270	-1.762954	1.849587
C	1.402130	0.443653	-0.351300
H	1.086486	0.596308	-1.382879
C	1.211971	1.777749	0.358373
H	1.373210	1.715299	1.431306
H	1.913462	2.487571	-0.077536
C	2.829781	-0.073092	-0.377617
C	3.841885	0.497782	0.386599
C	3.129904	-1.142533	-1.218615
C	5.139988	0.006227	0.306120
H	3.629809	1.321339	1.057560
C	4.423642	-1.639490	-1.292610
H	2.348453	-1.587668	-1.826638
C	5.433500	-1.062783	-0.530451
H	5.921197	0.458870	0.904723
H	4.644243	-2.471750	-1.949944
H	6.445084	-1.445716	-0.589581
O	-0.089203	2.309144	0.090142
C	-1.048532	2.113124	1.015467
C	-2.254989	2.952897	0.737167

H	-2.037992	3.967987	1.079832
H	-2.462344	2.996475	-0.331159
H	-3.110298	2.564213	1.284079
O	-0.912245	1.376787	1.958743
C	-2.190701	-1.142595	-0.407829
C	-2.756472	0.033131	-0.880939
C	-2.879894	-2.041098	0.391965
C	-4.059105	0.334872	-0.519602
H	-2.200327	0.701335	-1.526933
C	-4.185873	-1.745216	0.746353
H	-2.407227	-2.951387	0.733958
C	-4.738028	-0.561594	0.286949
H	-4.534273	1.243282	-0.861544
H	-4.758905	-2.418285	1.367973
N	-6.124643	-0.237573	0.676835
O	-6.584246	0.820629	0.301118
O	-6.725809	-1.045779	1.352792

31d-anion

S	-0.795017	-0.085467	-1.455026
O	-0.383972	-1.160492	-2.374906
O	-1.262270	1.100991	-2.189851
N	0.153583	0.186616	-0.255007
C	1.443412	0.801847	-0.565065
H	1.541384	1.029286	-1.636197
C	1.551441	2.139221	0.160272
H	1.435459	2.013698	1.236666
H	2.497037	2.636114	-0.056019
C	2.589567	-0.139980	-0.211459
C	3.679594	0.229045	0.574344
C	2.542572	-1.443437	-0.712944
C	4.699866	-0.681155	0.847920
H	3.751765	1.226259	0.990730
C	3.560600	-2.347264	-0.449825
H	1.685084	-1.735669	-1.310186
C	4.647356	-1.968668	0.335541
H	5.536251	-0.376464	1.466294
H	3.506880	-3.352113	-0.852805
H	5.441668	-2.674641	0.546787
O	0.535725	3.008354	-0.345508
C	-0.576743	3.187286	0.374590
C	-1.616061	3.913304	-0.424447
H	-1.196139	4.835475	-0.828855
H	-1.902102	3.275268	-1.262605
H	-2.477734	4.136803	0.199354
O	-0.709709	2.832833	1.520254
C	-2.225519	-0.703922	-0.571457
C	-2.938016	0.170540	0.241885
C	-2.616613	-2.024112	-0.724476
C	-4.055400	-0.281440	0.920350
H	-2.613292	1.198243	0.349905
C	-3.735208	-2.491573	-0.048229
H	-2.046662	-2.681434	-1.367797
C	-4.428830	-1.608571	0.759957
H	-4.627166	0.375266	1.560785
H	-4.060524	-3.517666	-0.145459
N	-5.612623	-2.097128	1.479997
O	-6.213720	-1.313720	2.189040
O	-5.932554	-3.260475	1.331850

31b

S	-0.788847	-1.055149	-1.235382
O	-0.588712	-2.384236	-1.727356
O	-0.591651	0.071412	-2.104784
N	0.358536	-0.755121	0.021387
N	0.381931	-1.627222	1.076601
O	1.216218	-1.433304	1.934183
O	-0.438600	-2.524709	1.078731
C	1.310723	0.384514	-0.001139
H	1.089839	0.856948	-0.957379
C	1.000179	1.395803	1.092910
H	1.277299	1.037004	2.082467
H	1.544385	2.318391	0.889286
C	2.744035	-0.118269	-0.073504
C	3.760028	0.486941	0.657998
C	3.061342	-1.172053	-0.929535
C	5.073610	0.043909	0.536077
H	3.538368	1.305441	1.330780
C	4.370050	-1.617895	-1.047275
H	2.279950	-1.655152	-1.507453
C	5.382840	-1.009325	-0.313221
H	5.854997	0.524597	1.112538
H	4.599382	-2.441186	-1.713129
H	6.404968	-1.356481	-0.403133
C	-2.350556	-0.914048	-0.438446
C	-2.762477	0.348104	-0.029076
C	-3.132494	-2.046611	-0.274559
C	-4.005249	0.470313	0.573122
H	-2.127588	1.213477	-0.178813
C	-4.379113	-1.904449	0.318700
H	-2.770249	-3.012851	-0.599807
C	-4.809317	-0.652385	0.742828
H	-4.347610	1.442258	0.904311
H	-5.010080	-2.773431	0.454625
H	-5.781088	-0.548597	1.210146
H	-0.066244	1.624688	1.078610

31b-anion

S	-0.815827	0.024341	-1.238042
O	-0.436920	-0.940675	-2.287521
O	-1.245504	1.307776	-1.823545
N	0.178646	0.147293	-0.043319
C	1.438050	0.840196	-0.362675
H	1.470538	1.135992	-1.420984
C	1.542430	2.122236	0.456503
H	1.539817	1.897460	1.526537
H	2.445875	2.690188	0.221529
C	2.615496	-0.109043	-0.162583
C	3.730034	0.202144	0.612447
C	2.579384	-1.352889	-0.800766
C	4.780440	-0.705313	0.750379
H	3.793460	1.154654	1.123950
C	3.625767	-2.253561	-0.673425
H	1.705363	-1.602201	-1.394241
C	4.734634	-1.933323	0.108030
H	5.635246	-0.444946	1.364097
H	3.578373	-3.210837	-1.180103
H	5.550313	-2.638517	0.214540
C	-2.242556	-0.670628	-0.416400
C	-3.068587	0.160595	0.329895

C	-2.516769	-2.024365	-0.535991
C	-4.178847	-0.374593	0.968800
H	-2.843147	1.217795	0.403744
C	-3.629637	-2.556768	0.107031
H	-1.865391	-2.649753	-1.133685
C	-4.459242	-1.733658	0.858297
H	-4.827138	0.268046	1.552355
H	-3.848616	-3.614007	0.017408
H	-5.327395	-2.149549	1.355490
H	0.677758	2.750307	0.238299

31c

S	-0.608864	-1.374529	-0.957106
O	-0.398791	-2.771977	-0.729144
O	-0.285263	-0.792526	-2.230065
N	0.413364	-0.489586	0.121675
N	0.454350	-0.910110	1.432359
O	1.375135	-0.513277	2.108583
O	-0.442348	-1.635009	1.807053
C	1.435099	0.471385	-0.355399
H	1.156276	0.629760	-1.396524
C	1.293485	1.820863	0.336136
H	1.421252	1.759862	1.413584
H	2.039998	2.492186	-0.085998
C	2.834401	-0.117917	-0.336277
C	3.857255	0.414210	0.441478
C	3.095919	-1.221554	-1.145441
C	5.127411	-0.150289	0.407255
H	3.672730	1.263185	1.088605
C	4.361410	-1.790425	-1.174203
H	2.305491	-1.636969	-1.762748
C	5.381922	-1.253405	-0.397266
H	5.916673	0.271388	1.017810
H	4.552657	-2.648541	-1.807162
H	6.371591	-1.693377	-0.420276
O	0.027946	2.411352	0.024955
C	-0.963370	2.282529	0.927599
C	-2.118705	3.174849	0.599500
H	-1.859856	4.184786	0.928358
H	-2.295752	3.206093	-0.474424
H	-3.006169	2.839805	1.129896
O	-0.887609	1.565073	1.892030
C	-2.244168	-0.913389	-0.507746
C	-2.744531	0.280295	-1.012314
C	-2.997732	-1.758800	0.294792
C	-4.042280	0.642953	-0.683479
H	-2.138014	0.906456	-1.655641
C	-4.295106	-1.382894	0.609271
H	-2.575268	-2.683359	0.664321
C	-4.811851	-0.185388	0.125923
H	-4.450851	1.571006	-1.062908
H	-4.902296	-2.025799	1.233709
H	-5.825092	0.102063	0.379166

31c-anion

S	-0.792915	-0.080371	-1.434038
O	-0.371271	-1.137608	-2.372896
O	-1.257553	1.118957	-2.152169
N	0.176728	0.181174	-0.241177
C	1.457520	0.800960	-0.574381
H	1.536826	1.027409	-1.647438
C	1.576217	2.139143	0.148417
H	1.481794	2.012763	1.226742
H	2.515985	2.639790	-0.085519
C	2.613002	-0.136390	-0.238951
C	3.709566	0.232460	0.537926
C	2.564822	-1.438440	-0.744428
C	4.734638	-0.676218	0.799147
H	3.782944	1.228420	0.957272
C	3.587626	-2.340513	-0.494201
H	1.701358	-1.729584	-1.333791
C	4.680630	-1.962064	0.282824
H	5.575710	-0.371793	1.411343
H	3.532752	-3.344190	-0.900026
H	5.478286	-2.667006	0.484748
O	0.545372	3.001277	-0.337059
C	-0.532593	3.214819	0.424447
C	-1.601179	3.917315	-0.357149
H	-1.203703	4.846811	-0.768418
H	-1.889973	3.274402	-1.190335
H	-2.453966	4.129128	0.282693
O	-0.614288	2.911152	1.589638
C	-2.210037	-0.718777	-0.553104
C	-3.026069	0.167024	0.139579
C	-2.484165	-2.077152	-0.567214
C	-4.126062	-0.317720	0.833048
H	-2.797074	1.226694	0.131967
C	-3.588197	-2.559065	0.129411
H	-1.838020	-2.746360	-1.121520
C	-4.406862	-1.681628	0.828875
H	-4.765838	0.366967	1.376767
H	-3.807529	-3.619992	0.123434
H	-5.266786	-2.057746	1.370221

38a

P	-1.184885	-0.420271	-0.728424
O	-1.770610	-1.346988	-1.676363
O	-2.256534	0.591024	0.015022
O	-0.394814	-0.974622	0.517697
C	0.918750	-1.431463	0.307319
C	1.930712	-0.492110	0.131888
C	1.151302	-2.792124	0.352831
C	3.233743	-0.978301	0.006090
C	2.455417	-3.247586	0.221853
H	0.320292	-3.470978	0.497294
C	3.494459	-2.339444	0.050596
H	4.043344	-0.273266	-0.139810
H	2.657060	-4.310751	0.254349
H	4.511993	-2.694023	-0.054202
N	-3.325096	0.022985	0.722606
O	-3.273871	-1.152515	0.925328
O	-4.143516	0.822206	1.038339
O	-0.245707	0.659663	-1.376924
C	0.569120	1.495837	-0.595522
C	1.646445	0.960100	0.106275
C	0.296284	2.849769	-0.634095
C	2.468684	1.861182	0.787238
C	1.128903	3.720907	0.052614
H	-0.552506	3.202589	-1.206709
C	2.216306	3.224014	0.761877

H	3.308654	1.476464	1.353464
H	0.926497	4.784179	0.031673
H	2.866930	3.899300	1.303054

38a-anion

P	-1.310329	-0.301098	-0.615089
O	-1.634487	-1.358895	-1.598319
O	-2.338560	0.528796	0.051918
O	-0.379490	-0.904347	0.602384
C	0.879254	-1.378522	0.338273
C	1.929944	-0.473442	0.149458
C	1.106830	-2.747705	0.358547
C	3.220247	-0.984650	-0.002597
C	2.396871	-3.234505	0.198852
H	0.267495	-3.414579	0.513452
C	3.456943	-2.351760	0.021629
H	4.042153	-0.294281	-0.154952
H	2.573682	-4.303074	0.215431
H	4.464652	-2.727956	-0.103535
O	-0.183918	0.650157	-1.349787
C	0.587775	1.497248	-0.597614
C	1.671325	0.983123	0.123224
C	0.334915	2.861325	-0.639239
C	2.502113	1.883383	0.792802
C	1.171332	3.738326	0.036934
H	-0.511461	3.216580	-1.214495
C	2.258679	3.249001	0.753052
H	3.342291	1.500224	1.361145
H	0.973522	4.802833	0.003787
H	2.912336	3.929231	1.284843

38e

P	0.087346	-0.664471	-0.778279
O	-0.540112	-1.644689	-1.642469
N	-1.095769	0.155338	0.207244
O	1.037426	-1.141997	0.374726
C	2.428682	-1.260656	0.179062
C	3.203018	-0.119928	-0.010028
C	2.962968	-2.531675	0.259582
C	4.583735	-0.305693	-0.115186
C	4.336966	-2.685168	0.150155
H	2.303378	-3.375712	0.416148
C	5.145895	-1.570173	-0.036940
H	5.215147	0.560081	-0.275663
H	4.771346	-3.674819	0.211814
H	6.218544	-1.686635	-0.127161
N	-0.921939	0.343067	1.621203
O	0.094205	0.867810	1.964731
O	-1.833961	-0.041706	2.305887
O	0.857988	0.469470	-1.546085
C	1.461714	1.502709	-0.807605
C	2.613605	1.235437	-0.075971
C	0.884581	2.755272	-0.878053
C	3.198942	2.309004	0.598052
C	1.483170	3.802967	-0.192999
H	-0.014454	2.896961	-1.465790
C	2.641288	3.577261	0.542592
H	4.092536	2.133430	1.185016
H	1.044103	4.791608	-0.235380
H	3.109907	4.391752	1.080485

C	-3.555532	-1.198456	0.079157
F	-4.375021	-0.968542	1.078934
S	-2.671275	0.403684	-0.362919
O	-2.545413	0.453388	-1.779604
F	-2.679405	-2.121059	0.412994
F	-4.232169	-1.585011	-0.980905
O	-3.253927	1.446227	0.409592

38e-anion

P	0.006416	-0.691134	-0.623878
O	-0.225110	-1.795268	-1.560423
N	-1.186064	-0.057638	0.265330
O	1.048041	-1.023832	0.562349
C	2.386582	-1.195060	0.249297
C	3.174707	-0.071227	-0.006090
C	2.920850	-2.472166	0.283179
C	4.540234	-0.270001	-0.216034
C	4.280737	-2.645445	0.065952
H	2.267404	-3.310352	0.491920
C	5.091060	-1.542809	-0.181273
H	5.168942	0.587930	-0.425171
H	4.705000	-3.641465	0.091929
H	6.151819	-1.675414	-0.353776
O	0.777650	0.491936	-1.409374
C	1.374259	1.519663	-0.701754
C	2.579393	1.282663	-0.037450
C	0.798397	2.778348	-0.745477
C	3.206933	2.367538	0.577548
C	1.437400	3.840574	-0.120814
H	-0.134824	2.912758	-1.278724
C	2.644296	3.634846	0.538782
H	4.141241	2.203881	1.102113
H	0.992600	4.827499	-0.152298
H	3.144740	4.461063	1.028253
C	-3.628436	-1.079808	-0.129536
F	-4.860340	-0.808124	-0.560071
S	-2.543607	0.396375	-0.340009
O	-2.558822	0.627199	-1.769552
F	-3.706076	-1.428775	1.152990
F	-3.166071	-2.116770	-0.817123
O	-3.184322	1.376531	0.506541

38d

P	-1.156088	-0.379593	-0.814133
S	-1.844758	-1.625417	-2.082024
S	-2.535516	1.008157	0.085691
O	-0.383767	-0.907704	0.479286
C	0.919709	-1.395821	0.291400
C	1.953443	-0.477216	0.132714
C	1.125663	-2.761072	0.341955
C	3.249672	-0.986513	0.035611
C	2.423724	-3.240850	0.237696
H	0.279398	-3.423974	0.472023
C	3.483074	-2.352682	0.086654
H	4.074970	-0.296511	-0.093879
H	2.605326	-4.307443	0.275445
H	4.495544	-2.726829	0.002922
N	-3.616904	-0.138152	0.932279
O	-3.318284	-1.301106	0.989397
O	-4.591779	0.385138	1.411058

O	-0.158652	0.697475	-1.444889
C	0.640701	1.524549	-0.638876
C	1.690537	0.978369	0.097566
C	0.389883	2.883329	-0.691550
C	2.503891	1.871088	0.798855
C	1.211221	3.746220	0.019396
H	-0.428456	3.250251	-1.300066
C	2.269090	3.237315	0.763322
H	3.321779	1.477453	1.390759
H	1.024078	4.811961	-0.012096
H	2.911231	3.905583	1.323007

38d-anion

P	-1.296746	-0.314787	-0.630993
S	-1.719183	-1.709909	-1.958275
S	-2.716443	0.742535	0.237148
O	-0.369901	-0.904767	0.596424
C	0.898264	-1.380063	0.336400
C	1.943904	-0.472699	0.148365
C	1.121172	-2.748685	0.363743
C	3.234634	-0.983689	-0.002739
C	2.411477	-3.234420	0.205338
H	0.281683	-3.414040	0.521939
C	3.470053	-2.350596	0.023636
H	4.056462	-0.293552	-0.155264
H	2.589462	-4.302571	0.225550
H	4.477646	-2.726838	-0.102033
O	-0.174685	0.653585	-1.349553
C	0.601185	1.501820	-0.588605
C	1.684188	0.983236	0.125760
C	0.346161	2.864365	-0.633166
C	2.518253	1.882608	0.792617
C	1.186198	3.739602	0.040606
H	-0.500086	3.219376	-1.208249
C	2.274639	3.247870	0.753425
H	3.361141	1.498832	1.356201
H	0.990728	4.804381	0.007562
H	2.931293	3.927399	1.282370

38b

P	-1.188015	-0.414640	-0.734446
O	-1.762568	-1.333051	-1.695028
O	-2.253300	0.588653	0.016174
O	-0.394977	-0.982472	0.507728
C	0.910973	-1.440826	0.302085
C	1.927619	-0.501039	0.137930
C	1.139061	-2.800931	0.337341
C	3.226376	-0.984055	0.016979
C	2.440135	-3.263212	0.209906
H	0.307841	-3.480828	0.471439
C	3.471313	-2.349080	0.053373
H	4.045299	-0.286049	-0.117529
H	2.641631	-4.325714	0.234174
N	-3.328533	0.012244	0.718502
O	-3.275059	-1.164532	0.907717
O	-4.144454	0.809804	1.038555
O	-0.237315	0.670068	-1.365340
C	0.574143	1.494268	-0.580405
C	1.647276	0.951367	0.120462
C	0.305454	2.850950	-0.612330
C	2.476755	1.839589	0.806267
C	1.138558	3.714008	0.074256
H	-0.541046	3.212473	-1.181989
C	2.222021	3.198407	0.777568
H	3.315605	1.451640	1.370666
H	0.947123	4.780188	0.060725
C	4.890978	-2.808823	-0.085201
F	5.674242	-2.309026	0.885078
F	5.435769	-2.412342	-1.246960
F	5.008693	-4.138859	-0.033488
C	3.126740	4.157156	1.490061
F	2.446152	4.986968	2.295628
F	3.797836	4.940044	0.627584
F	4.040669	3.540896	2.245824

38b-anion

P	-1.292645	-0.342075	-0.663451
O	-1.583398	-1.393056	-1.660728
O	-2.329919	0.477370	-0.002828
O	-0.369285	-0.949492	0.563465
C	0.889583	-1.412256	0.315804
C	1.937960	-0.494872	0.154216
C	1.125797	-2.779101	0.322618
C	3.230502	-0.988724	0.022994
C	2.419238	-3.257941	0.182649
H	0.290994	-3.455746	0.452587
C	3.464958	-2.357275	0.038274
H	4.056324	-0.296867	-0.104478
H	2.607724	-4.323479	0.189350
O	-0.154165	0.626896	-1.365961
C	0.592795	1.469717	-0.596645
C	1.667807	0.958636	0.137619
C	0.326439	2.833802	-0.630295
C	2.485584	1.853384	0.825613
C	1.142811	3.709711	0.060137
H	-0.514015	3.188644	-1.213137
C	2.222871	3.212123	0.783810
H	3.321489	1.476187	1.402226
H	0.943851	4.774770	0.036282
C	4.875292	-2.830470	-0.106995
F	5.668945	-2.366727	0.876357
F	5.436345	-2.415207	-1.257154
F	4.979566	-4.164448	-0.087444
C	3.108283	4.183263	1.495880
F	2.416627	5.013739	2.295411
F	3.779924	4.975504	0.638115
F	4.027190	3.584561	2.262742

38f

P	0.087107	-0.665025	-0.790417
O	-0.536297	-1.639673	-1.661233
N	-1.085720	0.157256	0.195531
O	1.040973	-1.150018	0.360484
C	2.425541	-1.273384	0.174629
C	3.205817	-0.133767	-0.011800
C	2.955007	-2.544644	0.259867
C	4.581845	-0.319163	-0.109675
C	4.327146	-2.707396	0.156807
H	2.294675	-3.387988	0.413608
C	5.128712	-1.590756	-0.028725

H	5.222854	0.541197	-0.266673
H	4.759608	-3.696955	0.219942
N	-0.901781	0.355549	1.608275
O	0.121804	0.873590	1.940791
O	-1.813474	-0.014396	2.299531
O	0.873223	0.466728	-1.552878
C	1.475480	1.494143	-0.818170
C	2.621693	1.223056	-0.081231
C	0.901947	2.750145	-0.892051
C	3.209969	2.287978	0.600857
C	1.495009	3.792023	-0.203073
H	0.007020	2.897779	-1.483760
C	2.647161	3.549622	0.538150
H	4.098249	2.111346	1.194050
H	1.063478	4.785153	-0.240519
C	-3.558573	-1.176428	0.092577
F	-4.358578	-0.935132	1.104654
S	-2.665800	0.415559	-0.369378
O	-2.543899	0.451636	-1.786537
F	-2.684185	-2.104750	0.414271
F	-4.255429	-1.558249	-0.955339
O	-3.231755	1.469670	0.398921
C	6.618156	-1.725927	-0.129183
F	7.240180	-1.231487	0.954977
F	7.112912	-1.057440	-1.182027
F	7.009426	-2.997895	-0.253895
C	3.268430	4.697382	1.275140
F	2.398173	5.282827	2.112892
F	3.685838	5.661289	0.437203
F	4.326398	4.330438	2.004111

38f-anion

P	0.048151	-0.752596	-0.608054
O	-0.162010	-1.868003	-1.533801
N	-1.147538	-0.100498	0.255129
O	1.077787	-1.067994	0.598071
C	2.416839	-1.237746	0.320456
C	3.206771	-0.112445	0.064373
C	2.955698	-2.510473	0.390787
C	4.573840	-0.302566	-0.103895
C	4.319714	-2.684279	0.215852
H	2.303648	-3.349672	0.596517
C	5.119064	-1.576753	-0.029105
H	5.210418	0.551421	-0.308486
H	4.750656	-3.675154	0.272540
O	0.842005	0.419288	-1.396894
C	1.417261	1.457324	-0.699831
C	2.606845	1.237021	-0.005171
C	0.836956	2.714774	-0.780685
C	3.221882	2.329918	0.603963
C	1.455325	3.787816	-0.165995
H	-0.084326	2.837845	-1.335785
C	2.648617	3.586481	0.521645
H	4.144530	2.182814	1.151428
H	1.014629	4.776297	-0.222912
C	-3.582055	-1.133265	-0.160586
F	-3.666174	-1.475873	1.122664
S	-2.501112	0.345495	-0.370745
O	-2.494292	0.564334	-1.801621
F	-3.110488	-2.170573	-0.840868
F	-4.810782	-0.866318	-0.601002
O	-3.150247	1.331375	0.461165
C	6.600050	-1.723310	-0.184628
F	7.267813	-1.238596	0.878975
F	7.067710	-1.054740	-1.251134
F	6.980327	-2.998137	-0.326674
C	3.301788	4.766456	1.169503
F	2.489654	5.372510	2.052373
F	3.641414	5.709413	0.272276
F	4.419665	4.443134	1.829041

38c

P	-1.170602	-0.413462	-0.736564
O	-1.733801	-1.340093	-1.694675
O	-2.236699	0.587789	0.008513
O	-0.373347	-0.972962	0.509583
C	0.932096	-1.423311	0.314113
C	1.943459	-0.478553	0.147726
C	1.166496	-2.784897	0.358720
C	3.247008	-0.948566	0.029111
C	2.466034	-3.242718	0.238248
H	0.338274	-3.467970	0.493509
C	3.477550	-2.310013	0.077014
H	4.068073	-0.258106	-0.108771
H	2.688169	-4.299845	0.266426
N	-3.313478	0.009298	0.713807
O	-3.254732	-1.166222	0.905773
O	-4.130023	0.806353	1.029972
O	-0.218931	0.672592	-1.368609
C	0.579707	1.506129	-0.587911
C	1.653816	0.972222	0.124635
C	0.300215	2.859920	-0.629376
C	2.469294	1.864165	0.814215
C	1.116816	3.737563	0.059209
H	-0.543948	3.212300	-1.207733
C	2.187456	3.215668	0.765638
H	3.309658	1.503601	1.392368
H	0.925661	4.800960	0.047613
N	4.861638	-2.787700	-0.055732
O	5.057789	-3.983454	0.021362
O	5.733907	-1.961982	-0.234740
N	3.063391	4.137730	1.502624
O	3.990580	3.664366	2.127866
O	2.813168	5.324468	1.444688

38c-anion

P	-1.271829	-0.346401	-0.669625
O	-1.538580	-1.406782	-1.661106
O	-2.316042	0.469279	-0.019109
O	-0.345257	-0.937373	0.567827
C	0.912222	-1.393558	0.329602
C	1.955978	-0.470997	0.165387
C	1.153655	-2.763034	0.343633
C	3.252373	-0.951919	0.036569
C	2.444262	-3.237412	0.207290
H	0.321249	-3.441971	0.474509
C	3.472483	-2.317985	0.060391
H	4.080527	-0.268140	-0.093215
H	2.652720	-4.297853	0.215756
O	-0.133159	0.631184	-1.367983

C	0.599296	1.482231	-0.602686
C	1.675846	0.980598	0.143267
C	0.320820	2.843892	-0.645520
C	2.479070	1.878848	0.833846
C	1.120433	3.733684	0.045523
H	-0.518150	3.188482	-1.235975
C	2.189812	3.230865	0.771831
H	3.316591	1.528877	1.422803
H	0.921586	4.795745	0.024495
N	4.843947	-2.808454	-0.082587
O	5.026738	-4.010952	-0.059025
O	5.735209	-1.991303	-0.216988
N	3.044982	4.163767	1.506560
O	3.967015	3.707742	2.156179
O	2.793378	5.351535	1.430700

38g

P	0.101368	-0.656652	-0.789738
O	-0.509257	-1.632224	-1.667055
N	-1.074511	0.152292	0.197713
O	1.062523	-1.139086	0.358709
C	2.443482	-1.255705	0.177415
C	3.219756	-0.112996	-0.008493
C	2.976726	-2.527931	0.267543
C	4.597012	-0.287652	-0.104019
C	4.346009	-2.689689	0.169227
H	2.318316	-3.372379	0.422031
C	5.126085	-1.560282	-0.014726
H	5.245737	0.563914	-0.259995
H	4.795552	-3.670244	0.234775
N	-0.889625	0.353844	1.610277
O	0.135353	0.870993	1.940746
O	-1.801670	-0.012648	2.301815
O	0.885348	0.483392	-1.546227
C	1.480830	1.510759	-0.814218
C	2.630926	1.242248	-0.075976
C	0.903118	2.764083	-0.890257
C	3.215925	2.304404	0.604940
C	1.488048	3.813079	-0.203963
H	0.008504	2.908309	-1.482898
C	2.635575	3.555809	0.527543
H	4.103881	2.149384	1.202864
H	1.062863	4.805972	-0.236493
C	-3.544128	-1.186949	0.098654
F	-4.338917	-0.947737	1.114826
S	-2.657615	0.407205	-0.368138
O	-2.533962	0.440022	-1.785051
F	-2.664333	-2.111828	0.415135
F	-4.244904	-1.569785	-0.945865
O	-3.223541	1.462045	0.398524
N	6.583750	-1.719586	-0.124854
O	7.035876	-2.844355	-0.059337
O	7.255719	-0.719396	-0.274512
N	3.261988	4.660843	1.269314
O	2.728855	5.750235	1.218440
O	4.277354	4.423746	1.891475

38g-anion

P	0.067586	-0.746184	-0.593128
O	-0.131141	-1.870620	-1.508809
N	-1.126538	-0.087422	0.261927
O	1.101673	-1.045127	0.617244
C	2.436532	-1.216053	0.343386
C	3.225041	-0.092008	0.075164
C	2.975259	-2.491325	0.424831
C	4.591093	-0.276430	-0.099446
C	4.334166	-2.669622	0.243620
H	2.323753	-3.327895	0.641280
C	5.114925	-1.553662	-0.013845
H	5.235467	0.565799	-0.314152
H	4.779923	-3.652463	0.302044
O	0.864386	0.421955	-1.391193
C	1.430783	1.466708	-0.706198
C	2.621777	1.255604	-0.003783
C	0.844406	2.720135	-0.800522
C	3.229956	2.349949	0.598862
C	1.449658	3.804081	-0.192281
H	-0.073986	2.834614	-1.361600
C	2.635226	3.594196	0.494764
H	4.150749	2.228654	1.153548
H	1.014060	4.791320	-0.252286
C	-3.559764	-1.121570	-0.158583
F	-3.646607	-1.459068	1.125399
S	-2.479288	0.356796	-0.371313
O	-2.463383	0.568623	-1.802810
F	-3.085088	-2.160149	-0.834367
F	-4.786598	-0.855705	-0.603560
O	-3.128652	1.346949	0.454276
N	6.557194	-1.733507	-0.211136
O	7.004260	-2.861694	-0.143346
O	7.232466	-0.747127	-0.431642
N	3.286539	4.736233	1.143895
O	2.741573	5.820120	1.070209
O	4.338310	4.542179	1.721875

39b

P	0.337261	0.359285	0.437486
O	-0.271316	-0.640157	1.478435
N	0.794557	1.584855	1.548751
O	-0.868270	0.908409	-0.391272
C	-2.045163	1.311335	0.285028
C	-2.871557	0.352201	0.863178
C	-2.339091	2.661075	0.274017
C	-4.045679	0.814822	1.462622
C	-3.510406	3.090259	0.881227
H	-1.672887	3.355278	-0.224108
C	-4.360244	2.165534	1.476339
H	-4.708713	0.098398	1.932699
H	-3.755196	4.144880	0.884681
H	-5.272922	2.497538	1.955123
N	0.856745	2.907158	1.015311
O	0.986160	2.962587	-0.180254
O	0.737129	3.812636	1.786845
C	-1.268118	-1.563027	1.094272
C	-2.548304	-1.091292	0.828989
C	-0.927184	-2.899371	1.081426
C	-3.524234	-2.048187	0.543300
C	-1.916783	-3.827255	0.789942
H	0.089714	-3.194536	1.307610
C	-3.212830	-3.399416	0.524297

H	-4.532444	-1.718706	0.321917
H	-1.673449	-4.881989	0.774169
H	-3.985944	-4.122520	0.297328
P	1.414489	1.190165	3.090595
O	2.377507	2.388866	3.339415
C	2.722234	2.698386	4.678668
C	1.762547	3.257014	5.517365
C	4.026846	2.463431	5.058682
C	2.178690	3.594951	6.806673
C	4.409076	2.805065	6.348342
H	4.720255	2.028163	4.349820
C	3.484186	3.370985	7.218179
H	1.457268	4.023408	7.492331
H	5.427493	2.627074	6.669379
H	3.778975	3.635181	8.225879
O	0.257962	1.269857	4.135432
C	-0.348121	2.522159	4.380239
C	0.368299	3.490286	5.074350
C	-1.640894	2.695788	3.934000
C	-0.291397	4.692325	5.333430
C	-2.269667	3.904692	4.200347
H	-2.136342	1.894238	3.396445
C	-1.594080	4.897622	4.900990
H	0.236444	5.475953	5.863788
H	-3.284162	4.067053	3.857816
H	-2.082233	5.842114	5.105499
N	2.098247	-0.200324	2.997530
C	2.689850	-1.278937	5.350379
F	2.245312	-0.186821	5.961669
S	1.752118	-1.540566	3.782991
O	2.400300	-2.642353	3.132929
F	3.982074	-1.139358	5.103407
F	2.507574	-2.323695	6.143355
O	0.380456	-1.660933	4.191872
N	1.427536	-0.205620	-0.503566
C	3.153904	0.785850	-2.225678
F	2.391399	1.853199	-2.389803
S	2.981576	0.112820	-0.518732
O	3.749413	-1.097575	-0.531868
F	2.810636	-0.133355	-3.114853
F	4.421281	1.124165	-2.419191
O	3.366113	1.188888	0.359099

39b-anion

P	0.399686	0.154592	0.657005
O	-0.382191	-0.944062	1.510677
N	0.911169	1.268397	1.634480
O	-0.733355	0.757498	-0.290855
C	-1.916481	1.193665	0.304957
C	-2.850303	0.248199	0.727585
C	-2.144172	2.555220	0.389405
C	-4.052033	0.726147	1.255349
C	-3.348191	3.004046	0.912925
H	-1.381405	3.240846	0.040865
C	-4.299940	2.088214	1.348299
H	-4.791374	0.014476	1.604096
H	-3.538600	4.067663	0.985692
H	-5.237495	2.436378	1.763939
C	-1.353656	-1.755316	0.933668
C	-2.587988	-1.203053	0.591385
C	-1.083809	-3.106036	0.809658
C	-3.571386	-2.074350	0.118759
C	-2.076747	-3.949674	0.331881
H	-0.109322	-3.477439	1.101778
C	-3.321216	-3.432791	-0.010209
H	-4.537305	-1.670964	-0.162571
H	-1.877374	-5.009147	0.230503
H	-4.097453	-4.088588	-0.384015
P	1.474009	1.076538	3.088475
O	2.402076	2.354960	3.299123
C	2.784160	2.689194	4.596717
C	1.858680	3.298495	5.444202
C	4.093278	2.454166	4.972470
C	2.303884	3.686096	6.708656
C	4.509587	2.840768	6.238998
H	4.766637	1.976160	4.271461
C	3.614758	3.459564	7.104426
H	1.603094	4.152883	7.391196
H	5.531463	2.656947	6.546962
H	3.935921	3.760748	8.093645
O	0.346367	1.258054	4.205498
C	-0.251436	2.504150	4.365343
C	0.457864	3.514220	5.015925
C	-1.556645	2.667171	3.939046
C	-0.208448	4.716896	5.254042
C	-2.195045	3.876061	4.181551
H	-2.055858	1.848721	3.431319
C	-1.521791	4.897339	4.843313
H	0.320252	5.519999	5.754293
H	-3.217166	4.017329	3.851838
H	-2.018415	5.840785	5.032530
N	2.292878	-0.246262	3.413100
C	2.697523	-0.889800	5.978018
F	2.119549	0.225159	6.412532
S	1.958016	-1.426682	4.375178
O	2.741156	-2.585211	4.023558
F	4.001905	-0.669039	5.853323
F	2.520677	-1.838180	6.895779
O	0.564662	-1.615902	4.695065
N	1.390213	-0.597045	-0.336252
C	2.547956	1.188547	-1.936687
F	1.847569	2.172411	-1.375635
S	2.842298	-0.154205	-0.707626
O	3.491257	-1.191664	-1.468149
F	1.866183	0.733792	-2.982121
F	3.702533	1.684242	-2.368186
O	3.592075	0.502434	0.334488

15a

N	-3.581180	-0.605728	0.581500
C	-2.185208	-0.861684	0.591207
C	-1.456095	0.170144	-0.128823
C	-2.084905	1.252446	-0.614378
C	-3.502779	1.386933	-0.431969
N	-4.218293	0.494843	0.158202
H	-4.038249	2.256882	-0.789130
O	-1.699057	-1.810608	1.157252
N	-4.465280	-1.530207	1.235250
O	-5.347167	-1.047298	1.889857
O	-4.235342	-2.692233	1.048199

H	-0.387336	0.021288	-0.212921
H	-1.545063	2.034753	-1.132095

15a-anion

N	-3.590435	-0.609677	0.590260
C	-2.241274	-0.832390	0.454055
C	-1.447575	0.195331	-0.174501
C	-2.046366	1.335192	-0.609801
C	-3.435764	1.462950	-0.422498
N	-4.147975	0.517321	0.154845
H	-3.978446	2.344096	-0.743075
O	-1.736846	-1.902599	0.870565
H	-0.382360	0.025546	-0.282598
H	-1.484298	2.129782	-1.088184

14

C	-8.758832	-0.850055	0.100193
C	-7.387026	-0.884369	0.085674
C	-6.692056	0.330922	-0.001996
C	-7.388635	1.564407	-0.039055
C	-8.802616	1.548284	-0.032762
C	-9.474884	0.361278	0.027459
H	-9.300313	-1.784748	0.174435
H	-6.873621	-1.831284	0.156724
C	-6.667544	2.772718	-0.051599
H	-9.324938	2.496457	-0.066396
H	-10.556762	0.343515	0.034476
C	-5.293042	2.780051	-0.009505
C	-4.622285	1.571637	0.013175
H	-7.221079	3.704101	-0.085557
H	-4.717841	3.693907	-0.010538
H	-3.542811	1.489617	0.020036
N	-5.311645	0.429712	-0.000889
N	-4.496913	-0.795401	-0.080413
O	-3.592926	-0.847203	0.694975
O	-4.829286	-1.570382	-0.922722

14-anion

C	-8.724010	-0.855501	0.000312
C	-7.355817	-0.834704	0.000127
C	-6.661066	0.399340	0.000212
C	-7.401230	1.608253	0.000507
C	-8.816836	1.556331	0.000710
C	-9.463387	0.350945	0.000605
H	-9.251243	-1.801896	0.000231
H	-6.776026	-1.750293	-0.000096
C	-6.678989	2.825274	0.000611
H	-9.372506	2.487549	0.000945
H	-10.545853	0.313365	0.000753
C	-5.314674	2.789250	0.000417
C	-4.667478	1.531617	0.000110
H	-7.220642	3.764890	0.000845
H	-4.722260	3.694849	0.000488
H	-3.581890	1.492194	-0.000036
N	-5.295957	0.382049	0.000028

32e

Si	-1.898826	1.581662	-0.032283
C	-1.638798	-0.251374	0.094733
C	-0.611073	-0.769385	0.883719
C	-2.450102	-1.132033	-0.628431
C	-0.394459	-2.139911	0.957245
H	0.028160	-0.107364	1.458292
C	-2.246361	-2.499940	-0.555714
H	-3.253867	-0.755662	-1.252934
C	-1.219126	-2.993531	0.241166
H	0.402779	-2.533786	1.574174
H	-2.881795	-3.178976	-1.112066
C	-1.548991	2.485738	1.557690
C	-1.802630	1.887056	2.798412
C	-1.001284	3.769657	1.530386
C	-1.527859	2.557958	3.977455
H	-2.216945	0.886917	2.847432
C	-0.726450	4.453488	2.709016
H	-0.777488	4.253021	0.586040
C	-0.992472	3.840680	3.922681
H	-1.725343	2.087293	4.933485
H	-0.304889	5.449614	2.674783
C	-1.025372	2.347271	-1.486114
C	0.121474	1.756978	-2.021074
C	-1.497517	3.539273	-2.046458
C	0.786508	2.340950	-3.091252
H	0.503583	0.827922	-1.611443
C	-0.843049	4.127279	-3.117046
H	-2.387844	4.017014	-1.652271
C	0.295108	3.520058	-3.633014
H	1.673385	1.875785	-3.503022
H	-1.217716	5.046979	-3.549174
O	-3.532565	1.859241	-0.562429
N	-4.560610	1.491393	0.235622
O	-5.650261	1.751384	-0.185045
O	-4.282903	0.941244	1.275172
C	-0.998462	-4.475602	0.282623
F	-0.193689	-4.850457	1.283262
F	-0.437129	-4.928936	-0.852855
F	-2.147133	-5.153644	0.428741
C	-0.734212	4.559595	5.212855
F	-1.877850	4.945972	5.805751
F	0.007607	5.660618	5.052427
F	-0.095864	3.783507	6.102666
C	1.025529	4.176276	-4.765569
F	1.922145	5.079890	-4.328540
F	0.199001	4.833349	-5.590393
F	1.706698	3.297466	-5.511927

32e-anion

Si	-2.334921	1.456455	-0.209038
C	-1.653016	-0.313218	-0.005228
C	-0.375552	-0.602644	0.482247
C	-2.473204	-1.388099	-0.362575
C	0.075426	-1.910650	0.611059
H	0.289969	0.204370	0.773673
C	-2.041043	-2.701866	-0.242954
H	-3.471647	-1.185434	-0.735348
C	-0.765225	-2.954683	0.246921
H	1.067981	-2.115094	0.993156
H	-2.689138	-3.524793	-0.523223
C	-1.831168	2.369230	1.389802
C	-2.789019	2.552239	2.392927
C	-0.542672	2.859323	1.625406

C	-2.480921	3.193104	3.585436
H	-3.799519	2.191892	2.231111
C	-0.213605	3.501372	2.812510
H	0.226080	2.750029	0.866407
C	-1.189945	3.666334	3.786877
H	-3.237063	3.329166	4.350056
H	0.789372	3.877289	2.974822
C	-1.232991	2.262749	-1.545179
C	-0.354771	1.550885	-2.363665
C	-1.335420	3.646104	-1.754179
C	0.397451	2.180570	-3.351852
H	-0.244613	0.478738	-2.237596
C	-0.596144	4.291337	-2.731475
H	-2.006431	4.236921	-1.137281
C	0.271122	3.548868	-3.529216
H	1.072400	1.607111	-3.975035
H	-0.688743	5.361944	-2.878025
O	-3.872892	1.492677	-0.501922
C	-0.304445	-4.373855	0.344098
F	0.795728	-4.514993	1.094476
F	-0.010706	-4.898519	-0.862418
F	-1.241870	-5.178131	0.874785
C	-0.843827	4.316542	5.087959
F	-1.859810	5.036171	5.589442
F	0.206623	5.144030	4.988885
F	-0.522971	3.415673	6.038387
C	1.078831	4.267368	-4.561989
F	2.011833	5.068458	-4.010556
F	0.322229	5.066238	-5.335097
F	1.730170	3.436837	-5.385566

32c

O	0.448959	-0.376233	0.900846
N	0.327631	-1.412698	1.740170
O	1.207465	-2.229242	1.686590
O	-0.640415	-1.437703	2.467411
Si	-0.788130	0.884443	0.892105
C	-2.405924	0.122304	0.415603
H	-3.081780	0.910376	0.071043
H	-2.275117	-0.586008	-0.406183
H	-2.880266	-0.392657	1.251329
C	-0.779756	1.719582	2.543500
H	0.240365	1.972363	2.842813
H	-1.346915	2.652305	2.474832
H	-1.232280	1.101421	3.319315
C	-0.070452	1.938016	-0.449677
H	-0.712654	2.806227	-0.618999
H	0.923913	2.298794	-0.177979
H	0.003391	1.383262	-1.387625

32c-anion

O	0.581718	-0.443787	1.001598
Si	-0.502230	0.696046	0.843618
C	-2.217026	0.074215	0.300247
H	-2.938424	0.893058	0.209726
H	-2.160844	-0.427326	-0.671647
H	-2.623121	-0.647368	1.016810
C	-0.840130	1.673663	2.440973
H	0.070914	2.161675	2.802739
H	-1.597681	2.451208	2.297579
H	-1.193874	1.010533	3.237331
C	-0.063653	2.027659	-0.442989
H	-0.843342	2.792653	-0.520246
H	0.871609	2.534208	-0.183085
H	0.068581	1.587061	-1.436803

32f

Si	-1.930868	1.591270	-0.035047
C	-1.692506	-0.246972	0.085912
C	-0.657712	-0.779666	0.858821
C	-2.526654	-1.110553	-0.629974
C	-0.456176	-2.150372	0.925453
H	-0.000385	-0.128502	1.424831
C	-2.342067	-2.482597	-0.574282
H	-3.334713	-0.721046	-1.240100
C	-1.308032	-2.970118	0.206782
H	0.338565	-2.573284	1.523658
H	-2.985318	-3.158001	-1.120565
C	-1.566510	2.490523	1.557022
C	-1.797937	1.882923	2.796395
C	-1.041568	3.785531	1.523307
C	-1.527323	2.552267	3.978558
H	-2.194920	0.876160	2.847844
C	-0.766928	4.473509	2.696436
H	-0.838586	4.275764	0.578064
C	-1.020560	3.838392	3.898272
H	-1.703826	2.088444	4.938882
H	-0.367025	5.477402	2.677406
C	-1.036746	2.344081	-1.485482
C	0.135102	1.762198	-1.976219
C	-1.518284	3.514956	-2.080777
C	0.820504	2.333912	-3.038295
H	0.524380	0.849718	-1.537628
C	-0.849786	4.098309	-3.144847
H	-2.426612	3.983721	-1.719698
C	0.308225	3.490619	-3.598827
H	1.726874	1.890069	-3.425784
H	-1.217637	5.001101	-3.611485
O	-3.555218	1.898303	-0.560756
N	-4.585790	1.552284	0.248282
O	-5.671088	1.846348	-0.156899
O	-4.307743	0.985277	1.278454
N	1.025986	4.100746	-4.731391
O	0.556368	5.105279	-5.226993
O	2.049157	3.567743	-5.110838
N	-1.106665	-4.427502	0.276509
O	-0.234406	-4.846374	1.010479
O	-1.823401	-5.134073	-0.403417
N	-0.738517	4.563289	5.149713
O	-1.029815	4.023051	6.197566
O	-0.232097	5.663933	5.067163

32f-anion

Si	-2.358626	1.457941	-0.232494
C	-1.672648	-0.309927	-0.021592
C	-0.384993	-0.586746	0.451938
C	-2.498568	-1.388559	-0.350982
C	0.073307	-1.887453	0.591784
H	0.282376	0.226083	0.720399
C	-2.067326	-2.701776	-0.221960

H	-3.502033	-1.192556	-0.712482
C	-0.783331	-2.922587	0.247708
H	1.068319	-2.099581	0.957811
H	-2.706610	-3.535632	-0.477571
C	-1.853740	2.361514	1.372817
C	-2.810912	2.526552	2.380065
C	-0.565019	2.855937	1.605163
C	-2.505209	3.150073	3.581079
H	-3.819710	2.162788	2.218035
C	-0.230363	3.485269	2.794842
H	0.200938	2.756993	0.842529
C	-1.213375	3.617138	3.763097
H	-3.247097	3.276631	4.357657
H	0.765643	3.867662	2.970910
C	-1.245579	2.270985	-1.557060
C	-0.325740	1.571258	-2.343413
C	-1.380783	3.648521	-1.780067
C	0.437984	2.207810	-3.313275
H	-0.191314	0.504048	-2.204343
C	-0.633925	4.310473	-2.740427
H	-2.084435	4.225703	-1.187982
C	0.267356	3.569759	-3.490950
H	1.149724	1.662089	-3.917269
H	-0.741442	5.373412	-2.907051
O	-3.892836	1.497236	-0.530938
N	1.068931	4.255577	-4.510662
O	0.902516	5.451042	-4.662470
O	1.861559	3.596783	-5.156943
N	-0.311510	-4.304848	0.391765
O	0.821485	-4.484534	0.796792
O	-1.075023	-5.205875	0.100187
N	-0.871488	4.276921	5.028371
O	-1.726508	4.343833	5.891084
O	0.252627	4.724221	5.156652

32d

Si	-2.008051	1.536959	-0.048345
C	-1.571022	-0.263136	0.100461
C	-0.557222	-0.659022	0.978057
C	-2.202457	-1.239797	-0.678197
C	-0.180318	-1.993485	1.073888
H	-0.056329	0.077649	1.598644
C	-1.830938	-2.574280	-0.579959
H	-2.994696	-0.960843	-1.365055
C	-0.818168	-2.951379	0.295316
H	0.607369	-2.285429	1.758007
H	-2.331491	-3.320747	-1.184979
H	-0.528357	-3.992746	0.371580
C	-1.744914	2.469491	1.532520
C	-2.721325	2.455313	2.534155
C	-0.545152	3.145179	1.774447
C	-2.507841	3.106362	3.741971
H	-3.661649	1.935649	2.375443
C	-0.327061	3.791940	2.985770
H	0.228865	3.173656	1.013681
C	-1.309127	3.774275	3.968410
H	-3.275318	3.091908	4.506731
H	0.607643	4.311251	3.160205
H	-1.141735	4.281250	4.911361
C	-1.219455	2.336492	-1.529168
C	-0.730930	1.546704	-2.575312
C	-1.073321	3.726506	-1.617024
C	-0.126444	2.127648	-3.684091
H	-0.816761	0.466159	-2.528149
C	-0.463036	4.308093	-2.719556
H	-1.439069	4.361211	-0.817152
C	0.008528	3.508248	-3.755526
H	0.243024	1.501625	-4.487353
H	-0.354847	5.384866	-2.771994
H	0.483693	3.962901	-4.616807
O	-3.729585	1.352807	-0.288737
N	-4.493353	2.445816	-0.489465
O	-5.656500	2.228281	-0.681833
O	-3.945174	3.522333	-0.454037

32d-anion

Si	-2.357376	1.448819	-0.209938
C	-1.665454	-0.315411	-0.001022
C	-0.384699	-0.593224	0.490824
C	-2.465776	-1.403383	-0.362208
C	0.080470	-1.898018	0.614289
H	0.267971	0.224429	0.785138
C	-2.011558	-2.714795	-0.243918
H	-3.465639	-1.212605	-0.739834
C	-0.734820	-2.964258	0.244626
H	1.076716	-2.085873	0.998979
H	-2.652247	-3.541257	-0.530947
H	-0.375811	-3.982495	0.339919
C	-1.889761	2.369939	1.394699
C	-2.858594	2.544836	2.389439
C	-0.609420	2.874881	1.650750
C	-2.567309	3.190550	3.587572
H	-3.863380	2.171799	2.215291
C	-0.305054	3.520399	2.845250
H	0.169219	2.771578	0.899751
C	-1.286392	3.679708	3.818412
H	-3.338208	3.315541	4.340010
H	0.694530	3.904521	3.015747
H	-1.054951	4.186503	4.748153
C	-1.252140	2.268321	-1.534963
C	-0.335365	1.575723	-2.331384
C	-1.382203	3.645910	-1.761254
C	0.421186	2.222726	-3.306727
H	-0.201003	0.507256	-2.191310
C	-0.634629	4.302629	-2.731058
H	-2.081828	4.223397	-1.162107
C	0.273746	3.588990	-3.508112
H	1.125786	1.658982	-3.907972
H	-0.756228	5.369485	-2.882373
H	0.860807	4.096544	-4.264743
O	-3.895957	1.470568	-0.522921

22g

C	-3.417940	0.640529	-0.114001
C	-2.482835	1.312497	0.661094
C	-1.193239	0.814884	0.757373
C	-0.894534	-0.351684	0.070932
C	-1.821484	-1.046126	-0.695387
C	-3.103824	-0.532959	-0.787717
H	-2.749496	2.220436	1.186345

H	-0.456219	1.324255	1.365189
H	-1.564591	-1.963541	-1.212522
H	-3.848386	-1.046944	-1.381453
S	0.707638	-1.076587	0.246689
O	0.640214	-2.376419	0.862073
C	1.327149	-1.362123	-1.543250
N	1.745369	-0.149391	1.028761
N	2.014830	1.032595	0.399403
O	2.781502	1.773177	0.968317
O	1.482438	1.273598	-0.684785
F	2.605465	-1.058702	-1.582750
F	0.652099	-0.656948	-2.417614
F	1.167985	-2.646535	-1.784600
C	-4.824082	1.168967	-0.184717
F	-5.425236	0.848329	-1.334968
F	-4.869434	2.500157	-0.070730
F	-5.583210	0.669333	0.803028

22g-anion

C	-3.334973	0.583320	-0.101733
C	-2.341682	1.230359	0.623846
C	-1.115643	0.605936	0.798882
C	-0.904275	-0.651183	0.251274
C	-1.899680	-1.300804	-0.465749
C	-3.127637	-0.678204	-0.644091
H	-2.521587	2.211933	1.045284
H	-0.322550	1.084700	1.360282
H	-1.715205	-2.284505	-0.877564
H	-3.914250	-1.171688	-1.200855
S	0.712736	-1.432138	0.502581
O	0.465660	-2.860638	0.235868
C	1.415676	-0.938121	-1.182186
N	1.506110	-0.856212	1.581663
F	2.643642	-1.451363	-1.314870
F	1.523884	0.391939	-1.293703
F	0.687932	-1.363896	-2.222133
C	-4.669363	1.246973	-0.257473
F	-5.341094	0.801993	-1.326815
F	-4.562847	2.577000	-0.387273
F	-5.463772	1.034288	0.807669

22h

C	-2.726850	0.146919	0.015088
C	-1.918223	1.237459	-0.240637
C	-0.540647	1.090791	-0.250185
C	0.018186	-0.162698	0.027514
C	-0.809315	-1.283874	0.154219
C	-2.186439	-1.113596	0.164082
H	-2.358653	2.206578	-0.438199
H	-2.832798	-1.973761	0.278775
S	1.786251	-0.344972	0.400800
O	1.974331	-1.514362	1.212976
C	2.623438	-0.612385	-1.294300
N	2.499718	1.000379	0.808025
N	2.080588	1.475774	2.038960
O	2.731085	2.388328	2.481301
O	1.098277	0.980813	2.575330
F	3.248119	-1.760256	-1.193817
F	3.465322	0.347394	-1.565820
F	1.686087	-0.678118	-2.212731
C	-4.221534	0.329318	0.033029
F	-4.827250	-0.629585	0.734368
F	-4.732718	0.298689	-1.204439
F	-4.563811	1.502563	0.569917
C	0.252388	2.320139	-0.658520
F	-0.508675	3.151281	-1.368075
F	1.291144	1.992051	-1.436126
F	0.711901	3.020048	0.380920
C	-0.328178	-2.727445	0.159764
F	-0.043377	-3.197068	1.369632
F	0.743286	-2.887106	-0.627825
F	-1.279516	-3.520865	-0.342019

22h-anion

C	-2.708890	0.167024	0.066484
C	-1.910134	1.251506	-0.244942
C	-0.533025	1.095794	-0.306968
C	0.063889	-0.140232	-0.030349
C	-0.775064	-1.248956	0.127554
C	-2.154604	-1.089751	0.200898
H	-2.359253	2.217905	-0.439783
H	-2.793233	-1.949024	0.358536
S	1.868806	-0.175044	0.504152
O	1.953872	-1.464609	1.192035
C	2.661729	-0.613699	-1.184713
N	2.315811	1.107673	1.009667
F	3.317506	-1.773268	-1.140865
F	3.543656	0.326803	-1.522249
F	1.758620	-0.692916	-2.167373
C	-4.195720	0.348620	0.148938
F	-4.769513	0.339568	-1.065432
F	-4.524006	1.515283	0.716925
C	0.227985	2.320164	-0.779593
F	-0.544309	3.018868	-1.637980
F	1.331745	2.015303	-1.463906
F	0.548060	3.178075	0.190145
C	-0.297271	-2.690327	0.118100
F	-0.032849	-3.198268	1.322283
F	0.763221	-2.874528	-0.669896
F	-1.266216	-3.477718	-0.395861
F	-4.781288	-0.619548	0.860133

22l

C	-3.407065	0.625243	-0.084226
C	-2.486265	1.316262	0.682368
C	-1.195235	0.820667	0.764640
C	-0.899858	-0.347079	0.078974
C	-1.829522	-1.049397	-0.676946
C	-3.115275	-0.544514	-0.764470
H	-2.763760	2.221351	1.203320
H	-0.455540	1.335212	1.364567
H	-1.574516	-1.969215	-1.190467
H	-3.871585	-1.052482	-1.345374
S	0.710307	-1.066678	0.248042
O	0.645335	-2.358643	0.878913
C	1.314000	-1.367825	-1.545870
N	1.750205	-0.127872	1.010949
N	2.007389	1.048287	0.364269
O	2.782115	1.795894	0.911080
O	1.454251	1.275955	-0.712741

N	-4.780177	1.163479	-0.180593
O	-5.562740	0.583628	-0.902740
O	-5.043663	2.153850	0.467092
F	2.588337	-1.052937	-1.600610
F	0.622927	-0.678348	-2.419709
F	1.163527	-2.656043	-1.768314

22l-anion

C	-3.316401	0.588163	-0.094021
C	-2.326307	1.272475	0.591936
C	-1.100714	0.647414	0.764486
C	-0.901540	-0.627102	0.254154
C	-1.908225	-1.299980	-0.427002
C	-3.138414	-0.686243	-0.607563
H	-2.509593	2.264678	0.979289
H	-0.299122	1.140880	1.300146
H	-1.731554	-2.297307	-0.807655
H	-3.941749	-1.184277	-1.131879
S	0.718959	-1.406727	0.516592
O	0.461433	-2.839575	0.290011
C	1.408598	-0.957934	-1.185079
N	1.515929	-0.802124	1.576141
N	-4.619789	1.244963	-0.286197
O	-5.474694	0.644791	-0.906621
O	-4.773730	2.354586	0.183891
F	2.626665	-1.491847	-1.320470
F	0.660880	-1.394680	-2.205780
F	1.533467	0.367536	-1.324033

22f

C	-3.377593	0.626164	-0.036814
C	-2.601812	0.968150	1.058167
C	-1.304808	0.489078	1.117321
C	-0.842223	-0.306892	0.077319
C	-1.621481	-0.643938	-1.017799
C	-2.921836	-0.166220	-1.074468
H	-3.000082	1.592938	1.844715
H	-0.669684	0.737989	1.959117
H	-1.244752	-1.259021	-1.823752
H	-3.561967	-0.405468	-1.911505
S	0.808124	-0.943323	0.273911
O	0.852271	-1.885561	1.371688
C	1.272042	-1.734417	-1.236487
H	1.167924	-1.046810	-2.070952
H	2.312182	-2.024146	-1.079448
H	0.637616	-2.614790	-1.338815
N	1.812647	0.265041	0.656702
N	1.846800	1.285985	-0.228286
O	2.581801	2.212664	0.059385
O	1.161271	1.254522	-1.253716
N	-4.761079	1.134933	-0.099611
O	-5.438586	0.814681	-1.053727
O	-5.143779	1.844361	0.807102

22f-anion

C	-3.326688	0.610453	-0.090997
C	-2.693125	0.251983	1.088935
C	-1.344534	-0.067031	1.037852
C	-0.665583	-0.021820	-0.173861
C	-1.320118	0.347592	-1.340684
C	-2.668837	0.668626	-1.309243
H	-3.241782	0.227916	2.020066
H	-0.813229	-0.342637	1.939860
H	-0.765638	0.390695	-2.270601
H	-3.201083	0.956298	-2.204972
S	1.124217	-0.434605	-0.266660
O	1.558367	-0.242208	1.142849
C	0.949218	-2.231642	-0.391302
H	0.466767	-2.473388	-1.337382
H	1.963758	-2.632447	-0.372513
H	0.375192	-2.614829	0.451693
N	1.782016	0.181915	-1.429564
N	-4.756679	0.943979	-0.049109
O	-5.289122	1.311492	-1.078648
O	-5.339216	0.834472	1.012523

22e

C	3.986475	1.473063	-0.511561
C	2.915679	2.246820	-0.077215
C	1.717339	1.643906	0.268368
C	1.626010	0.260983	0.153894
C	2.683035	-0.534668	-0.268989
C	3.874878	0.090548	-0.600414
H	3.011380	3.322247	-0.002441
H	0.888970	2.245764	0.620026
H	2.596118	-1.612504	-0.321856
H	4.715603	-0.507297	-0.927446
S	0.146278	-0.521236	0.708429
O	-0.100785	-0.287668	2.109566
C	-1.300289	0.292377	-0.251829
N	0.107536	-2.087314	0.405729
N	0.174629	-2.408471	-0.921434
O	0.172938	-3.590627	-1.177131
O	0.227301	-1.512165	-1.762470
F	-2.082987	-0.686495	-0.686630
F	-0.841790	1.012128	-1.262356
H	4.919871	1.953698	-0.777508
C	-2.147930	1.195906	0.673464
F	-3.060178	1.806936	-0.069250
F	-1.390930	2.111133	1.258345
F	-2.757053	0.467045	1.590692

22e-anion

C	3.756586	1.681737	-0.498939
C	3.027898	2.024701	0.634051
C	1.899844	1.291420	0.984051
C	1.515674	0.214491	0.196080
C	2.247234	-0.144098	-0.929598
C	3.367589	0.597881	-1.280234
H	3.335977	2.864007	1.245837
H	1.320809	1.545220	1.862333
H	1.941979	-1.002877	-1.515611
H	3.940300	0.327692	-2.159177
S	0.096891	-0.799235	0.665532
O	-0.274746	-0.307478	2.005390
C	-1.241856	0.089939	-0.390218
N	0.185016	-2.189281	0.232546
F	-2.296311	-0.749743	-0.465923
F	-0.779497	0.271524	-1.648191
H	4.633352	2.256915	-0.772008

C	-1.793980	1.436421	0.084346
F	-2.581110	1.972018	-0.858452
F	-0.811605	2.300857	0.322751
F	-2.523742	1.299690	1.183360

22d

C	-3.440332	0.626033	-0.123719
C	-2.494074	1.317593	0.623439
C	-1.199162	0.832860	0.727010
C	-0.896894	-0.352674	0.071869
C	-1.827252	-1.071650	-0.671074
C	-3.112202	-0.563062	-0.767158
H	-2.759687	2.237418	1.127848
H	-0.458269	1.360563	1.314579
H	-1.564847	-2.002106	-1.162292
H	-3.856643	-1.097586	-1.342600
S	0.702768	-1.060367	0.255611
O	0.662114	-2.347926	0.900925
C	1.321851	-1.387303	-1.525258
N	1.741287	-0.106921	1.008342
N	2.002434	1.061348	0.353795
O	2.756823	1.824222	0.912631
O	1.480804	1.272660	-0.741373
F	2.601332	-1.085159	-1.574731
F	0.647372	-0.704021	-2.417966
F	1.164955	-2.677873	-1.739912
H	-4.447391	1.016250	-0.203929

22d-anion

C	-3.315923	0.647871	-0.174347
C	-2.290231	1.319153	0.483000
C	-1.068981	0.688800	0.687526
C	-0.885615	-0.609049	0.230853
C	-1.905371	-1.288472	-0.424934
C	-3.125165	-0.653449	-0.626458
H	-2.442012	2.331238	0.838016
H	-0.259618	1.189837	1.205342
H	-1.742301	-2.303643	-0.763938
H	-3.927582	-1.175334	-1.133938
S	0.712194	-1.400516	0.522514
O	0.449821	-2.838124	0.320790
C	1.442435	-1.002273	-1.176409
N	1.517861	-0.793412	1.576888
F	2.658064	-1.554567	-1.272758
F	1.591191	0.318108	-1.348945
F	0.715687	-1.454366	-2.206595
H	-4.268564	1.139393	-0.332376

22b

C	-3.440444	0.583051	-0.076864
C	-2.498717	1.251061	0.697016
C	-1.198115	0.774757	0.776472
C	-0.881186	-0.377233	0.072125
C	-1.806605	-1.068652	-0.701679
C	-3.098742	-0.571269	-0.773644
H	-2.772524	2.146251	1.240287
H	-0.456972	1.286134	1.378497
H	-1.536327	-1.973548	-1.234464
H	-3.838168	-1.088440	-1.371284
S	0.741922	-1.060657	0.194465
O	0.739779	-2.317733	0.912653
C	1.264751	-1.355418	-1.545084
N	1.766660	-0.063788	0.936980
N	2.021766	1.094257	0.279231
O	2.812987	1.843592	0.816837
O	1.465810	1.340037	-0.792561
F	2.623226	-1.303718	-1.548831
H	-4.452380	0.964196	-0.136914
H	0.919847	-2.364084	-1.776739
H	0.839607	-0.590594	-2.188359

22b-anion

C	-3.303569	0.663772	-0.177346
C	-2.298948	1.306244	0.538057
C	-1.074408	0.676596	0.732679
C	-0.863253	-0.593254	0.214252
C	-1.864670	-1.241280	-0.500342
C	-3.087193	-0.608802	-0.696140
H	-2.469213	2.295663	0.945694
H	-0.280994	1.157690	1.293359
H	-1.686670	-2.237290	-0.887423
H	-3.872964	-1.110847	-1.248198
S	0.753218	-1.387760	0.478515
O	0.444230	-2.836219	0.353532
C	1.410810	-0.983397	-1.195038
N	1.546973	-0.793466	1.563712
F	2.691771	-1.503616	-1.282360
H	-4.258258	1.153544	-0.328740
H	0.793992	-1.440880	-1.968481
H	1.466224	0.100394	-1.299398

22c

C	-3.441723	0.607060	-0.121602
C	-2.502169	1.297597	0.634917
C	-1.201910	0.824354	0.731559
C	-0.885347	-0.348291	0.061436
C	-1.808937	-1.063555	-0.693208
C	-3.099941	-0.568332	-0.782705
H	-2.777404	2.207560	1.152056
H	-0.465016	1.352095	1.324372
H	-1.535696	-1.982992	-1.199403
H	-3.838235	-1.102408	-1.366606
S	0.724929	-1.045502	0.222955
O	0.691973	-2.331281	0.884894
C	1.323386	-1.396022	-1.539025
N	1.747112	-0.081941	1.001430
N	2.010524	1.082337	0.349583
O	2.767875	1.846155	0.909202
O	1.488117	1.300889	-0.744280
F	2.625269	-1.113118	-1.558629
F	0.681531	-0.652323	-2.429811
H	-4.453245	0.986876	-0.195478
H	1.145380	-2.461354	-1.700157

22c-anion

C	-3.315327	0.655841	-0.178561
C	-2.310172	1.305474	0.530229
C	-1.084041	0.680637	0.725505
C	-0.873412	-0.591427	0.212318
C	-1.873588	-1.247769	-0.495573

C	-3.098251	-0.618927	-0.690511
H	-2.481380	2.297214	0.931463
H	-0.289219	1.166671	1.279359
H	-1.694368	-2.245197	-0.877780
H	-3.884575	-1.126236	-1.236722
S	0.738330	-1.379364	0.479202
O	0.457145	-2.823782	0.303627
C	1.421096	-1.004349	-1.213696
N	1.536776	-0.779682	1.550834
F	2.652336	-1.571164	-1.292969
F	1.599601	0.336093	-1.346554
H	-4.271408	1.142754	-0.330205
H	0.797835	-1.370088	-2.030276

22a

C	-3.415399	0.611260	-0.041314
C	-2.611930	0.946122	1.044066
C	-1.309121	0.478217	1.114298
C	-0.838803	-0.322244	0.079885
C	-1.620248	-0.667746	-1.012458
C	-2.923484	-0.190990	-1.063146
H	-4.432116	0.981016	-0.090888
H	-2.998058	1.573737	1.836986
H	-0.672534	0.733860	1.953588
H	-1.238266	-1.286278	-1.813915
H	-3.550409	-0.447202	-1.907633
S	0.807075	-0.943653	0.272500
O	0.880101	-1.889365	1.368334
C	1.281757	-1.728961	-1.239411
H	1.157784	-1.047225	-2.075566
H	2.328733	-1.995406	-1.088582
H	0.666609	-2.623286	-1.335885
N	1.814276	0.268893	0.655312
N	1.850868	1.289813	-0.225556
O	2.572600	2.225304	0.076388
O	1.186892	1.255552	-1.264886

22a-anion

C	-3.331759	0.657707	-0.146463
C	-2.323821	1.294779	0.568544
C	-1.091720	0.670990	0.739052
C	-0.872011	-0.585713	0.194779
C	-1.877804	-1.227798	-0.518298
C	-3.108783	-0.604200	-0.688827
H	-4.292234	1.141666	-0.278995
H	-2.496987	2.275648	0.995720
H	-0.297276	1.149072	1.301150
H	-1.695473	-2.214662	-0.926674
H	-3.896219	-1.104628	-1.240363
S	0.760793	-1.377849	0.431125
O	0.434918	-2.826083	0.304321
C	1.468907	-0.989990	-1.190898
H	1.609476	0.087608	-1.264313
H	2.433231	-1.499220	-1.226319
H	0.820294	-1.354936	-1.986402
N	1.523698	-0.802079	1.553171

28a

C	-3.935707	-0.412671	0.323372
C	-2.874834	-1.247539	-0.001826
C	-1.624234	-0.701229	-0.261272
C	-1.470247	0.677702	-0.189420
C	-2.513270	1.526766	0.130523
C	-3.757549	0.964101	0.390875
H	-4.908567	-0.840452	0.530251
H	-3.002174	-2.322019	-0.041365
H	-2.359654	2.597316	0.173964
H	-4.589200	1.606950	0.649311
C	0.963226	-1.232026	-0.121941
S	0.081798	1.356397	-0.649160
O	0.238802	2.646500	-0.046787
O	0.296064	1.166491	-2.057104
N	1.044488	0.236105	0.246053
N	2.371072	0.687430	0.430377
O	2.759546	1.599067	-0.259774
O	3.004065	0.121615	1.286229
C	-0.473933	-1.592587	-0.568347
O	-0.648082	-2.665412	-1.087341
C	1.941334	-1.557051	-1.246958
H	2.967211	-1.374176	-0.921642
H	1.854292	-2.614573	-1.490375
H	1.731869	-0.969333	-2.140970
C	1.234801	-2.119106	1.116940
H	0.974993	-3.133332	0.806826
H	2.305884	-2.113088	1.310431
C	0.462861	-1.729951	2.368587
H	0.703884	-2.422262	3.176203
H	0.722654	-0.722943	2.698625
H	-0.617782	-1.770247	2.212286

28a-anion

C	-3.985296	-0.346878	0.275246
C	-2.924784	-1.208499	0.035886
C	-1.648884	-0.697058	-0.192276
C	-1.455497	0.683967	-0.186250
C	-2.510172	1.550959	0.047410
C	-3.777620	1.030012	0.281413
H	-4.974956	-0.745905	0.460701
H	-3.070343	-2.282053	0.034753
H	-2.336327	2.620071	0.052729
H	-4.606776	1.700646	0.472868
C	0.933569	-1.186867	-0.107688
S	0.210574	1.239398	-0.505231
O	0.346141	2.574571	0.068468
O	0.313362	1.193150	-1.970468
N	1.085369	0.220792	0.273854
C	-0.506691	-1.631015	-0.432353
O	-0.721239	-2.755569	-0.836233
C	1.831757	-1.554782	-1.297256
H	2.867498	-1.332591	-1.031451
H	1.747407	-2.615290	-1.538139
H	1.564641	-0.963099	-2.172950
C	1.363778	-2.035099	1.111387
H	1.394369	-3.083264	0.803589
H	2.386713	-1.730284	1.349531
C	0.474980	-1.879045	2.338983
H	0.892883	-2.425973	3.186394
H	0.384488	-0.829869	2.625406
H	-0.530017	-2.272095	2.161413

29a

C	-4.001051	-0.960380	-0.259768
C	-2.742593	-0.974292	0.330866
C	-2.017963	0.201519	0.472620
C	-2.601729	1.375274	0.007305
C	-3.842957	1.412861	-0.609553
C	-4.548773	0.224754	-0.737123
H	-0.573702	0.727821	1.961576
H	-4.550932	-1.887623	-0.365497
H	-2.303963	-1.907640	0.664878
C	-0.609718	0.242377	0.981863
H	-4.245992	2.345420	-0.983441
H	-5.520919	0.226792	-1.212769
C	0.321941	1.012967	0.037627
S	-1.781622	2.910932	0.257732
O	-1.865184	3.287121	1.643427
O	-2.185141	3.834801	-0.762005
N	-0.128331	2.435259	-0.046941
H	-0.226067	-0.767655	1.110401
H	1.291268	1.054294	0.531680
N	0.780145	3.426834	0.267940
O	0.343182	4.556987	0.355501
O	1.942789	3.114781	0.401416
C	0.562970	0.429845	-1.381831
C	1.739602	1.187516	-2.009461
H	2.636909	1.113988	-1.391298
H	1.960214	0.761982	-2.990642
H	1.505691	2.245738	-2.154192
C	-0.634776	0.552035	-2.330983
H	-1.472836	-0.081858	-2.039270
H	-0.985675	1.585039	-2.408167
H	-0.317691	0.239487	-3.328929
C	0.945696	-1.044937	-1.235754
H	1.757399	-1.176230	-0.514576
H	0.095117	-1.651702	-0.916823
H	1.285393	-1.428523	-2.200230

29a-anion

C	-4.502771	-0.706282	0.062911
C	-3.167438	-0.932311	0.359945
C	-2.224855	0.098261	0.299603
C	-2.671757	1.362960	-0.074155
C	-4.007593	1.597655	-0.385269
C	-4.929422	0.566000	-0.311874
H	-0.703585	-0.122565	1.751648
H	-5.213397	-1.522531	0.118842
H	-2.838625	-1.927040	0.642658
C	-0.784168	-0.176936	0.660529
H	-4.319275	2.592889	-0.681513
H	-5.971215	0.748523	-0.546350
C	0.244496	0.806414	0.074242
S	-1.509392	2.721408	-0.184913
O	-2.013378	3.789140	0.688795
O	-1.550382	3.118539	-1.603540
N	-0.158879	2.189266	0.358011
H	-0.547854	-1.207341	0.385484
H	1.154513	0.676127	0.669809
C	0.701509	0.455169	-1.378699
C	1.485069	1.634284	-1.957599
H	2.294611	1.933953	-1.284843
H	1.929625	1.352535	-2.916924
H	0.831982	2.494105	-2.109985
C	-0.447998	0.101479	-2.328489
H	-0.982049	-0.794035	-1.998661
H	-1.159711	0.921774	-2.424074
H	-0.041122	-0.108058	-3.322877
C	1.643475	-0.754715	-1.302541
H	2.527735	-0.527375	-0.700309
H	1.153771	-1.629253	-0.866804
H	1.981580	-1.032820	-2.304547

29b

C	-3.936535	-0.453912	0.333671
C	-2.867918	-1.273745	0.003413
C	-1.627003	-0.712668	-0.264742
C	-1.476141	0.666254	-0.199263
C	-2.514755	1.512606	0.128914
C	-3.734695	0.913767	0.390526
H	-4.910185	-0.871385	0.549785
H	-2.990941	-2.348284	-0.033391
H	-2.386428	2.585699	0.177669
C	0.964631	-1.231100	-0.126861
S	0.078560	1.350982	-0.660663
O	0.219245	2.643956	-0.064232
O	0.285633	1.148402	-2.066073
N	1.031034	0.237219	0.246162
N	2.355313	0.697805	0.447772
O	2.746294	1.606503	-0.243874
O	2.976489	0.139450	1.315131
C	-0.465209	-1.595505	-0.585235
O	-0.639863	-2.654206	-1.127151
C	1.953626	-1.548324	-1.244266
H	2.975007	-1.360096	-0.908302
H	1.875680	-2.606230	-1.489201
H	1.748926	-0.961118	-2.139556
C	1.231042	-2.120598	1.112124
H	0.985780	-3.136082	0.793910
H	2.300338	-2.103998	1.314699
C	0.443163	-1.747051	2.358528
H	0.698597	-2.431280	3.168442
H	0.675431	-0.733076	2.687659
H	-0.635553	-1.817020	2.198575
N	-4.870038	1.783379	0.751497
O	-4.658854	2.973982	0.842513
O	-5.946049	1.256150	0.935234

29b-anion

C	-3.977471	-0.381255	0.336104
C	-2.913388	-1.225686	0.065342
C	-1.650241	-0.697873	-0.184888
C	-1.458436	0.682813	-0.171157
C	-2.503995	1.545978	0.096594
C	-3.745396	0.985393	0.346214
H	-4.964857	-0.770498	0.540008
H	-3.055121	-2.298900	0.057266
H	-2.354285	2.616898	0.114740
C	0.935674	-1.182253	-0.118387
S	0.207244	1.241772	-0.508896
O	0.335121	2.578076	0.058124
O	0.287250	1.180020	-1.972540

N	1.078537	0.224805	0.270594
C	-0.499946	-1.623400	-0.458908
O	-0.720770	-2.729870	-0.899849
C	1.847061	-1.543803	-1.298834
H	2.878461	-1.317647	-1.020846
H	1.770059	-2.604198	-1.542141
H	1.586251	-0.952306	-2.176427
C	1.349989	-2.035269	1.103464
H	1.381978	-3.082315	0.791839
H	2.370887	-1.732907	1.352233
C	0.448498	-1.880953	2.322074
H	0.858763	-2.428963	3.172309
H	0.354463	-0.832371	2.609465
H	-0.554300	-2.275274	2.134764
N	-4.872560	1.886974	0.638963
O	-4.645108	3.078068	0.705379
O	-5.970758	1.392425	0.796875

28b

C	-4.025896	-0.966511	-0.198328
C	-2.760673	-0.978954	0.367419
C	-2.026716	0.195117	0.486300
C	-2.606453	1.376694	0.030910
C	-3.851687	1.428005	-0.564451
C	-4.541380	0.233056	-0.661045
H	-0.576010	0.709296	1.961663
H	-4.598978	-1.878219	-0.295769
H	-2.326669	-1.913311	0.701515
C	-0.614787	0.229266	0.979116
H	-4.269418	2.351402	-0.942599
C	0.309538	1.006026	0.031952
S	-1.777729	2.915332	0.274397
O	-1.863973	3.282392	1.660516
O	-2.193885	3.831242	-0.745444
N	-0.136757	2.430255	-0.039822
H	-0.232947	-0.782065	1.099878
H	1.281254	1.041702	0.521912
N	0.778357	3.420200	0.275342
O	0.341436	4.549060	0.369612
O	1.938709	3.103237	0.400744
C	0.540226	0.432047	-1.392364
C	1.705775	1.201107	-2.026506
H	2.609258	1.129992	-1.417233
H	1.919610	0.781779	-3.011699
H	1.463145	2.258365	-2.164090
C	-0.668417	0.552319	-2.327480
H	-1.496639	-0.094055	-2.034922
H	-1.030082	1.582501	-2.393165
H	-0.359252	0.251587	-3.331326
C	0.932695	-1.041172	-1.257458
H	1.751143	-1.171289	-0.543839
H	0.088335	-1.654956	-0.935404
H	1.266362	-1.416874	-2.226970
N	-5.869240	0.243224	-1.289500
O	-6.312916	1.311181	-1.659381
O	-6.449380	-0.816956	-1.404691

28b-anion

C	-4.468508	-0.837512	-0.050958
C	-3.138135	-1.016324	0.275007
C	-2.227437	0.045575	0.237034
C	-2.694904	1.301136	-0.150559
C	-4.022170	1.505090	-0.492579
C	-4.888903	0.430903	-0.429068
H	-0.755072	-0.117483	1.731530
H	-5.170158	-1.659089	-0.017071
H	-2.789236	-2.000286	0.566449
C	-0.793970	-0.186914	0.638846
H	-4.372777	2.480869	-0.802049
C	0.226065	0.817775	0.074393
S	-1.561954	2.692582	-0.245055
O	-2.122693	3.747570	0.604696
O	-1.586494	3.065532	-1.668003
N	-0.220377	2.191067	0.336329
H	-0.525189	-1.214477	0.385576
H	1.119617	0.714293	0.698519
C	0.730479	0.465743	-1.361571
C	1.500237	1.659492	-1.928186
H	2.282131	1.984580	-1.234887
H	1.979026	1.382064	-2.871898
H	0.831286	2.502120	-2.106032
C	-0.385531	0.075427	-2.336177
H	-0.905035	-0.830927	-2.012094
H	-1.115304	0.875953	-2.459117
H	0.050565	-0.132343	-3.318201
C	1.698592	-0.719975	-1.248261
H	2.558878	-0.467088	-0.622098
H	1.217808	-1.603645	-0.821131
H	2.072567	-0.996204	-2.237836
N	-6.297057	0.636788	-0.774856
O	-6.655636	1.758084	-1.081268
O	-7.041958	-0.324724	-0.738702

37

C	-3.869455	0.129342	0.163148
F	-3.314281	-1.051661	0.303583
S	-3.421960	1.205969	1.611987
O	-3.852429	2.533113	1.344696
O	-1.803902	1.071759	1.279461
F	-3.466708	0.701638	-0.947224
F	-5.181332	0.023368	0.176042
O	-3.722410	0.504701	2.810111
N	-0.909512	1.782868	2.165038
O	-1.420776	2.380035	3.053977
O	0.213594	1.641461	1.841611

37-anion

C	-3.908004	0.141935	0.174019
F	-3.472925	-1.119462	0.219011
S	-3.234491	1.129336	1.574461
O	-3.814618	2.440528	1.345577
O	-1.800234	1.038104	1.366242
F	-3.539598	0.656337	-1.001508
F	-5.242554	0.111960	0.202923
O	-3.741290	0.418291	2.734910

$NO_2^+(MeCN)_4BF_4^-$

N	-2.112627	0.012892	-3.577332
O	-2.209679	-1.084209	-3.553025
O	-2.010519	1.113447	-3.600320

C	-2.442025	-0.004981	0.132904
N	-2.203415	0.053855	-0.986076
C	-2.748332	-0.080891	1.551322
H	-1.850112	-0.362900	2.099559
H	-3.524654	-0.828505	1.710569
H	-3.098839	0.891010	1.896711
C	-2.121250	-0.259754	-7.296272
N	-2.114713	-0.074679	-6.165709
C	-2.127842	-0.496902	-8.730081
H	-3.157690	-0.548198	-9.081401
H	-1.622234	-1.438829	-8.940256
H	-1.609066	0.318515	-9.232774
C	-5.800396	0.122754	-3.878833
N	-4.684152	0.276148	-3.671399
C	-7.215883	-0.067701	-4.147499
H	-7.688521	-0.524918	-3.279409
H	-7.329975	-0.722431	-5.010468
H	-7.677507	0.897907	-4.351931
C	1.602214	-0.455949	-3.564054
N	0.475615	-0.249877	-3.593078
C	3.030986	-0.718473	-3.524505
H	3.410766	-0.798181	-4.542421
H	3.209853	-1.652550	-2.992977
H	3.533088	0.099278	-3.008796
B	-4.691599	-3.224316	-3.531891
F	-4.092369	-2.673991	-2.394022
F	-6.024311	-2.806172	-3.611956
F	-4.000242	-2.811384	-4.676389
F	-4.650467	-4.621716	-3.445643

BF_4^-

B	-0.807209	-3.058539	-1.203845
F	0.511594	-3.058547	-1.670748
F	-1.465994	-1.916204	-1.670748
F	-1.465988	-4.200880	-1.670759
F	-0.806791	-3.058535	0.195137

$NO_2^+(MeCN)_4$

N	-2.501107	-0.087071	-3.657132
O	-2.691598	-1.173385	-3.628146
O	-2.310915	0.999313	-3.685410
C	-2.687895	0.013460	0.070537
N	-2.586803	-0.010346	-1.070392
C	-2.816664	0.043267	1.517545
H	-1.828105	0.143851	1.964414
H	-3.280852	-0.882234	1.856568
H	-3.436511	0.891854	1.805107
C	-2.290626	-0.191958	-7.382579
N	-2.399854	-0.167309	-6.242418
C	-2.151760	-0.222867	-8.828648
H	-3.137207	-0.323792	-9.282293
H	-1.529952	-1.071652	-9.111321
H	-1.685204	0.702370	-9.165135
C	-6.162009	0.630245	-3.798799
N	-5.047016	0.376453	-3.728923
C	-7.575992	0.952382	-3.889154
H	-7.984799	1.062069	-2.885274
H	-8.094792	0.148880	-4.410791
H	-7.697118	1.885419	-4.438397
C	1.157558	-0.822881	-3.576796
N	0.044518	-0.554593	-3.618862
C	2.568672	-1.164554	-3.521535
H	2.892173	-1.518099	-4.499871
H	2.719282	-1.948491	-2.780136
H	3.141962	-0.281007	-3.242944

MeCN

C	-2.343109	-0.162840	-7.289268
N	-2.342762	-0.162847	-6.142480
C	-2.343018	-0.162829	-8.744314
H	-3.370513	-0.162867	-9.105969
H	-1.829161	-1.052973	-9.105738
H	-1.829131	0.727291	-9.105732
